# Caspase‐8 in endothelial cells maintains gut homeostasis and prevents small bowel inflammation in mice

**DOI:** 10.15252/emmm.202114121

**Published:** 2022-05-02

**Authors:** Nathalie Tisch, Carolin Mogler, Ana Stojanovic, Robert Luck, Emilia A Korhonen, Alexander Ellerkmann, Heike Adler, Mahak Singhal, Géza Schermann, Lena Erkert, Jay V Patankar, Andromachi Karakatsani, Anna‐Lena Scherr, Yaron Fuchs, Adelheid Cerwenka, Stefan Wirtz, Bruno Christian Köhler, Hellmut G Augustin, Christoph Becker, Thomas Schmidt, Carmen Ruiz de Almodóvar

**Affiliations:** ^1^ European Center for Angioscience (ECAS) Medical Faculty Mannheim Heidelberg University Mannheim Germany; ^2^ Institute of Pathology TUM School of Medicine Technical University of Munich Munich Germany; ^3^ Department of Immunobiochemistry Mannheim Institute for Innate Immunoscience (MI3) Medical Faculty Mannheim Heidelberg University Mannheim Germany; ^4^ Division of Vascular Oncology and Metastasis German Cancer Research Center Heidelberg (DKFZ‐ZMBH Alliance) Heidelberg Germany; ^5^ Department of General, Visceral and Transplantation Surgery Heidelberg University Heidelberg Germany; ^6^ Department of Medicine 1 Friedrich‐Alexander‐University Erlangen Germany; ^7^ National Center for Tumor Diseases Department of Medical Oncology Internal Medicine VI Heidelberg University Hospital Heidelberg Germany; ^8^ Laboratory of Stem Cell Biology & Regenerative Medicine Department of Biology Technion –Israel Institute of Technology Haifa Israel; ^9^ Department of General, Visceral, Cancer and Transplantation Surgery Faculty of Medicine with University Hospital Cologne University of Cologne Cologne Germany; ^10^ Present address: Institute for Neurovascular Cell Biology University Hospital Bonn Bonn Germany; ^11^ Present address: Schlegel Chair for Neurovascular Cell Biology University of Bonn Bonn Germany

**Keywords:** caspase‐8, chronic intestinal inflammation, endothelium, necroptosis, vascular homeostasis, Digestive System, Vascular Biology & Angiogenesis

## Abstract

The gut has a specific vascular barrier that controls trafficking of antigens and microbiota into the bloodstream. However, the molecular mechanisms regulating the maintenance of this vascular barrier remain elusive. Here, we identified Caspase‐8 as a pro‐survival factor in mature intestinal endothelial cells that is required to actively maintain vascular homeostasis in the small intestine in an organ‐specific manner. In particular, we find that deletion of Caspase‐8 in endothelial cells results in small intestinal hemorrhages and bowel inflammation, while all other organs remained unaffected. We also show that Caspase‐8 seems to be particularly needed in lymphatic endothelial cells to maintain gut homeostasis. Our work demonstrates that endothelial cell dysfunction, leading to the breakdown of the gut‐vascular barrier, is an active driver of chronic small intestinal inflammation, highlighting the role of the intestinal vasculature as a safeguard of organ function.

## Graphical abstract

Inflammatory bowel disease (IBD) is a group of disorders that causes severe inflammation of the intestine. This study identified that vascular dysfunction in the small intestine results in classic features of IBD in mice.

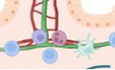

**The paper explained**

**Problem**

The gut has a specific vascular barrier that controls trafficking of antigens and microbiota into the blood stream, and as such contributes to gut homeostasis. Vascular dysfunction has been reported in patients with inflammatory bowel disease (IBD). However, the molecular mechanisms that maintain the gut‐vascular barrier remain elusive. Furthermore, it remains unknown whether vascular dysfunction is only consequence, or can even be cause of small bowel inflammation.

**Results**

Our study addresses the role of Caspase‐8 (Casp8), a key pro‐survival factor in the necroptosis cell death signaling pathway, in endothelial cells (ECs). Loss of Casp8 function in epithelial cells has been shown to be involved in human IBD patients as well as in mouse models of inflammatory bowel diseases. Our study shows that EC‐specific Casp8 knockout mice recapitulate those phenotypes, indicating the additional need of endothelial Casp8 for vascular homeostasis. In particular, lymphatic EC homeostasis largely depends on Casp8 expression. Overall, our data shows that the intestinal vasculature can be a primary driver for small bowel inflammation.

**Impact**

Our study reveals that targeting cell death signaling in the intestinal vasculature in inflammatory bowel diseases might prove useful as novel therapeutical strategy to treat IBD patients.

## Introduction

The intestinal epithelium is a single‐cell layer lining the small and large intestine that constitutes the body’s largest barrier against the external environment (Martini *et al*, 2017). The gut microbiota lives in symbiosis with its host and regulates a number of physiological processes, such as integrity and permeability of the gut epithelium, host immunity, and defense against ingested pathogens (Backhed *et al*, 2005). However, the gut microbiota also poses a potential threat to the host and can lead to inflammation and infection, when immune or epithelial cell homeostasis is compromised (Goto & Kiyono, 2012; Kamada *et al*, 2013). Therefore, the intestine has acquired a highly specialized immune system. Hereby, Peyer’s patches, organized lymphoid follicles, are important players in immune surveillance (Jung *et al*, 2010; Morikawa *et al*, 2016).

The gut epithelium is functionally supported by a cooperative dense vascular network that builds a second barrier, termed the gut‐vascular barrier (GVB) (Spadoni *et al*, 2015). The GVB is intermingled by blunt‐ended lacteals, the capillaries of the lymphatic system that reside within intestinal villi (Spadoni *et al*, 2015; Bernier‐Latmani & Petrova, 2017), and both systems actively co‐regulate each other (Jang *et al*, 2013).

Accumulating evidence indicates that endothelial cells (ECs) forming the inner lining of blood vessels actively contribute to both organ development, maintenance, and repair by the secretion of tissue‐specific, so called angiocrine factors that instruct and guide cells in the environment (Rafii *et al*, 2016; Augustin & Koh, 2017). In line, it is recognized that ECs possess organ‐specific morphological and transcriptional properties that are required to develop organotypic properties (Nolan *et al*, 2013). Similarly, organ‐specific lymphatic ECs (LECs) exist (Bernier‐Latmani & Petrova, 2017; Oliver *et al*, 2020). However, the molecular mechanisms that confer these organotypic features and that maintain vascular homeostasis in an organ‐specific manner remain elusive.

Inflammatory bowel diseases (IBD) are chronic inflammatory disorders of the small and large intestine (Kim *et al*, 2017; Martini *et al*, 2017). IBD pathogenesis is not just caused by immune cell‐mediated mechanisms but is also closely associated with a loss of epithelial barrier integrity that can lead to excessive translocation of commensal microbiota followed by an exaggerated immune response (Martini *et al*, 2017). Even though vascular impairments in the intestine have been reported in IBD patients (Homan *et al*, 1976; Dvorak *et al*, 1980; Wakefield *et al*, 1991), only little is known about the mechanisms that might lead to gut‐specific vascular dysfunction. Whereas it is assumed that these vascular defects arise secondary to increased intestinal inflammation, the possibility that vascular dyshomeostasis can be a primary cause for IBD development has so far not been sufficiently investigated.

Caspase‐8 (Casp8) is a central regulator of the extrinsic cell death signaling pathway. When fully activated, Casp8 leads to the proteolytic cleavage and activation of the executioner Caspase‐3 to induce cellular apoptosis (Lin *et al*, 1999; Kang *et al*, 2004). On the other hand, restricted Casp8 activity is required to prevent necroptosis (Oberst *et al*, 2011; Dillon *et al*, 2012). Loss of Casp8, or inhibition of its enzymatic activity, results in the formation of the RIPK1‐RIPK3 comprising necrosome and the phosphorylation of mixed lineage kinase protein like (MLKL), the ultimate executer of necroptosis (Sun *et al*, 2012; Zhao *et al*, 2012).

Interestingly, a subpopulation of human patients with a CASP8 deficiency suffering from very early onset IBD has been identified (Lehle *et al*, 2019). Epithelial cell‐specific knockout of Casp8 in adult mice results in mouse lethality due to massive intestinal defects with similar features as seen in human IBD conditions (Gunther *et al*, 2011; Schwarzer *et al*, 2020). This is caused by epithelial cell necroptosis (Gunther *et al*, 2011), indicating that Casp8 is an important pro‐survival factor of the gut‐epithelial barrier. Epithelial cell death in the absence of Casp8 can be inhibited if cytokine signaling is downregulated (Gunther *et al*, 2011, 2019), and colonic inflammation in epithelial cell‐specific Casp8 knockout mice strongly depends on microbial composition (Stolzer *et al*, 2020). Whether Casp8 is required in a similar fashion as a pro‐survival factor of the GVB, and thus, whether EC dysfunction in the absence of Casp8 in the gut might also contribute to IBD development, remains elusive.

In this study, we describe that acute deletion of Casp8 in ECs of adult mice (from hereon Casp8^ECko^) resulted in a gut‐vasculature‐specific phenotype and caused lethality of the mice around 3 weeks after the first tamoxifen treatment. Most strikingly, Casp8^ECko^ mice developed severe inflammation and tissue damage. These defects were dependent on EC necroptosis and the presence of microbiota. Importantly, deletion of Casp8 solely in blood ECs in the intestine did not result in disease, thus highlighting the importance of Casp8 expression primarily in the lymphatic vasculature. We therefore identified Casp8 as a novel, organotypic regulator of vascular homeostasis in the small intestine. Furthermore, our data suggests that the intestinal endothelium acts as an important safeguard of overall intestine homeostasis as vascular dysfunction can be a primary driver for intestine inflammation.

## Results

### Acute loss of Casp8 in adult ECs is lethal and impairs intestine homeostasis

**Figure 1:**
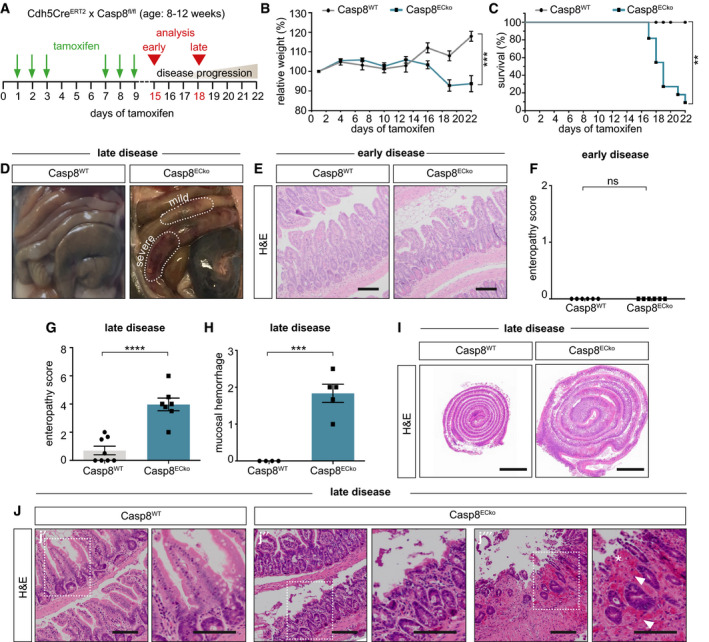
Acute loss of Casp8 in adult ECs is lethal and impairs intestine homeostasis ASchematic representation of Casp8 deletion in adult ECs indicating the time points for tamoxifen treatment, analysis of the phenotype (early (day 15) or late (day 18)), and the timeframe of disease progression.B, CGraphs showing the relative weight (B; *n* = 15 WT, 12 ECko; curve comparison) and the survival (C; *n* = 15 WT, 11 ECko; Log‐Rank test) of mice upon tamoxifen treatment.DRepresentative images of the intestine of Casp8^WT^ and Casp8^ECko^ mice, with mild and severe hemorrhages at a late disease stage.E, FRepresentative images of H&E staining (E) and quantification of intestinal pathology (F) of the small intestine at an early disease stage in Casp8^WT^ and Casp8^ECko^ mice (enteropathy score; *n* = 6 WT, 6 ECko; two‐tailed unpaired Student’s *t*‐test). Scale bars: 100 µm; insets 50 µm.G, HQuantification of intestinal pathology (enteropathy score, G; *n* = 8 WT, 7 ECko; two‐tailed unpaired Student’s *t*‐test) and hemorrhages in the mucosa (H; *n* = 4 WT, 5 ECko; two‐tailed unpaired Student’s *t*‐test) at a late disease stage in Casp8^WT^ and Casp8^ECko^ mice.IOverview of H&E‐stained Swiss roll preparations of Casp8^WT^ and Casp8^ECko^ mice. Scale bars: 2 mm.JRepresentative images of H&E staining of the small intestine of Casp8^WT^ (j’) and Casp8^ECko^ mice with mild (j″) and more severely (j‴) affected areas at a late disease stage (white arrows point to hemorrhages, asterisk points to focal erosion of the epithelium). Scale bars: 100 µm. Data information: Data shown as mean ± SEM. **P < 0.01, ***P < 0.001, ****P < 0.0001, ns: not significant. Source data are available online for this figure.

**Figure EV1:**
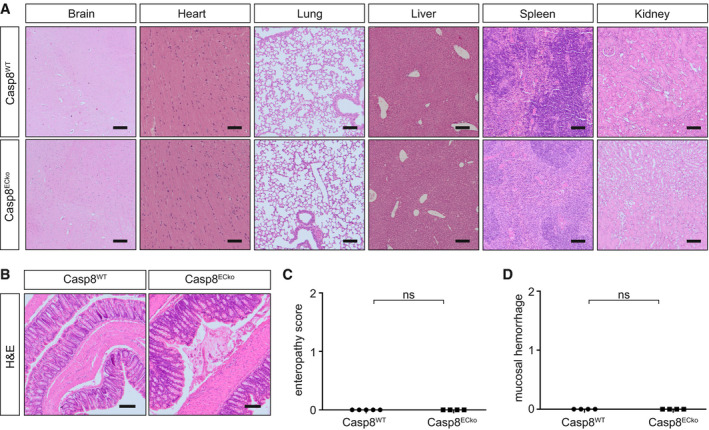
Casp8^ECko^ mice do not present defects in other organs ARepresentative images of H&E staining of the indicated organs in Casp8^WT^ and Casp8^ECko^ mice at a late disease stage (*n* = 4–5 WT, 5 ECko). Scale bars 100 µm.B–DRepresentative images of H&E staining of the large intestine (colon) (B). Quantification of intestinal pathology (C) and mucosal hemorrhages (D) in Casp8^WT^ and Casp8^ECko^ mice at a late disease stage (enteropathy score; *n* = 4 WT, 4 ECko; two‐tailed unpaired Student’s *t*‐test). Scale bars: 100 µm. Data information: All data is shown as mean ± SEM. ns: not significant. Source data are available online for this figure.

To determine whether Casp8 is required in ECs to maintain vessel homeostasis, we induced the deletion of Casp8 in adult ECs using an EC‐specific Casp8 floxed line (Cdh5Cre^ERT2^; Casp8^fl/fl^ mice; termed from here on Casp8^ECko^ (Tisch *et al*, 2019)) (Fig 1A). Around 3 weeks after the first tamoxifen treatment, Casp8^ECko^ mice had significantly reduced weight compared to Casp8^fl/fl^ (from here on Casp8^WT^ mice) (Fig 1B) and ~90% of the Casp8^ECko^ mice died or had to be euthanized due to illness (Fig 1C). Surprisingly, examination of all main organs only revealed hemorrhages in the small intestine (Fig 1D), whereas all others seemed unaffected (Fig EV1A).

To characterize the intestinal defects in Casp8^ECko^ mice in more detail, we scored the grade of inflammation and epithelial cell damage using standard histopathology (Welz *et al*, 2011) at different disease stages. At 15 days after the first tamoxifen treatment (early disease), Casp8^ECko^ mice did not present obvious histopathological changes (Fig 1E and F). However, starting at 18 days after the first tamoxifen treatment (late disease), Casp8^ECko^ mice developed profound enteritis that most strongly manifested in the terminal ileum (Fig 1G) and mucosal hemorrhages (Fig 1H). Swiss roll preparations of the small intestine of Casp8^ECko^ mice were larger, probably due to cell infiltration and fluid accumulation (Fig 1I). Compared to Casp8^WT^ mice (Fig 1J and j´), Casp8^ECko^ mice presented mild (Fig 1J and j″) to severe (Fig 1J and j‴) tissue lesions that were accompanied by inflammation and focal erosion of the epithelium in hypoxic‐necrotic tissue. The large intestine was not affected (Fig EV1B–D).

This phenotype was not due to a restricted expression of Cre recombinase in gut ECs as analysis of Cdh5‐Cre^ERT2^ x Rosa^mTmG^ reporter mice revealed active Cre recombinase in ECs of all organs (Appendix Fig S1A and B).

Furthermore, Casp8 expression was not only reduced in isolated intestine ECs but also in brain, lung, and skin ECs of Casp8^ECko^ mice (Appendix Fig S1C–G). We also ruled out that the Cdh5‐Cre^ERT2^ was unspecifically active in the intestinal epithelium, as Casp8 mRNA levels in isolated intestinal epithelial cells of Casp8^ECko^ mice were equal to controls (Appendix Fig S1H). In addition, GFP^+^ cells in Cdh5‐Cre^ERT2^ x Rosa^mTmG^ mice only colocalized with blood (CD31^+^) and lymphatic (CD31^+^ Lyve‐1^+^) ECs, but never with the intestinal epithelium (Appendix Fig S1I). Together, these findings indicate that deletion of Casp8 in ECs drives a severe intestinal pathology, without affecting other organs.

### Loss of Casp8 in ECs leads to small bowel inflammation

**Figure 2:**
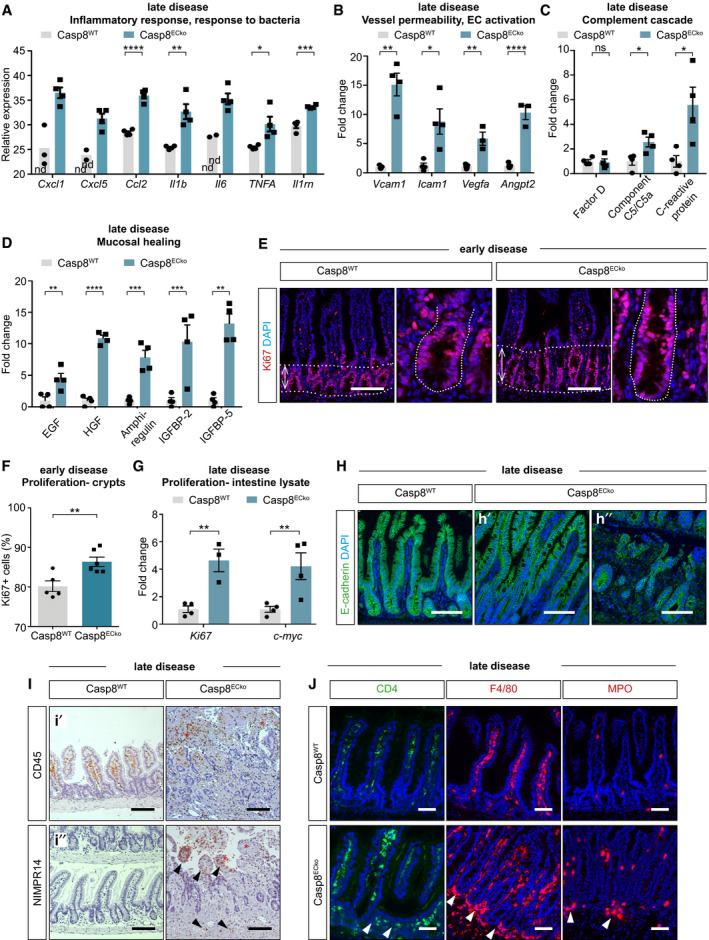
Loss of Casp8 in ECs leads to small bowel inflammation A, BQPCR analysis of genes associated to immune and bacterial response (A), vessel permeability, and EC activation (B) in small intestine lysates at a late disease timepoint in Casp8^WT^ and Casp8^ECko^ mice (*n* = 4 WT, 3‐4 ECko, biological replicates, multiple *t*‐test with Holm–Sidak method).C, DGraphs revealing that significantly upregulated cytokines in the proteome profiler in Casp8^ECko^ mice are associated to the complement cascade (C) and mucosal healing (D) (*n* = 4 WT, 4 ECko, multiple *t*‐test with Holm–Sidak method).E, FRepresentative images (E; area between dotted lines shows crypts) and quantification (F) of Ki67 staining in small intestine at an early disease stage in Casp8^WT^ and Casp8^ECko^ mice (*n* = 5 WT, 6 ECko two‐tailed unpaired Student’s *t*‐test). Scale bars: 100 µm.GQPCR analysis of the proliferation markers Ki67 and c‐myc in ileal lysates from late disease stage Casp8^WT^ and Casp8^ECko^ mice (*n* = 4 WT, 3‐4ECko, two‐way ANOVA with Sidak method).HRepresentative images of E‐Cadherin staining, showing intact epithelial barrier integrity in Casp8^ECko^ mice in healthy areas (h′), and disrupted barrier integrity in intestinal lesions (h″) at late disease stages compared to Casp8^WT^ mice (*n* = 4 WT, 4 ECko). Scale bars: 100 µm.IRepresentative pictures of CD45 and NIMPR14 staining at late disease stages (*n* = 5 WT, 5 ECko), showing accumulation of CD45^+^ (i´) and NIMPR14^+^ (i´´) cells (black arrowheads) in Casp8^ECko^ intestine at sites of impaired epithelial barrier integrity and tissue necrosis (red asterisks). Scale bars: 100 µm.JRepresentative images of CD4, F4/80, and MPO stainings in small intestines at a late disease stage showing accumulation of these cells at the crypt base in Casp8^ECko^ mice (arrowheads) (*n* = 4 WT, 4 ECko). Scale bars: 50 µm. Data information: Data shown as mean ± SEM. *P < 0.05, **P < 0.01, ***P < 0.001, ****P < 0.0001. Source data are available online for this figure.

**Figure EV2:**
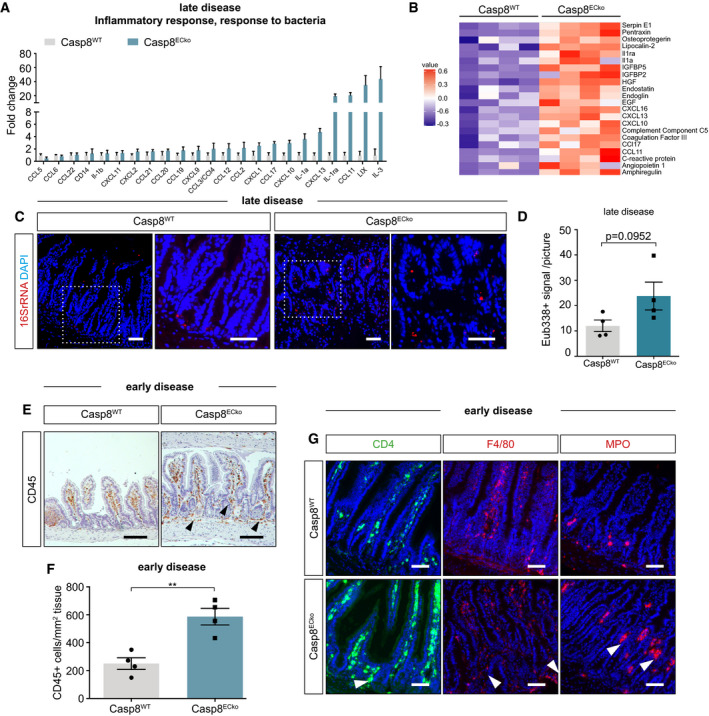
Loss of Casp8 in ECs leads to small bowel inflammation AGraph showing increased expression of proteins associated to the GOs inflammatory response and bacterial response in small intestinal samples of Casp8^ECko^ mice (*n* = 4 WT, 4 ECko), identified via a proteome profiler analysis.BRow‐normalized heat map of significantly upregulated genes in the proteome profiler analysis of small intestinal samples of Casp8^ECko^ compared to Casp8^WT^ mice (*n* = 4 WT, 4 ECko, unpaired students *t*‐test, unadjusted *P*‐values).C, DRepresentative pictures (C) and quantification (D) of bacterial 16S rRNA in Casp8^WT^ and Casp8^ECko^ mice by *FISH* at a late disease stage (*n* = 4 WT, 4 ECko two‐tailed unpaired student *t*‐test). Scale bars: 50 µm.E, FRepresentative pictures (E) and quantification (F) of CD45^+^ cells in deep layers of the lamina in the small intestine of Casp8^WT^ and Casp8^ECko^ mice (*n* = 4 WT, 4 ECko, two‐tailed unpaired student *t*‐test. Black arrowheads point to accumulated CD45^+^ cells). Scale bars: 50 µm.GRepresentative pictures of T‐cells (CD4^+^), macrophages (F4/80^+^), and granulocytes (MPO^+^) in the small intestine of Casp8^WT^ and Casp8^ECko^ mice, showing accumulation of these cells in the crypt area (white arrowheads) in Casp8^ECko^ mice already at early disease stages. Scale bars: 50 µm. Data information: All data is shown as mean ± SEM. **P < 0.01, ns: not significant. Source data are available online for this figure.

We next characterized intestinal inflammation in Casp8^ECko^ mice in detail using an unbiased proteome profiler array to detect a panel of cytokines, chemokines, and growth factors in ileal tissue lysates at an advanced disease stage. Upregulated cytokines in Casp8^ECko^ mice were linked to inflammatory and bacterial response pathways, as confirmed by qPCR analysis (Figs 2A and EV2A). In line with this inflammatory profile and the presence of hemorrhages, vessel permeability and EC activation markers were strongly expressed in Casp8^ECko^ mice (Fig 2B). Cytokines involved in the complement cascade and mucosal healing were also significantly upregulated in the proteome profiler assay (Figs 2C and D, and EV2B), suggesting that active bacterial defense and compensatory mucosal repair processes to maintain tissue homeostasis were activated. Consistently, intestinal crypts were elongated and presented an increased amount of Ki67^+^ proliferating epithelial cells (Fig 2E and F), together with increased Ki67 and c‐myc expression in ileal lysates (Fig 2G). E‐cadherin staining of the epithelial lining showed that the intestinal barrier remained largely intact (Fig 2H and h′), except of local barrier breakdown at the site of severe intestinal lesions (Fig 2H and h″). In line, as shown by *FISH*‐staining of bacterial *16S rRNA* (Fig EV2C and D), bacterial translocation was restricted to the local area of intestinal lesions, which furthermore supports our above‐mentioned findings.

Importantly, immune cell infiltration could already be observed in Casp8^ECko^ guts at the earlier disease time point when mice were still overall healthy and without intestinal pathology based on the histopathological score (see Fig 1E). At this early stage, increased numbers of leukocytes (CD45^+^ cells) accumulated in the deeper layers of the lamina propria surrounding the crypt region (Fig EV2E and F), comprising T‐cells (CD4^+^), macrophages (F4/ 80^+^), and MPO‐expressing granulocytes (Fig EV2G). At late disease stages, when severe intestinal pathology was observed, CD45^+^ cells accumulated at lesions (Fig 2I and i′). In addition, and mainly at sites of impaired epithelial barrier integrity, we found a strong infiltration of NIMP‐R14^+^ neutrophils (Fig 2I and i″), known to contribute to the antimicrobial defense response upon bacterial translocation over the epithelial barrier, as well as to promote mucosal healing during chronic intestinal inflammation (Fournier & Parkos, 2012). T‐cells, macrophages, and granulocytes further accumulated at the crypt base in Casp8^ECko^ (Fig 2J). Altogether, the sole deletion of Casp8 in ECs promoted early immune cell activation in the intestine with the subsequent progression to features of chronic intestinal inflammation (reminiscent of IBD in humans) (Guan, 2019).

### Casp8^ECko^ mice present small intestinal vascular dysfunction

**Figure EV3:**
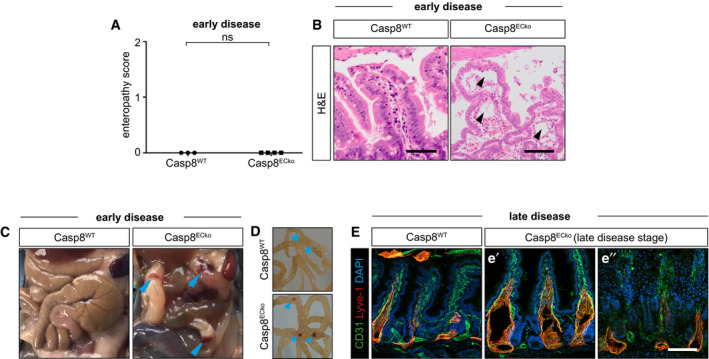
Casp8^ECko^ mice present increasing vascular disintegration, including edema and loss of tissue architecture in the course of disease progression AQuantification of intestinal pathology at an early disease stage (enteropathy score; *n* = 3 WT, 4 ECko, two‐tailed unpaired student *t*‐test).BRepresentative pictures of the small intestinal villi of Casp8^WT^ and Casp8^ECko^ mice at an early disease stage (black arrow heads point to edema in Casp8^ECko^ mice). Scale bars: 100 µm.C, DRepresentative pictures of the small intestine of Casp8^WT^ and Casp8^ECko^ mice at an early disease stage (blue arrow heads point to Peyer’s Patches in Casp8^ECko^ mice). Cleaned intestines are shown in D for better visualization of Peyer’s Patches (blue arrowheads).ERepresentative images of stainings for CD31, Lyve1, and DAPI in small intestinal sections of Casp8^WT^ and Casp8^ECko^ mice at a late disease stage. Lacteals in Casp8^ECko^ mice are severely dilated in villi that are still present (e′). Most of the villi are blunted, showing severe tissue disintegrity (e″). Scale bars: 100 µm. Data information: All data is shown as mean ± SEM. ns: not significant. Source data are available online for this figure.

**Figure 3:**
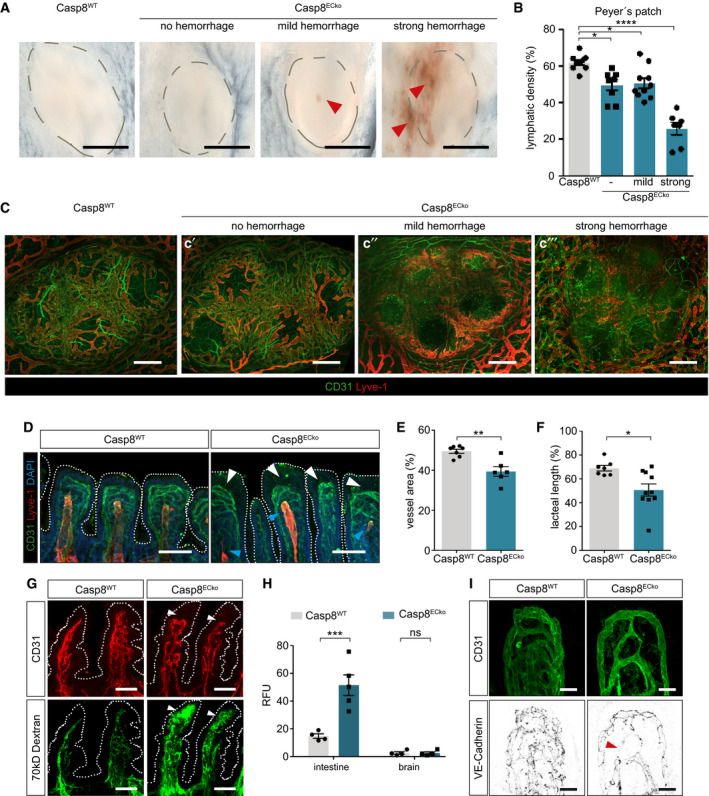
Dysfunction of the lymphatic vasculature and GVB breakdown precede disease manifestation in Casp8^ECko^ mice ARepresentative pictures showing Peyer’s Patches in Casp8^WT^ mice, and different severities of hemorrhage formation in Casp8^ECko^ mice at an early disease stage. Scale bars: 1 mm.B, CQuantification of lymphatic vessel density (B) and representative pictures of CD31 (pan EC) and Lyve1 (lymphatic) staining (C) in Peyer’s Patches in Casp8^WT^ and Casp8^ECko^ mice at an early disease stage (*n* = 9 WT, 7–10 ECko Peyer’s Patches are pooled from 4–6 mice per genotype; one‐way ANOVA with Tukey’s multiple comparison). Scale bars: 200 µm.D–FRepresentative images (D) and quantifications (E, F) of stainings for CD31, Lyve1, and DAPI (nuclei) in small intestinal wholemounts at an early disease stage (blue arrowheads point to defective lacteals, white arrowheads indicate edema) in Casp8^ECko^ mice (E; *n* = 7 WT, 6 ECko. F; *n* = 7 WT, 10 ECko; two‐tailed unpaired Student’s *t*‐test). Scale bars: 100 µm.GRepresentative images of small intestine sections of mice injected with a 70 kDa fluorescently labeled dextran and stained for CD31 at an early disease stage. White arrowheads point to extravasated dextran in Casp8^ECko^ mice (*n* = 5 WT, 5 ECko). Scale bars: 50 µm.HQuantification of extravasated dextran in small intestine and brain lysates in Casp8^WT^ and Casp8^ECko^ mice at an early disease stage (*n* = 4 WT, 4–5 ECko; two‐way ANOVA with Tukey’s multiple comparison). RFU: Relative Fluorescent Units.IRepresentative images of CD31 and VE‐Cadherin staining in intestinal wholemounts at an early disease stage (*n* = 5 WT, 5 ECko) in Casp8^WT^ and Casp8^ECko^ mice (red arrowheads point to VE‐Cadherin “empty” vessel patches). Scale bars: 25 µm. Data information: Data shown as mean ± SEM. *P < 0.05, **P < 0.01, ***P < 0.001, ****P < 0.0001, ns: not significant. Source data are available online for this figure.

To mechanistically understand how the loss of Casp8 in ECs resulted in hemorrhages and loss of intestine homeostasis, we characterized the intestinal vasculature of Casp8^ECko^ mice at different stages of disease progression. We focused on an early disease stage when mice presented no overt inflammatory pathology (Fig EV3A). At an early disease stage, hemorrhages were predominantly present in Peyer’s patches (Fig EV3C and D), independent of their position along the proximal‐distal axis (Esterhazy *et al*, 2019). We therefore analyzed vessel morphology specifically in Peyer’s Patches and distinguished between non‐hemorrhagic, mildly hemorrhagic, and severely hemorrhagic ones (Fig 3A) to study the order of events in more detail. Peyer’s Patches are extensively vascularized by efferent lymphatic vessels that drain lymph and immune cells to the mesenteric lymph nodes (Pellas & Weiss, 1990). Even before hemorrhage formation, lymphatic vessel density was already significantly reduced (Fig 3B and C, and c′), whereas blood vessels still seemed to be unaffected, indicating that changes in the lymphatic vasculature are an early event in disease development. In mildly, and especially strongly hemorrhagic Peyer’s Patches at an early disease stage, lymphatic vessel density was even further reduced, and blood vessels were also severely impaired (Fig 3B and C, c″, c‴). Next, we analyzed the vasculature in small intestinal villi, where edema was observed (Fig EV3B). Vessel density, as well as lacteal length, were significantly reduced in Casp8^ECko^ mice (Fig 3D–F), indicating that also here, both the blood and lymphatic vasculature were affected. To analyze blood vessel integrity, we performed functional assays. Analysis of extravasation of a 70 kDa fluorescently labeled tracer that can normally not cross the GVB (Spadoni *et al*, 2015) showed increased vessel permeability in the intestine in Casp8^ECko^ mice (Fig 3G and H). Consistent with the gut‐specific organotypic effects of Casp8 deletion in ECs, vessel permeability in the brain was not impaired in Casp8^ECko^ (Fig 3H). Increased intestinal vessel permeability was accompanied by the appearance of vessels without junctional VE‐Cadherin staining (Fig 3I). At later disease stages, vessel architecture in the villi was severely impaired, and lacteals strongly dilated (Fig EV3E). Taken together, loss of Casp8 in the endothelium results in intestinal vascular dysfunction in both the lymphatic and blood vasculature.

### Vascular dysfunction is rescued, and intestine homeostasis restored in Casp8^ECko^/ MLKL^ko^ mice

**Figure 4:**
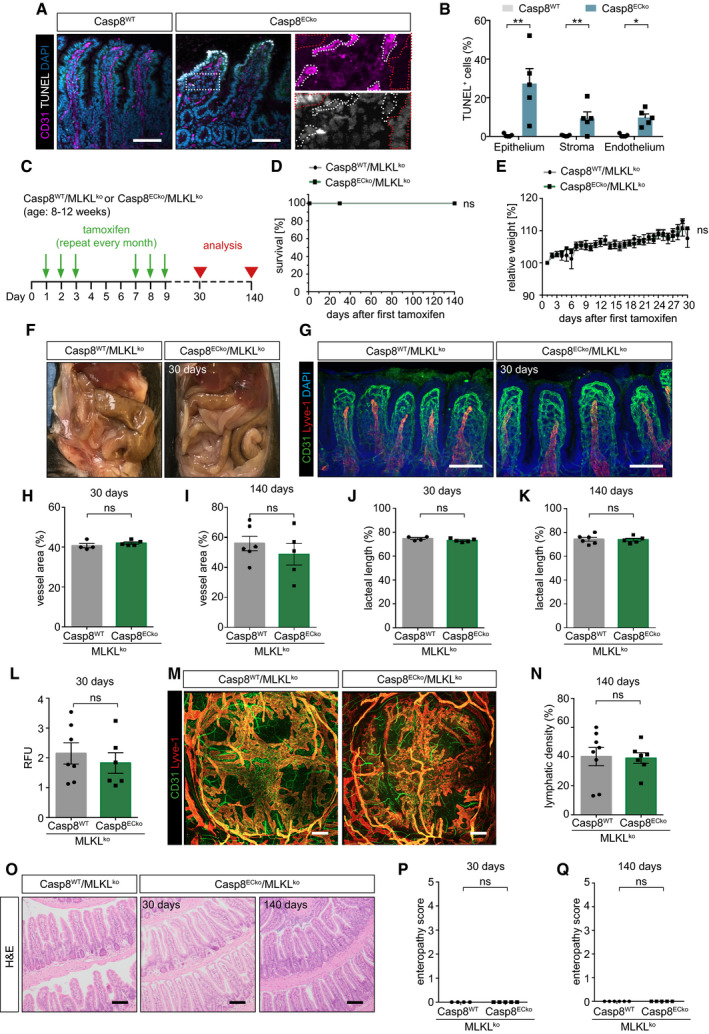
Vascular dysfunction is rescued, and intestine homeostasis restored in Casp8^ECko^/ MLKL^ko^ mice A, BRepresentative images (A) and quantification (B) of CD31, TUNEL, and DAPI staining in small intestine sections of Casp8^WT^ and Casp8^ECko^ mice at an early disease stage (red dotted lines: epithelium; white dotted lines: endothelium) in (*n* = 5 WT, 5 ECko, multiple *t*‐tests with Holm–Sidak method). Scale bars: 100 µm.CSchematic representation of the strategy of tamoxifen treatment and timepoint of analysis in Casp8^WT^/MLKL^ko^ or Casp8^ECko^/MLKL^ko^ mice.D, EGraphs showing survival and relative weight of Casp8^WT^/MLKL^ko^ and Casp8^ECko^/MLKL^ko^ mice upon tamoxifen treatment. (D; *n* = 5‐8 WT/ MLKLko, 5‐8 ECko/MLKLko; Log‐Rank test, E; *n* = 7 WT, 8 ECko; two‐tailed unpaired Student’s *t*‐test).FRepresentative pictures of intestines of Casp8^WT^/MLKL^ko^ and Casp8^ECko^/MLKL^ko^ mice at 30 days after tamoxifen treatment (*n* = 8 WT, 8 ECko).GRepresentative images of stainings for CD31, Lyve1, and DAPI in small intestinal wholemounts from Casp8^WT^/MLKL^ko^ and Casp8^ECko^/MLKL^ko^ mice at 30 days after tamoxifen treatment. Scale bars: 100 µm.H–KQuantification of vessel area (H, I) and lacteal length (J, K) at two different timepoints after tamoxifen treatment in Casp8^WT^/MLKL^ko^ and Casp8^ECko^/MLKL^ko^ mice (*n* = 4–6 WT/MLKLko, 5 ECko/MLKLko; two‐tailed unpaired Student’s *t*‐test).LGraph showing quantification of extravasated 70 kDa Dextran from small intestine tissue of Casp8^WT^/MLKL^ko^ and Casp8^ECko^/MLKL^ko^ mice at 30 days after tamoxifen treatment (*n* = 7 WT/MLKLko, 6 ECko/MLKLko; two‐tailed unpaired Student’s *t*‐test).M, NRepresentative pictures (M) and quantification (N) of lymphatic vessel density in Peyer’s Patches of Casp8^WT^/MLKL^ko^ and Casp8^ECko^/MLKL^ko^ mice at 140 days after tamoxifen treatment (*n* = 8 WT, 7 ECko Peyer’s Patches are pooled from 4 mice per genotype; two‐tailed unpaired students *t*‐test). Scale bars: 200 µm.ORepresentative pictures of H&E stainings of small intestine tissue of Casp8^WT^/MLKL^ko^ and Casp8^ECko^/MLKL^ko^ mice at two different timepoints after tamoxifen treatment (*n* = 4–6 WT, 4–5 ECko). Scale bars: 100 µm.P, QQuantification of intestinal pathology in Casp8^WT^/MLKL^ko^ and Casp8^ECko^/MLKL^ko^ mice at two different timepoints after tamoxifen treatment (enteropathy score; *n* = 4–6 WT/MLKLko, 5 ECko/MLKLko; two‐tailed unpaired Student’s *t*‐test). Data information: All data is shown as mean ± SEM. ns: not significant. Source data are available online for this figure.

As Casp8 is a pro‐survival factor that blocks necroptosis (Weinlich *et al*, 2013), we next analyzed cell death as a potential cause of vascular barrier disruption in Casp8^ECko^ mice. TUNEL^+^ CD31^+^ ECs were already observed at an early stage (Fig 4A and B). Interestingly, TUNEL^+^ cells were also observed in the epithelium and stromal (non‐endothelial, non‐epithelial) compartment (Fig 4A and B), highlighting the requirement of a proper GVB as a crucial safeguard of gut homeostasis. The amount of cell death in all cellular compartments was increased even further at later disease stages (Appendix Fig S2A and B). MLKL is the key executioner molecule of necroptosis (Gong *et al*, 2017). To genetically inhibit this cell death pathway, we crossed Casp8^ECko^ with MLKL^ko^ mice (Casp8^ECko^/MLKL^ko^). Adult Casp8^ECko^/MLKL^ko^ were healthy and viable throughout the whole observation time, when Casp8^ECko^ mice had already succumbed (Fig 4C–E). Neither at 30 nor at 140 days after tamoxifen treatment, Casp8^ECko^/MLKL^ko^ mice presented intestinal hemorrhages, changes in vessel density, lacteal length, and vessel permeability (Fig 4F–L). Lymphatic density in Peyer’s Patches was also completely restored in Casp8^ECko^/MLKL^ko^ (Fig 4M and N). Furthermore, Casp8^ECko^/MLKL^ko^ mice did not develop histopathological signs of small bowel inflammation (Fig 4O–Q). Taken together, these results suggested that EC necroptosis was the primary reason for increased vessel permeability and intestinal vascular dysfunction in Casp8^ECko^ mice. Our data also demonstrates the cooperative nature of the two gut barriers, as vascular dysfunction leads to breakdown of the gut epithelium.

### Lymphatics are the primary driver of vascular dysfunction and small bowel inflammation in Casp8^ECko^ mice

**Figure 5:**
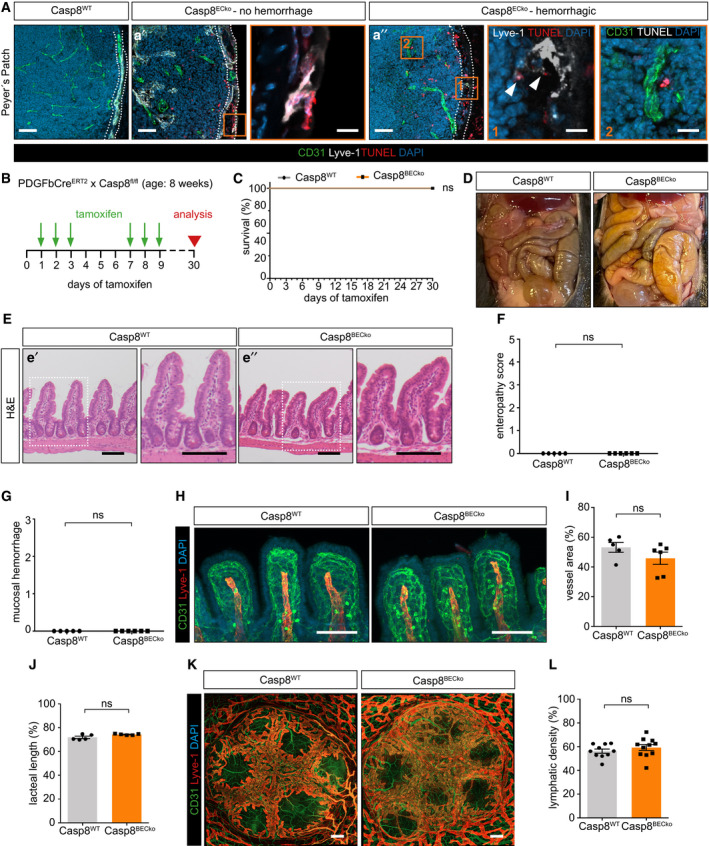
Lymphatic ECs are the primary driver of disease in Casp8^ECko^ mice ARepresentative pictures of TUNEL staining together with CD31 and Lyve‐1 in Peyer’s Patches of Casp8^WT^ and Casp8^ECko^ mice. TUNEL^+^ LECs, but not BECs, could already be seen in non‐hemorrhagic Peyer’s Patches (a′), and further accumulated in hemorrhagic Peyer’s Patches (a″). White dotted areas outline location of the lymphatic plexus. White arrowheads point to TUNEL^+^ LECs (note that necroptotic LECs only weakly express Lyve‐1). Scale bars: 50 µm, insets: 10 µm.BSchematic representation of Casp8 deletion in PDGFb‐Cre^ERT2^ × Casp8^fl/fl^ mice (Casp8^BECko^) indicating the time points for tamoxifen treatment and analysis.CSurvival (*n* = 5 WT, 6 BECko; Log‐Rank test) of Casp8^WT^ and Casp8^ECko^ mice upon tamoxifen treatment.DRepresentative pictures of intestines of Casp8^WT^ and Casp8^BECko^ mice.E–GRepresentative pictures of H&E staining of small intestine of Casp8^WT^ and Casp8^BECko^ mice (E), enteropathy score (F; *n* = 5 WT, 6 ECko, two‐tailed unpaired Student’s *t*‐test), and mucosal hemorrhages (G; *n* = 5 WT, 6 ECko, two‐tailed unpaired Student’s *t*‐test). Scale bars: 100 µm, insets: 50 µm.H–JRepresentative images of stainings for CD31, Lyve1, and DAPI in small intestinal wholemounts (H) and quantification of vessel density (I) and lacteal length (J) in Casp8^BECko^ compared to Casp8^WT^ mice (I; *n* = 5 WT, 6 BECko; two‐tailed unpaired Student’s *t*‐test, J; *n* = 5 WT, 5 BECko; two‐tailed unpaired Student’s *t*‐test with Welch’s correction). Scale bars: 100 µm.K, LRepresentative pictures (K) and quantification (L) of lymphatic vessel density Peyer’s Patches in Casp8^WT^ and Casp8^BECko^ mice (*n* = 10 WT, 11 BECko Peyer’s Patches from 5–6 mice per genotype; two‐tailed unpaired Student’s *t*‐test). Scale bars: 200 µm. Data information: Data shown as mean ± SEM. ns: not significant. Source data are available online for this figure.

As both blood and lymphatic vessels were affected in Casp8^ECko^ mice (Appendix Fig S1F and G, see above), we next studied the behavior of each EC type upon Casp8 deletion in more detail. First, we looked for cell death in the blood versus lymphatic vasculature *in vivo*. We focused our analysis on Peyer’s Patches. Already in non‐hemorrhagic Peyer’s Patches, we detected TUNEL^+^ ECs, exclusively in the lymphatic vasculature (Fig 5A, a′). This was even more prominent in hemorrhagic Peyer’s Patches (Fig 5A, a″) and thus correlated well with the reduced lymphatic density which preceded hemorrhage formation (see Fig 3E and F). Of note, and already in non‐hemorrhagic Peyer’s Patches, we also observed TUNEL^+^ cells in the surrounding tissue (Fig 5A) indicating that, similar to the lamina (Fig 4B and C), vascular dysfunction led to cell death in the adjacent tissue. To further explore the need of Casp8 in lymphatic and blood ECs, we crossed the PDGFb‐CreERT2 mouse line (shown to specifically recombine in blood ECs (BECs) in the intestine (Bernier‐Latmani *et al*, 2015); here also confirmed by GFP expression in CD31^+^/Lyve‐1^‐^ cells (Appendix Fig S3A)) with Casp8^fl/fl^ mice. We induced recombination inf PDGFb‐Cre^ERT2^x Casp8^fl^^/^^fl^ mice (from hereon Casp8^BECko^) using the same tamoxifen treatment protocol as indicated in Fig 5B, which led to significant downregulation of Casp8 expression (Appendix Fig S3B). Notably, Casp8^BECko^ mice were viable (Fig 5C) and did not show visible hemorrhages (Fig 5D). Furthermore, we did not observe histopathological signs of small bowel inflammation (Fig 5E and F), mucosal hemorrhages (Fig 5G), changes in vessel density (Fig 5H and I) or lacteal length (Fig 5H and J). Lymphatic density in Peyer’s Patches (Fig 5K and L) was not different between Casp8^BECko^ mice and Casp8^WT^ mice. Taken together, our data shows that the sole deletion of Casp8 in BECs is not sufficient to recapitulate the phenotype observed in (pan EC) Casp8^ECko^ mice. It also suggests that Casp8 expression in the lymphatic vasculature is a primary requirement for vessel and thus organ homeostasis in the small intestine.

### Inflammation alone is not sufficient to induce vascular defects and pathology in other organs in Casp8^ECko^ mice

**Figure EV4:**
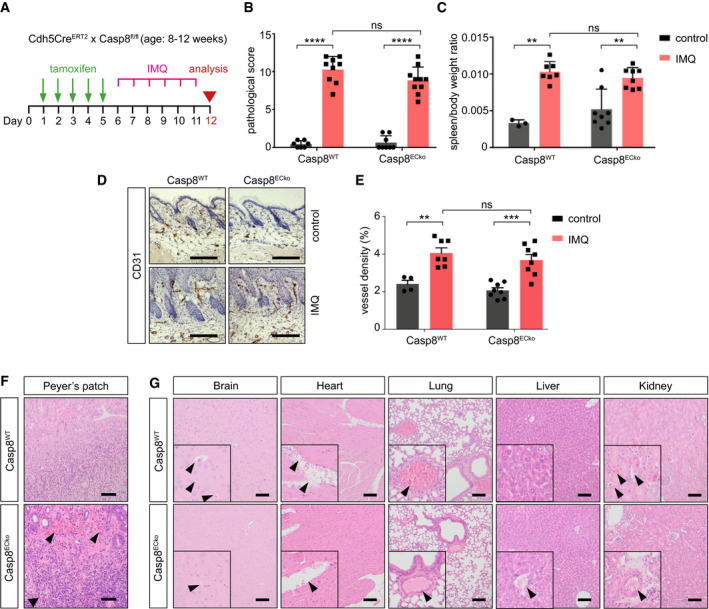
Inflammation alone is not sufficient to induce vascular defects and pathology in other organs in Casp8^ECko^ mice ASchematic representation of imiquimod (IMQ) treatment on the back skin of Casp8^WT^ and Casp8^ECko^ mice.B, CQuantification of skin pathology (B) and spleen weight (C) in Casp8^WT^ and Casp8^ECko^ mice with and without IMQ treatment (*n* = 4–7 WT control, 7–9 WT IMQ, 8 ECko control, 8–10 ECko IMQ; two‐way ANOVA with Sidak’s multiple comparison).DRepresentative pictures of CD31 and hematoxylin staining on skin cross sections of Casp8^WT^ and Casp8^ECko^ mice with and without IMQ treatment. Scale bars: 100 µm.EGraph showing quantification of CD31^+^ vessel area upon IMQ treatment in Casp8^WT^ and Casp8^ECko^ mice (*n* = 4 WT control, 7 WT IMQ, 8 ECko control, 8 ECko IMQ; two‐way ANOVA with Sidak’s multiple comparison).FRepresentative images of H&E staining of Peyer’s Patches from TNF‐α treated Casp8^WT^ and Casp8^ECko^ mice. Black arrowheads point to hemorrhages (*n* = 5 WT, 5 ECko). Scale bars: 50 µm.GRepresentative images of H&E staining of the indicated organs in Casp8^WT^ and Casp8^ECko^ mice 24 h after TNF‐α injection (*n* = 5 WT, 5 ECko). Black arrowheads point to erythrocytes inside of (healthy) blood vessels. Scale bars: 100 µm. Data information: All data is shown as mean ± SEM. **P < 0.01 ***P < 0.001, ****P < 0.0001; ns: not significant. Source data are available online for this figure.

Our data indicates that Casp8 is specifically required in intestinal ECs under physiological conditions to maintain gut homeostasis. As we observed a similar recombination efficiency in ECs of different organs (see Appendix Fig S1), and as Casp8 and other molecules of the necroptosis signaling pathway (such as *Ripk3*, *Tnfr1*, and *Mlkl)* are similarly expressed in ECs of different organs (Kalucka *et al*, 2020); Appendix Fig S4), we considered that other environmental factors would need to be involved in the manifestation of the intestinal phenotype. To better understand the organ specificity of the phenotype, we questioned whether the observed inflammation would be required and sufficient to induce vascular hemorrhages. If so, we reasoned that challenging other organs with an inflammatory insult would also lead to vascular defects in those organs in Casp8^ECko^ mice. First, we used a psoriasis inflammation model by local application of the immune activator imiquimod (IMQ) to challenge the skin—another epithelial barrier tissue in the body (Fig EV4A). Both Casp8^WT^ and Casp8^ECko^ mice developed equally strong skin inflammation and epidermal hyperplasia (Fig EV4B), as well as increased spleen weight (as measure of inflammation, Fig EV4C). No hemorrhages were observed, and analysis of blood vessel density showed equal inflammation‐induced angiogenesis after IMQ treatment in the skin in both genotypes (Fig EV4D and E). Thus, loss of Casp8 in ECs does not generally predispose mice to the increased disease development or hemorrhage formation in other organs—such as in this case in the skin—in the presence of inflammatory insults.

**Figure 6:**
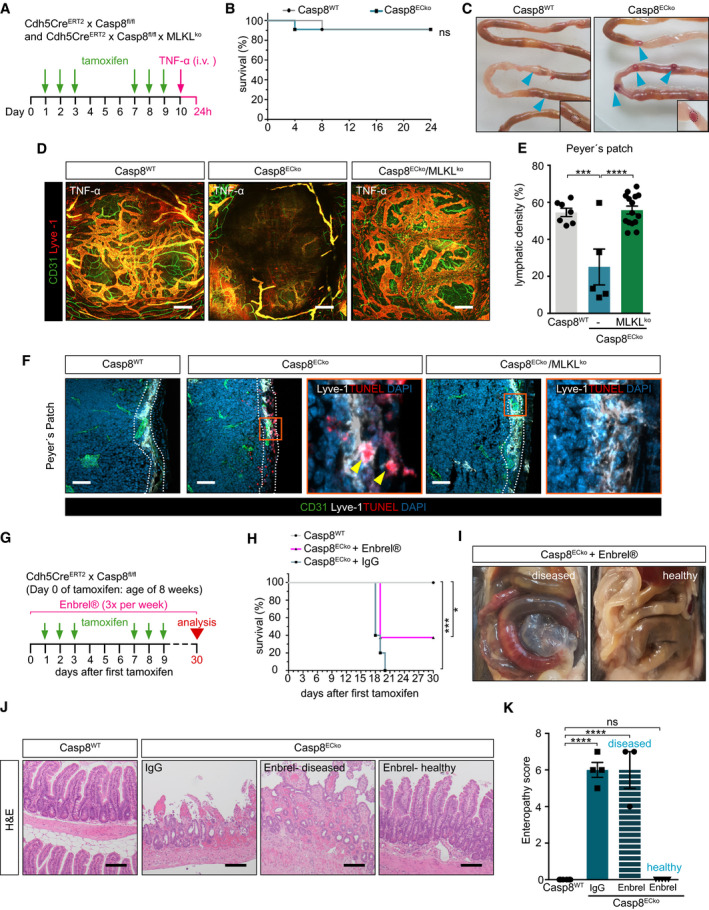
Intravenous TNF‐α injection in Casp8^ECko^ mice causes specific bleedings in the Peyer’s patches but in no other organ ASchematic representation of the strategy to induce systemic inflammatory response syndrome (SIRS) via intravenous TNF‐α injection.BSurvival graph after intravenous TNF‐α injection of Casp8^WT^ versus Casp8^ECko^ mice within the first 24 h (*n* = 11 WT, 11 ECko; Log‐Rank test).CRepresentative images of the small intestines 24 h after TNF‐α injection in Casp8^WT^ and Casp8^ECko^ mice (blue arrowheads point to Peyer’s Patches).D, ERepresentative pictures of Peyer’s Patches (D) and quantification of lymphatic vessel density (E) in Casp8^WT^, Casp8^ECko^, and Casp8^ECko^/MLKL^ko^ mice injected with TNF‐α (*n* = 7 WT, 5 ECko, 15 ECko/MLKLko. Peyer’s Patches are pooled from 3–5 mice per genotype). Scale bars: 200 µm.FRepresentative pictures of TUNEL staining together with CD31 and Lyve‐1 in Peyer’s Patches of Casp8^WT^, Casp8^ECko^, and Casp8^ECko^/MLKL^ko^ mice injected with TNF‐α. TUNEL^+^ LECs, but not BECs, could already be seen in Casp8^ECko^, but neither in Casp8^WT^ nor in Casp8^ECko^/MLKL^ko^ mice. White dotted areas outline location of the lymphatic plexus. Yellow arrowheads point to TUNEL^+^ECs). Scale bars: 50 µm.GSchematic representation of the Enbrel^®^ treatment protocol to inhibit TNF‐α signaling.HSurvival graph of Casp8^WT^ and Casp8^ECko^ mice after Enbrel^®^ treatment (*n* = 7 WT, 5 ECko^+^ IgG, 8 ECko^+^ Enbrel; Log‐Rank test).I–KRepresentative images of small intestines (I) and H&E staining (J) with histopathological scoring (K) of Casp8^WT^ and Casp8^ECko^ mice after Enbrel^®^ treatment (*n* = 6 WT, 4 ECko^+^ IgG; 8 ECko^+^ Enbrel (from which 3 mice were diseased, 5 mice healthy); one‐way ANOVA with Tukey’s multiple comparison). Scale bars: 100 µm. Data information: All data is shown as mean ± SEM. *P < 0.05, ***P < 0.001, ****P < 0.0001; ns: not significant. Source data are available online for this figure.

Next, we used a model of systemic inflammatory response syndrome (SIRS) to provoke a systemic inflammatory reaction (Matsuda & Hattori, 2006) and thus to systemically target the vasculature. For this, we intravenously injected a moderate dose of TNF‐α that is not lethal to wildtype mice (Gunther *et al*, 2011) at a time point when no vascular defects or hemorrhages were present in Casp8^ECko^ mice (10 days after first tamoxifen treatment, Fig 6A). As reported, this moderate dose of TNF‐α did not result in increased lethality (Fig 6B). Twenty‐four hours after TNF‐α injection, we saw specific hemorrhages in the Peyer’s Patches in Casp8^ECko^ mice (Figs 6C and EV4F), but in no other organ. At least on a histological level (no other parameters were analyzed), and within the timeframe of observation, only the small intestine was affected (Fig EV4G). In this model, vessel analysis on Peyer’s Patch (wholemounts) revealed similar defects as seen after spontaneous disease development in Casp8^ECko^ mice, with regressing lymphatic vessels and disrupted blood vessels (Fig 6D and E). This phenotype was completely prevented in Casp8^ECko^/MLKL^ko^ mice (Fig 6D and E) that were injected with TNF‐α. Again, TUNEL^+^ ECs were exclusively found in Lyve1^+^, lymphatic vessels of Casp8^ECko^ mice, but not Casp8^ECko^/MLKL^ko^ mice (Fig 6F), suggesting that lymphatic demise preceded demise of the blood vasculature. This result further suggests that the observed intestinal phenotype occurs in particular in this organ due to the presence of organ‐specific components.

As TNF‐α is an important necroptosis inducing cytokine, TNF‐α was elevated in the intestine of Casp8^ECko^ mice (see Fig 2A), and as anti‐TNF‐α treatment is used in IBD patients (Peyrin‐Biroulet *et al*, 2021), we questioned whether the observed phenotype in Casp8^ECko^ could be due to TNF‐α signaling. For this, we treated Casp8^ECko^ mice with the TNF‐α inhibitor Enbrel^®^ three times per week (Fig 6G). Only in a subset of mice (40% of Casp8^ECko^ mice), TNF‐α inhibition fully prevented lethality and disease development (Fig 6H–K) as shown by histopathological analysis, whereas as 60% of the Casp8^ECko^ mice still developed disease similar to Casp8^ECko^ mice treated with IgG control.

Taken together, these data suggested that apart from TNF‐α, another, gut‐specific environmental factor is involved in the appearance of intestinal hemorrhages and indicated that the intestinal vasculature is the primary vascular site requiring Casp8 expression for the maintenance of tissue homeostasis. It further highlights the potential involvement of the Peyer’s Patches as an important structure of the disease development.

### The presence of microbiota is required to induce small bowel inflammation in Casp8^ECko^ mice

**Figure EV5:**
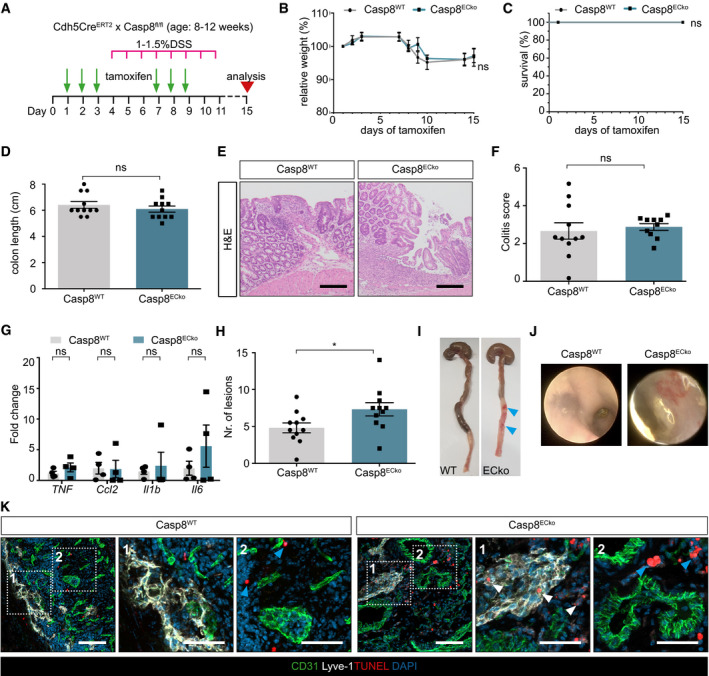
Casp8^ECko^ mice are more susceptible to colitis development upon DSS treatment ASchematic representation of low dosage DSS treatment via the drinking water in Casp8^WT^ and Casp8^ECko^ mice.B, CGraphs showing weight loss (B) and lethality (C) in Casp8^WT^ and Casp8^ECko^ mice (*n* = 11 WT, 11 ECko; B: curve comparison; C: Log‐Rank test).DColon length of Casp8^WT^ and Casp8^ECko^ mice after tamoxifen treatment (*n* = 11 WT, 11 ECko; two‐tailed unpaired Student’s *t*‐test).E, FRepresentative images (E) and histopathological evaluation (F) of H&E staining of the large intestine of Casp8^WT^ and Casp8^ECko^ mice (*n* = 11 WT, 10 ECKo; unpaired *t*‐test with Welch’s correction). Scale bars: 100 µm.GQPCR analysis of pro‐inflammatory cytokines in Casp8^WT^ and Casp8^ECko^ mice upon DSS treatment (*n* = 4 WT, 4 ECko; multiple *t*‐tests with Holm–Sidak method).HQuantification of the total number of inflammatory lesions per section in the colon of Casp8^ECko^ compared to Casp8^WT^ mice upon DSS treatment (*n* = 11WT, 11ECko, unpaired Student’s *t*‐test).I, JRepresentative pictures of large intestines (I) and colon endoscopy (J) of Casp8^WT^ mice and Casp8^ECko^ mice after DSS treatment. Blue arrowheads point to hemorrhages.KRepresentative pictures of TUNEL staining together with CD31 and Lyve‐1 staining shows TUNEL^+^ LECs, not BECs in Casp8^ECko^, but not Casp8^WT^ mice upon DSS treatment. Blue arrow heads point TUNEL^+^ cells outside the vessels. White arrow heads point to TUNEL^+^ cells inside Lyve‐1^+^ lymphatics. Scale bars: 50 µm, insets 25 µm. Data information: Data shown as mean ± SEM. *P < 0.05; ns: not significant. Source data are available online for this figure.

The intestinal microbiota is a crucial environmental feature that distinguishes the small intestine from other vascular niches in other organs. It has been shown that microbiota stimulates TNF‐α production in myeloid cells in the intestine, and that this is sufficient to cause EC apoptosis in the absence of anti‐apoptotic molecules such as TAK 1 (Naito *et al*, 2019), suggesting the existence of a microbiota‐immune cells‐endothelium signaling hub. However, as intestinal ECs themselves express the main Toll‐like receptors (TLRs) at a low level (Appendix Fig S5), it might also be that microbial products are additionally directly detected by ECs. As hemorrhages first occurred in Peyer’s Patches, and Peyer’s Patches hold the biggest immune cell repository and participate substantially in the sampling of bacteria and bacterial products (Jung *et al*, 2010), we speculated that microbiota could participate in the development of the phenotype by stimulating a pro‐inflammatory environment in Peyer’s Patches. To test this hypothesis, we took advantage of the fact that Casp8^ECko^ mice do not show a phenotype in the large intestine (Fig EV1B–D). Therefore, we challenged Casp8^ECko^ mice with a Dextran sodium sulfate (DSS) treatment that leads to colonic inflammation that indirectly depends on translocation of bacteria upon chemical disruption of the epithelial barrier (Eichele & Kharbanda, 2017). Upon treatment with a low dose of DSS (Fig EV5A), both Casp8^WT^ and Casp8^ECko^ mice developed mild ulcers with similarly severe depth and inflammation (Fig EV5B–G). However, Casp8^ECko^ mice presented an increased number of lesions (Fig EV5H; Movie EV1 and Movie EV2) and macroscopic hemorrhages compared to Casp8^WT^ mice (Fig EV5I and J), indicating that Casp8^ECko^ mice are more susceptible to DSS‐induced colitis. Of note, we observed TUNEL^+^ LECs, but not BECs, in Casp8^ECko^ mice compared to Casp8^WT^ controls upon DSS treatment (Fig EV5K), highlighting the contribution of LEC death in this model.

**Figure 7:**
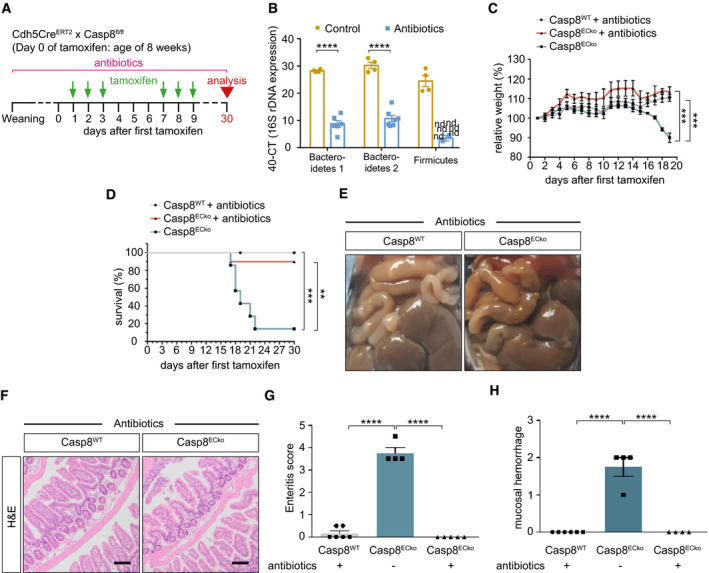
The presence of microbiota is required to induce small bowel inflammation in Casp8^ECko^ mice A, BSchematic representation of the strategy for antibiotic treatment of Casp8^WT^ and Casp8^ECko^ mice (A) and analysis of the most prominent bacterial phyla (B; *n* = 4 Control, 6–8 Antibiotics; multiple *t*‐tests with Holm–Sidak method) by qPCR analysis. In B, the cycle number of 16S rDNA expression compared to the total number of cycles is shown.C, DGraphs showing the relative weight (C; *n* = 11 WT, 7 ECko, 10 ECko^+^ antibiotics one‐way ANOVA with Tukey’s multiple comparison) and the survival of mice (D; *n* = 6 WT, 7 ECko, 7 ECko^+^ antibiotics; Log‐Rank test) upon tamoxifen treatment in the presence of an antibiotic cocktail.ERepresentative images of the gut of Casp8^WT^ and Casp8^ECko^ mice with antibiotic treatment 30 days after tamoxifen.F–HRepresentative images of H&E staining (F). Quantification of intestinal pathology (G) and mucosal hemorrhages (H) in Casp8^WT^ and Casp8^ECko^ mice treated with antibiotics compared to untreated Casp8^ECko^ mice (G; *n* = 6 WT, 4 ECko^+^ antibiotics, 5 ECko; one‐way ANOVA with Tukey’s multiple comparison, H; *n* = 6 WT, 4 ECko^+^ antibiotics, 4 ECko; one‐way ANOVA with Tukey’s multiple comparison). Data information: Data shown as mean ± SEM. **P < 0.01, ***P < 0.001, ****P < 0.0001; ns: not significant. Scale bars: 100 μm. Source data are available online for this figure.

Next, we depleted the intestinal microbiota by adding a cocktail of antibiotics to the drinking water of Casp8^ECko^ and Casp8^WT^ mice after weaning (Fig 7A), which resulted in a strong reduction of intestinal microbiota (Fig 7B). Of note, the intestinal microbiome between Casp8^ECko^ and control littermates did not differ significantly at early disease stages, as indicated by diversity analysis after 16S ribosomal (r) RNA gene sequencing (Appendix Fig S6A and B), suggesting that initial differences in the intestinal microbiome are not the trigger of disease development. Casp8^ECko^ mice treated with antibiotics remained healthy at least until 30 days after the first tamoxifen treatment, while the majority of Casp8^ECko^ mice receiving normal drinking water had already died (Fig 7C and D). In line, histological evaluation neither showed histopathological differences between Casp8^WT^ and Casp8^ECko^ mice receiving antibiotics (Fig 7E–G) nor hemorrhages (Fig 7H). Taken together, this data shows that the presence of intestinal microbiota is required to trigger disease development in Casp8^ECko^ mice in a gut‐specific manner.

## Discussion

Sustaining EC homeostasis is an active process in the adult organism, and active signaling input is required to maintain vascular integrity (Dejana *et al*, 2017). Both blood and lymphatic ECs reside in specialized vascular niches that determine their organotypic features (Nolan *et al*, 2013; Hagerling *et al*, 2018; Hong *et al*, 2020). The presence of microbiota in the gastrointestinal tract is a challenge that requires constant immune surveillance and cytokine production to maintain tolerance. Concurrently, several strategies and compartmentalized structural and immunological barriers, such as the gut‐epithelial and ‐vascular barrier and the gut lymphatic vascular system, have evolved to protect the host from the threat of infection. Here we identify Casp8 as a molecular factor in adult ECs that is required for maintaining gut‐vascular homeostasis in a tissue‐specific manner. We also show that vascular dysfunction is not just a consequence but can also be a primary driver of chronic intestinal inflammation.

Increased cell death in intestinal epithelial cells has been shown to promote IBD development in humans (Gunther *et al*, 2011; Pierdomenico *et al*, 2014; Lehle *et al*, 2019) as well as chronic intestinal inflammation in different mouse models (Gunther *et al*, 2011; Welz *et al*, 2011; Wittkopf *et al*, 2013; Vlantis *et al*, 2016; Schwarzer *et al*, 2020). All of these studies solely focused on intestinal epithelial cells and identified multiple pro‐survival and cell death regulatory proteins, such as Casp8, that are key for maintaining epithelial cell survival (Gunther *et al*, 2011; Welz *et al*, 2011; Wittkopf *et al*, 2013; Vlantis *et al*, 2016; Schwarzer *et al*, 2020). Here we show that dyshomeostasis of ECs can also promote intestine inflammation, and that the same factor—Casp8—also maintains the GVB and lymphatic vasculature (and thus gut‐epithelial barrier integrity in a non‐cell‐autonomous manner). Indeed, EC‐specific Casp8^ECko^ mice develop a phenotype that manifests specifically in the gut and die within 3 weeks upon acute Casp8 deletion due to massive intestinal hemorrhages and inflammation. We are here providing a first characterization of the inflammatory phenotype by cytokine analysis and immune cell stainings and acknowledge that further in‐depth analysis of the immune cell compartment is a necessary step in the future in order to fully validate our mouse model.

Interestingly, in addition to EC death, we also detected dead cells in the epithelial and stromal compartments, which can be secondary to EC demise and strong inflammation, or potentially due to the loss of proper angiocrine signaling required to maintain tissue homeostasis. It has been reported that LEC depletion in mice leads to rapid lethality due to intestine‐specific hemorrhages and sepsis (Jang *et al*, 2013). Furthermore, exposure of intestinal epithelial cells with lymph fluid in a mouse model of lymphangiectasia leads to increased barrier permeability (Gonzalez‐Loyola *et al*, 2021). Consistently, here we find that already in the initial steps of disease development in Casp8^ECko^ mice, Peyer’s Patches display altered lymphatic vessel structure and LEC death and lacteals in the villi are also affected. Of note, small bowel inflammation and vascular dysfunction do not occur when Casp8 is solely deleted in BECs (Casp8^BECko^ mice). Altogether, this data suggests that the presence of Casp8 in LECs is important for maintaining healthy LECs, and thus important for overall vascular and intestine homeostasis. How exactly the intestinal lymphatic vascular system regulates the blood endothelium in this model requires further investigation.

We furthermore show that disease development depends on the presence of intestinal microbiota, as antibiotic treatment protected Casp8^ECko^ mice from disease development. Peyer’s Patches play an important role in receiving and conveying information about the intestinal microbiota under steady state conditions in the healthy adult mouse (Jung *et al*, 2010; Morikawa *et al*, 2016). The intestinal microbial flora stimulates the development, maintenance, and activity of the intestinal immune system (Zhou *et al*, 2020), such as TNF‐α production by CD11b^+^ myeloid cells (Naito *et al*, 2019), and blockade of TNF‐α is a common treatment in patients with small inflammatory diseases (Bosani *et al*, 2009). While microbial products might also be directly detected by ECs (which requires further investigations), our data supports a model where a microbiota‐induced immune cell‐EC signaling hub is crucial for disease development in the intestine. TNF‐α is a potent driver of necroptosis when Casp8 activity is inhibited (Lin *et al*, 2004; He *et al*, 2009; Gunther *et al*, 2011). In line, intravenous TNF‐α injection accelerated hemorrhage formation in the intestine of Casp8^ECko^ mice. It was however not sufficient to induce hemorrhages in other organs, suggesting that a combination of microbiota and cytokine signaling is required to induce vascular demise in Casp8^ECko^ mice. This is supported by our finding that TNF‐α inhibition only partially protected Casp8^ECko^ mice from bowel inflammation. Villus macrophages are in close contact with lacteals, where they regulate diverse homeostatic functions and for example maintain lacteal integrity by secretion of VEGF‐C (Suh *et al*, 2019). Therefore, intestine‐specific immune cell–lymphatic EC communication might also explain the organotypic differences compared to more immune privileged organs.

Of note, even though the microbial burden is higher in the large compared to small intestine (Agace & McCoy, 2017; Hillman *et al*, 2017), the colon was not affected in our model. This is likely due to a thicker mucosal, and thus physical, barrier that prevents microbiota from directly interacting with the intestine tissue (Agace & McCoy, 2017) which leads to a reduced density of immune cells (Bowcutt *et al*, 2014). This is in line with our hypothesis that not only the microbiota, but a microbiota‐regulated immune cell–EC signaling cascade is required in our model.

A recent study reported an organ‐specific requirement of the TGF‐β associated kinase‐1 (TAK‐1), a key regulator of TNF‐α signaling, in intestinal ECs (Naito *et al*, 2019). In the absence of TAK‐1, vascular integrity in the intestine was impaired due to increased TNF‐α dependent EC apoptosis. Importantly, despite hemorrhage formation in these mice, the authors did not report the development of intestine inflammation, and the overall intestine architecture seemed largely intact. In contrast to apoptosis, necroptosis is a highly inflammatory cell death pathway, as dying cells release their cellular content to the extracellular space in an uncontrolled manner, thus boosting immune cell activation (Pasparakis & Vandenabeele, 2015) which might explain the pathological severity observed in Casp8^ECko^ mice. In addition, recent research has elucidated that necroptosis signaling molecules also regulate inflammation independent of their cell death function. For example, it has also been shown that deletion of Casp8, independent of necroptosis, induces autocrine secretion of pro‐inflammatory cytokines in diverse cell types, including ECs (Feltham *et al*, 2017; Henry & Martin, 2017; Kang *et al*, 2018). This might further impact cells in the environment, thus accelerating inflammation and causing tissue dyshomeostasis. It would therefore be interesting to better characterize the secretome of Casp8 knockout intestinal ECs in the future, in particular as we detected many non‐EC TUNEL^+^ in Casp8^ECko^ mice. In line, and furthermore, MLKL promotes vascular inflammation and expression of leukocyte cell adhesion molecules (Dai *et al*, 2020), thus opening up another future avenue on potential EC‐immune cell regulatory mechanism in this model‐beyond necroptosis.

In summary, our work supports a model where the presence of bacteria is required to initiate disease development upon Casp8 deletion in ECs, which is potentiated by the presence of TNF‐α. Our data shows that dysfunction of the lymphatic vasculature, followed by demise of the GVB, is sufficient to drive chronic intestinal inflammation in mice. It further indicates that microbiota do not just regulate intestinal angiogenesis (Stappenbeck *et al*, 2002; Reinhardt *et al*, 2012), but also interact, directly or indirectly, with the adult vasculature. Thus, our study integrates the intestinal endothelium both lymphatic and blood vessels‐ as a novel important player into the circuit of microbiota‐regulated intestine homeostasis.

## Materials and Methods

### Mice

Mice were maintained at the SPF animal facilities of the “Interfaculty Biomedical Facility” of the Heidelberg University and kept under a 12 h light cycle with regular chow diet (LASQCdiet^®^ Rod16) and water ad libitum. Tamoxifen‐inducible pan EC‐specific Casp8 knockout mice were generated by crossing Casp8^fl/fl^ mice (Beisner *et al*, 2005) with Cdh5(PAC)‐CreERT2 mice (Wang *et al*, 2010) and named Casp8^ECko^. Blood EC‐specific tamoxifen‐inducible Casp8 knockout mice have been generated by crossing Casp8^fl/fl^ mice with Pdgfb‐iCreER (Claxton *et al*, 2008), from here on Casp8^BECko^. MLKL^ko^ mice were kindly provided by M. Pasparakis (Uni. Köln). ROSA^mTmG^ are available from Jackson Labs. Recombination in 8–12‐week‐old adult mice was induced by oral gavage of 200 µl tamoxifen (10 mg/ml diluted in peanut oil) for a total of 6 days (see also Fig 1A). For the Psoriasis model, mice received five consecutive tamoxifen treatments (see Appendix Fig S4A). Activity of the Cre recombinase after tamoxifen treatment was analyzed in Cdh5(PAC)‐CreERT2 mice x ROSA^mTmG^ double fluorescent reporter mice (Muzumdar *et al,* 2007). All animal procedures were conducted in accordance with European, national, and institutional guidelines. Protocols were in accordance with the NIH “Guide for the Care and Use of Laboratory Animals” and approved by local government authorities (Regierungspräsidium Karlsruhe, Germany; references T49/15, T46/16, T36/17, T48/18, T38/19, G125/14, G251/16, G134/18). Mice were housed with a 12 h light cycle receiving regular chow diet and water ad libitum and regularly tested for pathogens according to the FELASA guidelines.

### Antibiotic treatment

After weaning from the mother at ~3 weeks, Casp8^ECko^ mice were treated with a mixture of broad‐spectrum antibiotics (Dannappel *et al*, 2014) via the drinking water until the end of the experiment: Ampicillin 1 g/l (Ratiopharm), Neomycin 1 g/l (Sigma), Meronem 0.5 g/l (Friedrich‐Eberth), Ciprofloxacin 0.5 g/l (Sigma). To quantify the number of intestinal bacteria, fecal samples were collected under sterile conditions at the end of the experiment and bacterial DNA was extracted using the QIAamp DNA Stool Mini Kit (QIAGEN). 16S bacterial rDNA was detected and quantified by qRT–PCR. Primer sequences are provided in Appendix Table S1.

### TNF‐α challenge

Ten days after the first tamoxifen treatment, a moderate dose of recombinant murine TNF‐α (Immunotools, 200 ng/g body weight) was injected into the tail vein of Casp8^ECko^, Casp8^ECko^/MLKL^ko^, and control mice. Survival of the mice was monitored for 24 h. Afterward, organs and small intestinal tissue was collected for further analysis.

### TNF‐α inhibitor treatment

Casp8^ECko^ mice were treated with 0.2 mg per injection with either human IgG1 (InVivo plus, BioXCell) or Enbrel^®^ (Pfizer Pharma) three times per week, starting 1 day before the first tamoxifen treatment, and compared to Casp8^WT^ controls.

### DSS‐induced colitis model and endoscopy

Casp8^ECko^ mice were treated with tamoxifen as before (Fig 1A). Starting at 4 days after the first tamoxifen treatment, mice received 1.5% DSS via the drinking water for a total of 8 days. The water was exchanged every second day. Endoscopy analysis was performed at 15 days after the first tamoxifen treatment right before collection of colon samples as previously described (el Marjou *et al*, 2004).

### Psoriasis skin inflammation model

Casp8^ECko^ mice were treated with tamoxifen for 5 consecutive days. Skin inflammation was then induced by daily application of Aldara™ 5% creme on the shaved back skin (5 days). Afterwards, mice were sacrificed, and the spleen weight was recorded as a measurement of inflammation. The back skin was dissected and fixed in 4% PFA for further tissue processing. Inflammation was histopathologically assessed with the following score: Thickness of epidermis [0‐3], Papillomatosis [0‐3], Parakeratosis [0‐3], Scaling [0‐3], Erythema [0‐3]. The combined score was calculated by adding the score for each parameter.

### Dextran blood vessel permeability assay

To measure vessel permeability, Casp8^ECko^, Casp8^ECko^/MLKL^ko^, and control mice were intravenously injected with 0.5 mg (50 µl) of 70 kDa FITC‐labeled Dextran (Thermo Scientific) at 15 or 30 days after the first tamoxifen treatment, respectively. After circulation of the tracer for 30 min, mice were either sacrificed by cervical dislocation to detect the tracer in tissue cryosections or anesthetized and perfused with PBS to then dissect the gut and brain and to extract extravasated tracer from the tissue. To do the latter, mice were anesthetized and blood was collected in 0.5 M EDTA and centrifuged for 5 min at maximum speed at 4°C to determine the serum Dextran levels. Afterward, mice were perfused with 10 ml PBS. 1–2 cm of the ileum or one brain hemisphere were dissociated in 300 µl PBS and centrifuged for 15 min at 10,000 *g* at 4°C. 100 µl of the blood and tissue supernatants was transferred in duplicates to opaque 96‐well plates and excitation/emission was measured at 495/519 nm. The data is expressed as Relative Fluorescent Units (RFU)/mg tissue/RFU serum.

### Primary EC isolation

Lung, brain, skin, and intestine ECs were isolated from adult Casp8^ECko^ mice at 10 or 15 days after the first tamoxifen treatment (see Appendix Fig S2).

#### Lung and brain ECs

Mice were sacrificed by cervical dislocation, and lungs and brain tissue was dissected and stored in HBSS medium on ice. Lung tissue was digested at 37°C in digestion buffer (3.12 mg/ml Collagenase II (Worthington), 3 mg/ml Collagenase/Dispase (Sigma‐Aldrich), 8 U/ml DNAseI (Worthington) in HBSS) for 30 min and dissociated to single cells using the gentleMACS™ dissociator (Miltenyi Biotec). The cell suspension was filtered through 70 µm filters, and red blood cells (RBCs) were lysed for 5 min in RBC lysis buffer (eBioscience™). Brain tissue was digested at 37°C in digestion buffer (3 mg/ml Collagenase/Dispase (Sigma‐Aldrich), 8 U/ml DNAseI (Worthington) in HBSS) and dissociated to single cells using the gentleMACS™ dissociator (Miltenyi Biotec) following the manufacturer’s protocol. Myelin removal was performed according to the manufacturer’s protocol using Myelin removal beads II (Miltenyi Biotec). Lung and brain CD31^+^ ECs were then isolated using the MACS^®^ Technology (Miltenyi Biotec). To do so, cell suspensions were first incubated on ice with magnetic beads coupled to a CD45 antibody (1:10 in 2%FBS/PBS) for 15 min and separated on LD columns (Miltenyi Biotec). Columns were washed three times with 2%FBS/PBS and the flow through was used to purify CD31^+^ cells by incubating the suspension with magnetic beads coupled to a CD31 antibody (1:10 in 2% FBS/PBS) for 15 min. CD31^+^ cells were selected on MS columns (Miltenyi Biotec) and eluted after three washes with 2% FBS/PBS. RNA was extracted with the RNeasy^®^ Mini Kit (QIAGEN) following the manufacturer’s instructions.

#### Skin ECs

Mice were sacrificed by cervical dislocation. 2 cm^2^ of shaved back skin was dissected, chopped with scissors into small pieces, and digested in 2.2 U/ml Collagenase Dispase (Sigma‐Aldrich) and 5 mg/ml Collagenase IV (Worthington Biochemical) in DMEM for 90 min at 37°C. Afterward, the tissue was dissociated to single cells using the gentleMACS™ dissociator (Miltenyi Biotec) and the “human skin” program, filtered, and subsequently selection of CD31^+^ cells has been performed as described above.

#### Intestinal ECs

Mice were sacrificed by cervical dislocation. One half of the small intestine, and the complete colon, were dissected and cleaned from intestinal content with ice‐cold PBS. The tissue was chopped into big junks and incubate in 20 ml of a 10 mM EDTA solution with agitation at 37°C for 20 min to remove epithelial cells. Afterward, several cycles of vortexing and washing with PBS were performed until the supernatant stayed clear of floating epithelial cells. The remaining tissue was cut into very small pieces and then digested with Collagenase IV (Worthington Biochemical, 3 mg/ml) in 0.2%FBS/PBS containing CaCl_2_ (2 mM) and 200 μg/ml DNase I (Worthington Biochemical) in 8 ml final volume for 20 min at 37°C. Afterward, the tissue was dissociated to single cells using the gentleMACS™ dissociator (Miltenyi Biotec) and the “intestine” program. Digestion was stopped by adding 10 ml DMEM containing 10% FBS, the suspension was filtered and processed for flow cytometry. Stained cells were analyzed, and blood and lymphatic ECs were isolated using BD Aria platform and sorted directly into RNA extraction buffer.

### Intestinal epithelial cell isolation

Epithelial cells from small intestinal tissue were isolated as described (Welz *et al*, 2011). Briefly, epithelial cells were isolated from Casp8^ECko^ mice and controls by consecutively incubating 5 cm of longitudinally opened and cleaned small intestinal tissue in 10 ml PBS with 1 mM DTT for 10 min and 20 ml of prewarmed HBSS with 1.5 mM EDTA for 15 min. All incubations were performed at 37°C on a horizontal shaker (180 rpm). Afterward, the tissue was vortexed for 1–2 min at maximum speed. The supernatant containing intestinal epithelial cells was centrifuged for 10 min at 1,200 rpm and the pellet was immediately resuspended in 1 ml TRIzol™ Reagent (Invitrogen) for RNA extraction.

### cDNA transcription and qRT–PCR

cDNA transcription and qRT–PCR have been performed as described (Tisch *et al*, 2019). DNAseI‐treated RNA was reverse‐transcribed into cDNA using either Maxima Reverse Transcriptase (Thermo Scientific) or SuperScript™Vilo™ (Invitrogen™), depending on the RNA concentration. RNA from sorted intestinal blood and lymphatic ECs was prepared using the PicoPure™ RNA Isolation Kit (Thermo Scientific). mRNA expression levels were assessed by qRT–PCR using Fast SYBR Green Master Mix (Applied Biosystems™). Actin was used as housekeeping control. Primer sequences are provided in Appendix Table S1.

### Proteome profiler and analysis

Casp8^ECko^ mice were sacrificed 18 days after the first tamoxifen treatment. The mouse XL Cytokine Array (R&D systems) was performed on ileal lysates according to the manual. Normalization, statistics, and heatmap were done in R [R Core Team (2021) (URL https://www.R‐project.org/)]. Background spot intensities were subtracted from all other spot values. Repeats were normalized on the reference spot intensities. Significance was measured with *t*‐test without *P*‐value correction. GO term and Pathway groups were generated using the Panther database (Mi *et al*, 2019) and custom R scripts.

### Endotheliomics database analysis

A published EC atlas (Kalucka *et al*, 2020) has been used to analyze expression of necroptosis signaling molecules in ECs across different tissues. The “explore single cell datasets” together with “t‐Distributed Stochastic Neighbor Embedding” functions have been used to visualize gene expression.

### Intestinal wholemount stainings

#### Villi

Intestinal wholemount preparations were performed as previously described (Bernier‐Latmani & Petrova, 2016). In brief, mice were anesthetized (Ketamin 120 mg/kg, Xylazin 16 mg/kg body weight) and perfused with 10 ml PBS, followed by perfusion with 10 ml 4% PFA. The small intestine (ileum) was opened longitudinal, flushed with cold PBS, and pinned on Silicon plates for overnight postfixation in 4% PFA. Incubation of the primary antibodies (CD31, AF3628, R&D, 1:200; Lyve‐1, 103‐PA50AG, ReliaTech, 1:400; VE‐Cadherin, AF1002, R&D, 1:200) has been performed for 24–48 h. For analysis of the villus vasculature, we focused on areas surrounding the most distal Peyer’s Patches. Images were acquired using a Zeiss LSM 800 equipped with a 20×/0.8 objective.

#### Peyer’s patches

As above, perfused intestine segments containing Peyer’s Patches were pinned on silicon plates. Villi were removed by gentle scraping with the blunt end of a forcep, and staining of wholemount tissue has been performed as before (Bernier‐Latmani & Petrova, 2016).

### Immunostaining on cryosections

#### Cdh5(PAC)‐CreERT2 mice x ROSA^mTmG^

Cryosections (10–40 µm) from optimal cutting temperature (OCT) embedded tissue were stored at −20°C until further usage. Heat‐induced antigen retrieval with citrate buffer was performed for brain and lung tissue. Antigen retrieval with methanol was performed for the liver. No antigen retrieval was required for heart and intestine. Afterward, sections were blocked for 1 h in 1% BSA/PBS/0.3% triton. Primary antibodies (CD31, AF3628, R&D, 1:200; IsolectinGS‐IB4, I21413, Invitrogen, 1:200; Lyve‐1, 103‐PA50AG, ReliaTech, 1:300) were incubated for 24–48 h at 4°C in blocking solution. Afterward, sections were washed with PBS and incubated with appropriate secondary antibodies and DAPI or TOPRO for nuclear counterstaining in blocking solution for 2 h at room temperature or overnight at 4°C. After washing again, sections were mounted with Fluoromount‐G™ (Linaris). Images were acquired using a Zeiss LSM 800 equipped with a 20×/0.8 objective.

#### Immune cell stainings in Casp8^ECko^ mice

3–4 μm thick sections were prepared from fresh frozen, cryopreserved intestinal “Swiss‐rolls” embedded in OCT compound. Sections were hydrated and fixed in 4% PFA and subjected to Avedin/Biotin blocking (Vector laboratories) and blocked again using 1× commercial Immunoblock (Roth GmbH). Endogenous peroxidases were blocked using a solution of 0.3% H_2_O_2_ in PBS. The sections were then incubated with appropriate primary antibodies (CD4, BD Pharmingen, 1:100; MPO, Abcam, 1:200; F4/80, eBiosciences, 1:1,000) overnight. The sections were washed thrice in PBS‐T, and the antigen‐bound primary antibodies were detected using appropriate Horse Radish Peroxidase‐conjugated secondary antibodies followed by the Tyramide signal amplification (Thermo Fisher). Nuclei were counterstained with Hoechst 33342 (Thermo Fisher). Images were acquired on the Leica DMI4000 inverted fluorescence microscope.

#### TUNEL staining

10–20 µm thick sections were prepared from fresh frozen, cryopreserved intestinal “Swiss‐rolls” or Peyer’s Patches embedded in OCT compound. Sections were fixed in 4% PFA, and TUNEL staining has been performed according to the manufacturer’s instructions (Roche), followed by CD31 co‐staining as described above. Images were acquired using a Zeiss LSM 800 equipped with a 20×/0.8 objective.

### Immunostaining on paraffin sections

Mice were sacrificed by cervical dislocation and organs were stored in 4% PFA until tissue dehydration and paraffin embedding. For small intestine analysis, 3–4 µm thick sections of Swiss roll preparations of the ileum were dried overnight at 37°C and rehydrated in a decreasing alcohol gradient. Endogenous peroxidases were quenched with 3% H_2_O_2_ in peroxidase blocking buffer (0.04 M sodium citrate, 0.121 M disodium phosphate, 0.03 M sodium azide) for 15 min. After washing with tap H_2_O, heat‐induced antigen retrieval in citrate buffer or EDTA was performed. After washing with PBS, slides were blocked for 1 h at RT in TNB‐buffer (0.1 M Tris, 0.15 M sodium chloride, 2.5% milk powder). Primary antibodies (E‐Cadherin, 612130; BD Biosciences; CD45, 553080, BD Pharmingen, 1:100; NIMP‐R14, Novus Biologicals) were incubated overnight at 4°C in TNB‐buffer supplemented with 20% horse serum (Vector Laboratories). The next day, after washing with PBS, the appropriate biotinylated secondary antibodies were incubated for 45 min at RT in 1.5% BSA/PBS. Signal amplification was performed with the TSA^®^ biotin detection kit (PerkinElmer). Chromogenic stainings were performed using the liquid DAB^+^ substrate chromogen system (Dako/Agilent) according to the manufacturer’s instructions. Afterward, samples were briefly counterstained with hematoxylin (2–3 s), dehydrated in an increasing ethanol gradient, and mounted with Eukitt^®^.

### Fluorescent in situ hybridization

Detection of bacteria in small intestine paraffin sections was performed by FISH using the universal bacterial oligonucleotide probe EUB‐338 (5′‐GCT GCC TCC CGT AGG AGT‐3′ with Cyanine‐3 5′‐modification; biomers). In brief, deparaffinized sections were incubated with the probe in formamide containing hybridization buffer for 90 min at 46°C, as previously described (Snel *et al*, 1995).

### Microbiome sequencing

For marker gene‐based microbiome analysis, the V4 regions (515F‐806R) of the bacterial 16S rRNA genes were amplified using the NEBNext Q5 Hot Start Hifi PCR Master Mix (New England Biolabs, Frankfurt am Main, Germany) using protocols established in the Earth microbiome project (https://earthmicrobiome.org/protocols‐and‐standards/16s/). Amplified fragments were purified with AMPure XP Beads (Beckmann Coulter GmbH, Krefeld, Germany), pooled in equimolar ratios and analyzed by 2 × 151 bp paired‐end sequencing on an Illumina MiSeq device (Illumina Inc., San Diego, USA). Raw fastq files were then imported and analyzed in QIIME2 v2021.8 (Bolyen *et al*, 2019) with Dada2 (Callahan *et al*, 2016) as the method for quality control, dereplication, and sub‐operating taxonomic unit/amplicon sequence variant (sOTU/ASV) table generation. The SILVA (Quast *et al*, 2013) small subunit database release 138 was used at a 99% similarity cutoff for taxonomic classification.

### Image analysis

All image analysis was performed using the NIH ImageJ software and blind to experimental conditions. The percentage of active recombinase expressing ECs in organs of Cdh5(PAC)‐CreERT2 mice x ROSA^mTmG^ was calculated by measuring the GFP^+^ area per CD31^+^ or IsoB4^+^ vessel area. The villus vessel area was calculated by measuring the CD31^+^ vessel area normalized to the villus area. For every animal, at least 20–30 villi were analyzed. Lacteal length was calculated by measuring the length of the lacteal (starting at the villus base) divided by the length of the blood vessel capillary network. Lymphatic vessel density in Peyer’s Patch wholemounts was calculated by measuring the Lyve‐1^+^ area normalized to the Peyer’s Patch area. CD45^+^ cells were detected by deconvolution of DAB^+^/H&E‐stained sections, and DAB^+^ (CD45^+^) cells in the submucosa were automatically counted with a customized macro in ImageJ. The total number of cells was normalized to the tissue area. For every animal, 20‐30 pictures were analyzed. The number of dead cells has been calculated by manually counting TUNEL^+^ cells colocalizing with (i) the epithelial layer (determined by using DAPI staining), (ii) endothelium (by colocalization with CD31 staining), and (iii) stromal compartment (by counting all other TUNEL^+^ cells in the villus).

### Histopathological score

Mucosal hemorrhages were calculated per mouse/per lesion. A modified histopathological score (Welz *et al*, 2011) was applied on hematoxylin‐stained intestine sections (3–4 µm) by an experienced pathologist blind to genotypes or treatment. A score for each parameter was applied for two different parameters: (i) small intestinal inflammation: 0, no inflammatory infiltrate in the lamina propria; 1, increased presence of inflammatory cells between the crypts; 2, inflammatory infiltrate extending into the villi; 3, extension of inflammatory infiltrate throughout the lamina propria. (ii) tissue damage: 0 epithelial barrier intact; 1, mild superficial damage of the epithelium; 2: moderate damage with detectable re‐epithelization; 3, loss of epithelial barrier and crypt structure, reactive epithelial cell changes. The combined score was calculated by adding the score for each parameter. A modified score was applied to grade DSS‐induced colitis development: a score for each parameter was applied for three different parameters: (i) number of total ulcerative lesions of each samples [0 > Score 0; 1–5 > Score 1; 6–10 > Score 3); (ii) extent of inflammation: mild [Score 1], moderate [2], severe [3]; iii) extent of regenerative epithelial changes at the border/surrounding of the ulcerative lesions: mild [Score 1], moderate [Score 2], severe [Score 3]. The combined score was calculated by adding the score for each parameter.

Exemplary pictures illustrating both scoring schemes can be found in Appendix Fig S7.

### Synopsis picture

The synopsis picture was created with BioRender.com.

### Experimental design and statistical analysis

At least three mice per group were analyzed in each experiment, and at least two independent experiments have been performed. All experimenters were blinded both during the execution and analysis of experiments. In experiments that required treatments (e.g., injections of substances etc.), mice of each sex were equally distributed between experimental groups (no randomization). Whenever possible, Cre^−^ and Cre^+^ mice were co‐housed to compensate for differences in the microbiota. Casp8^ECko^ mice that presented less than 50% recombination (as determined by qPCR analysis of isolated lung ECs) were excluded from the study.

Statistical analysis was performed using GraphPad Prism (version 7.0 and 8.0; GraphPad Software Inc.). All data is expressed as mean ± SEM. For comparison between two groups, unpaired Student’s *t*‐test (with Welch’s correction if variance within groups was unequal) was used. For comparisons between multiple groups, one‐way ANOVA with Tukey’s multiple comparison, two‐way ANOVA using the Sidak or Holm–Sidak method, or multiple *t*‐tests with the Holm–Sidak method were performed. For survival analysis, the Log‐Rank test was applied. Significance between weight loss curves was determined using the curve comparison function of GraphPad Prism. Alpha and beta diversity of the intestinal microbiome was determined with Permanova test and Mann–Whitney *U* test. Ward clustering using Euclidean distances was used to generate heat maps. Outlier detection has been performed using the Outlier calculator (GraphPad Prism) with an Alpha = 0.05 significance level. Statistical significance was defined as follows: *P < 0.05, **P < 0.01 ***P < 0.001, ****P < 0.0001. Individual *P*‐values are listed in Appendix Table S2.

## Author contributions

**Nathalie Tisch:** Conceptualization; Data curation; Formal analysis; Supervision; Funding acquisition; Validation; Investigation; Visualization; Methodology; Writing – original draft; Writing – review & editing. **Carolin Mogler:** Investigation; Writing – review & editing. **Ana Stojanovic:** Investigation; Methodology. **Robert Luck:** Validation; Investigation. **Emilia A Korhonen:** Data curation; Methodology. **Alexander Ellerkmann:** Investigation. **Heike Adler:** Data curation. **Mahak Singhal:** Data curation; Formal analysis; Visualization. **Géza Schermann:** Investigation; Visualization; Methodology. **Lena Erkert:** Investigation. **Jay Patankar:** Data curation; Formal analysis. **Andromachi Karakatsani:** Investigation. **Anna‐Lena Scherr:** Investigation; Methodology. **Yaron Fuchs:** Resources. **Adelheid Cerwenka:** Resources; Supervision. **Stefan Wirtz:** Resources; Investigation; Visualization; Writing – review & editing. **Bruno Christian Köhler:** Resources; Supervision; Methodology. **Hellmut, G Augustin:** Resources; Supervision. **Christoph Becker:** Conceptualization; Resources; Supervision; Methodology. **Thomas Schmidt:** Resources; Supervision. **Carmen Ruiz de Almodóvar:** Conceptualization; Resources; Supervision; Funding acquisition; Writing – original draft; Project administration; Writing – review & editing.

In addition to the CRediT author contributions listed above, the contributions in detail are:

NT designed, performed, and supervised initial experiments, analyzed data, and helped in funding acquisition; CM provided histopathological analysis and interpretation; AS performed flow cytometry experiments; RL helped with *in vivo* experiments, iv injections, and analyzed data; EAK performed *in vivo* experiments and imaging; AE performed experiments; AK performed iv injections of tracers and TNF‐α; HA performed qPCR experiments and imaging; GS analyzed results from the proteome profiler assay and transcriptome database; LE performed bacterial FISH and epithelial barrier stainings; MS performed Flow Cytometry experiments; JVP performed immune cell stainings; A‐LS and BCK performed colon endoscopy; YF provided infrastructure; SW performed 16S ribosomal RNA gene sequencing; AC, CB, HGA, and TS supervised specific parts of the project and provided funding; CRA supervised all stages of the project and acquired funding; NT and CRA wrote the manuscript; all authors discussed and interpreted the data and gave input to the written manuscript.

## Disclosure and competing interests statement

The authors declare that they have no conflict of interest.

## Supporting information



Appendix

Expanded View Figures PDF

Movie EV1

Movie EV2

Source Data for Expanded View

Source Data for Figure 1

Source Data for Figure 2

Source Data for Figure 3

Source Data for Figure 4

Source Data for Figure 5

Source Data for Figure 6

Source Data for Figure 7

## Data Availability

This study includes no data deposited in external repositories.
