# Supplementary material for: Caspase‐8 in endothelial cells maintains gut homeostasis and prevents small bowel inflammation in mice
Source: EMBO Mol Med. 2022 May 2;14(6):e14121. doi: 10.15252/emmm.202114121 (PMC9174885; doi:10.15252/emmm.202114121)
Supplement: Supplementary file 1 — Appendix [file EMMM-14-e14121-s002.pdf]

Table of Content

1. Appendix Supplementary Figures S1-S7

2. Appendix Supplementary Figure Legends

3. Appendix Table S1

4. Appendix Table S2

1. Appendix Supplementary Figures S1-S7

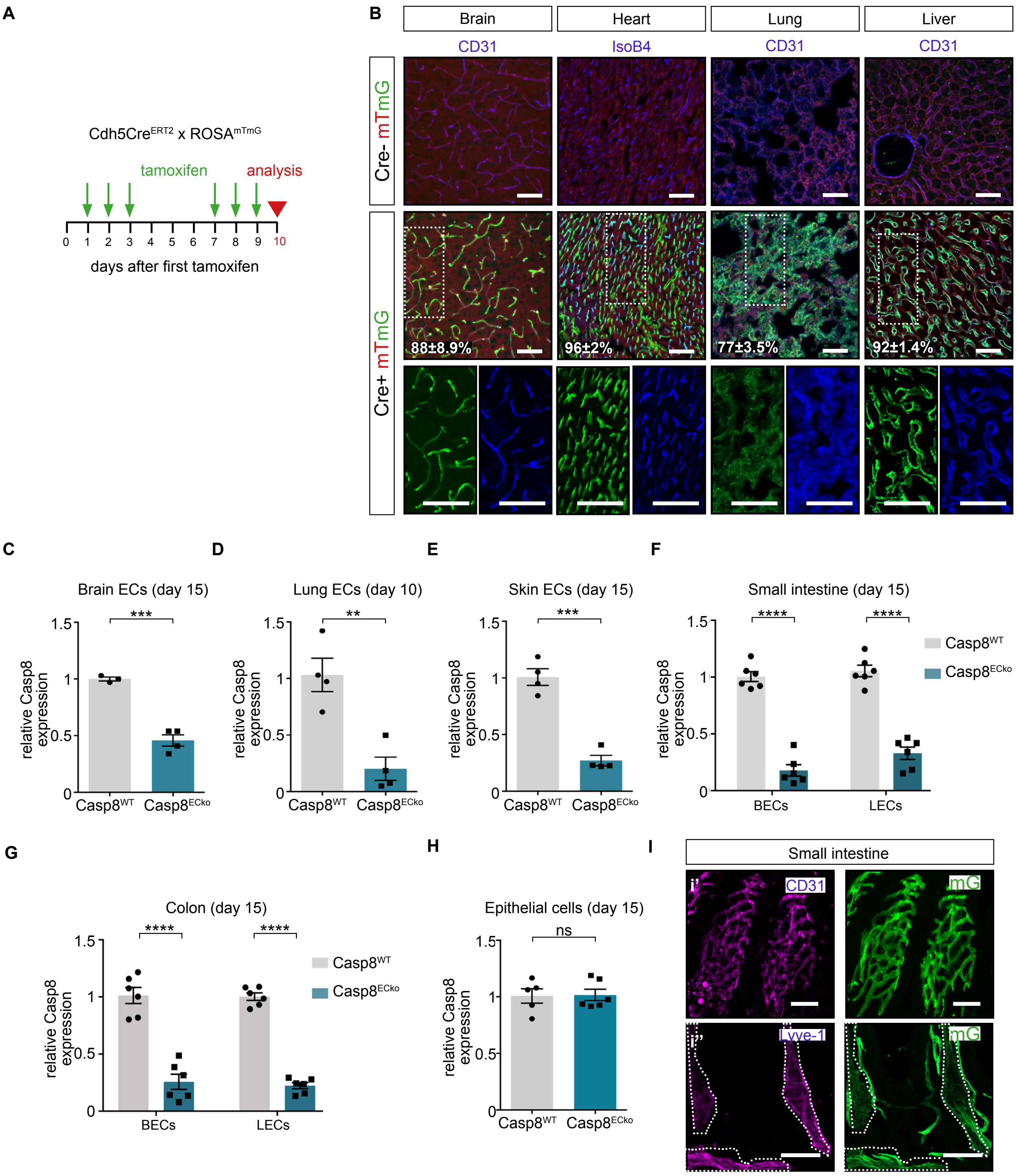

Appendix Fig. S1:

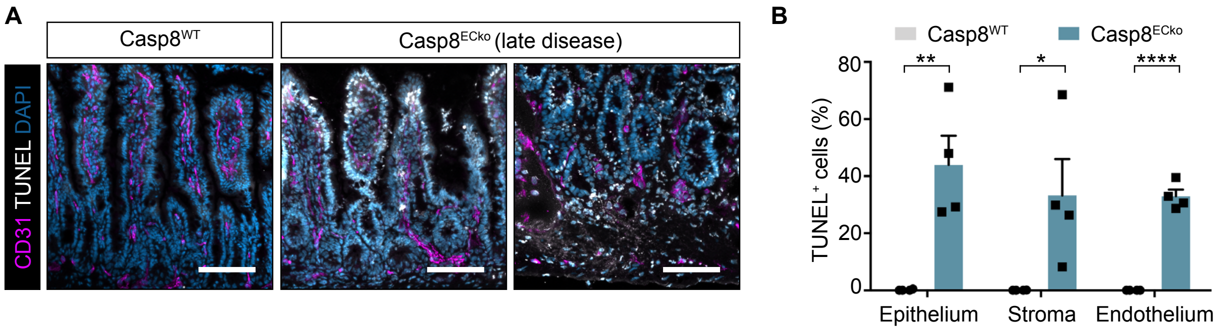

Appendix Fig. S2:

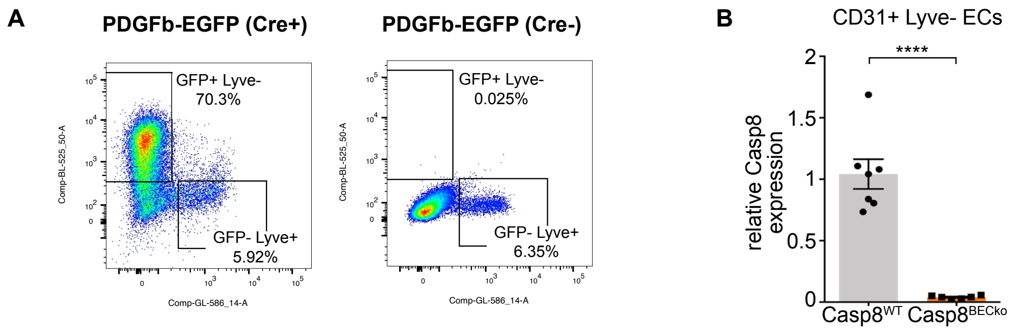

Appendix Fig. S3:

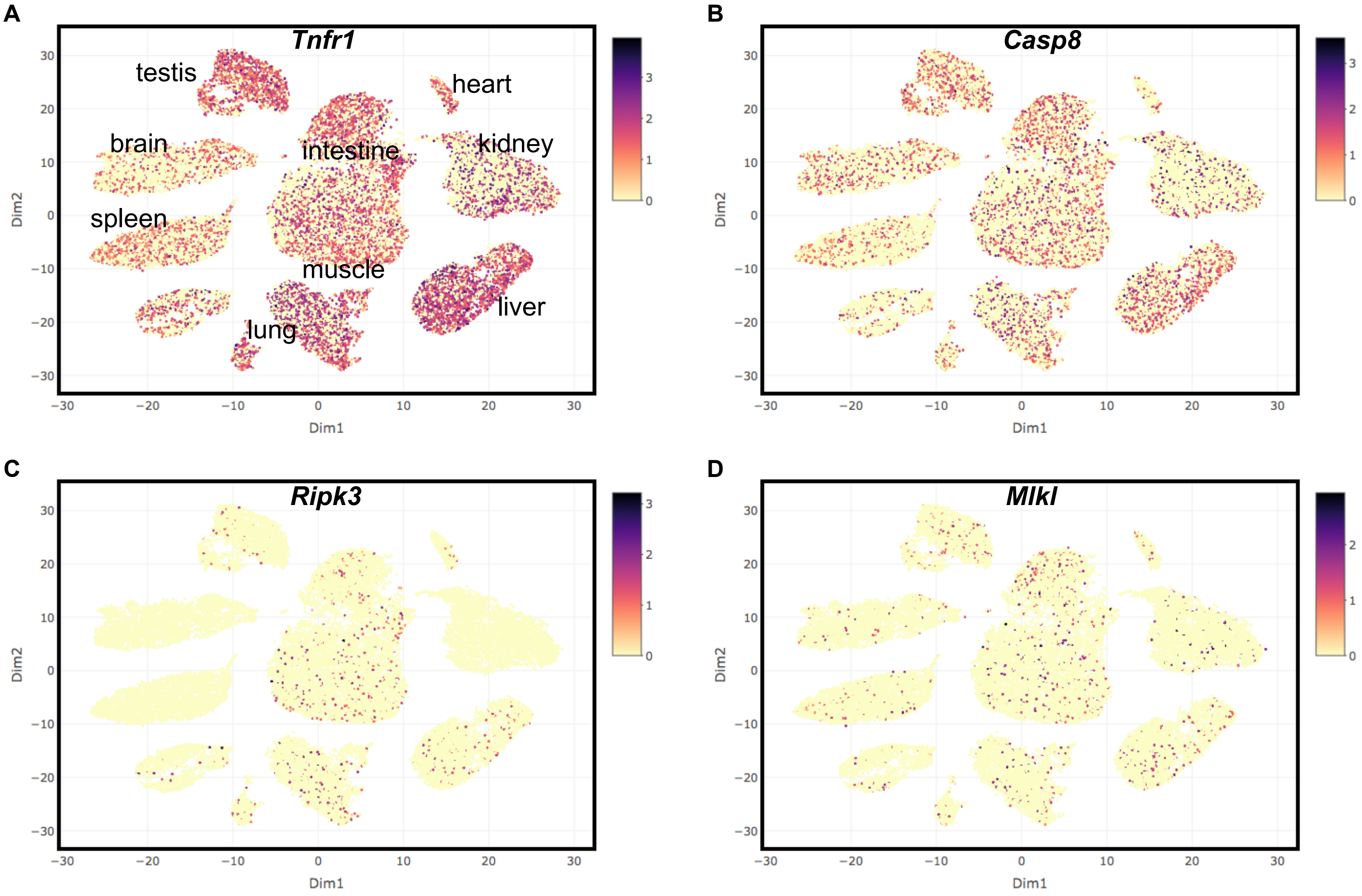

Appendix Fig. S4:

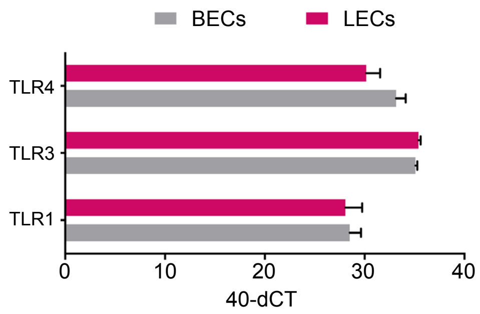

Appendix Fig. S5:

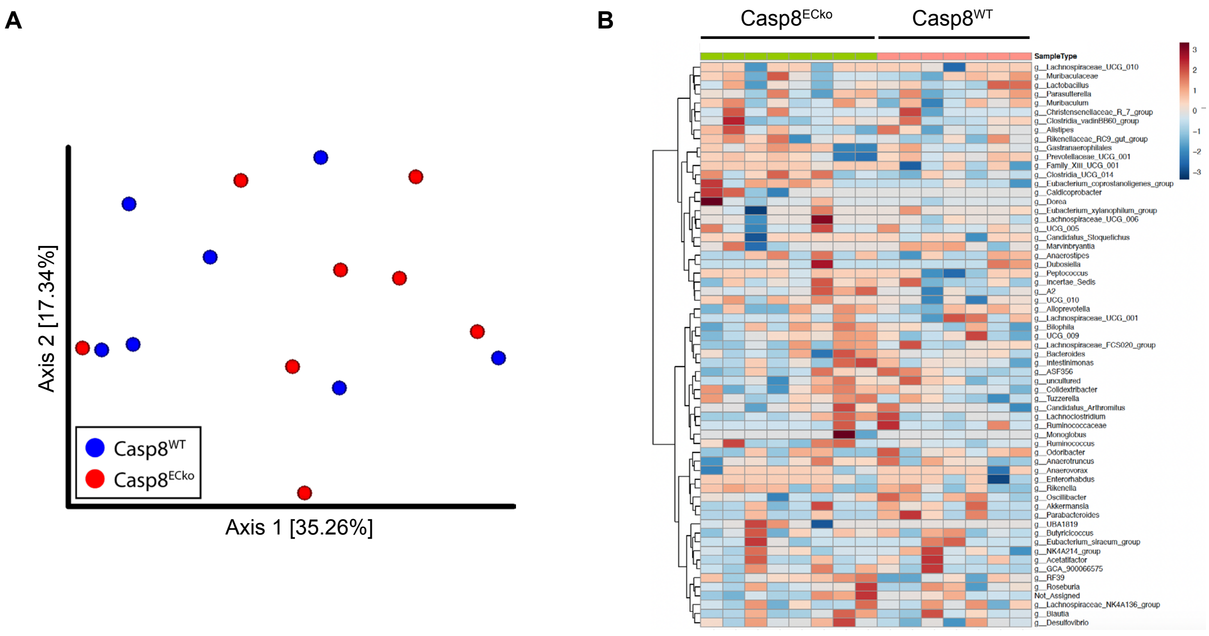

Appendix Fig. S6:

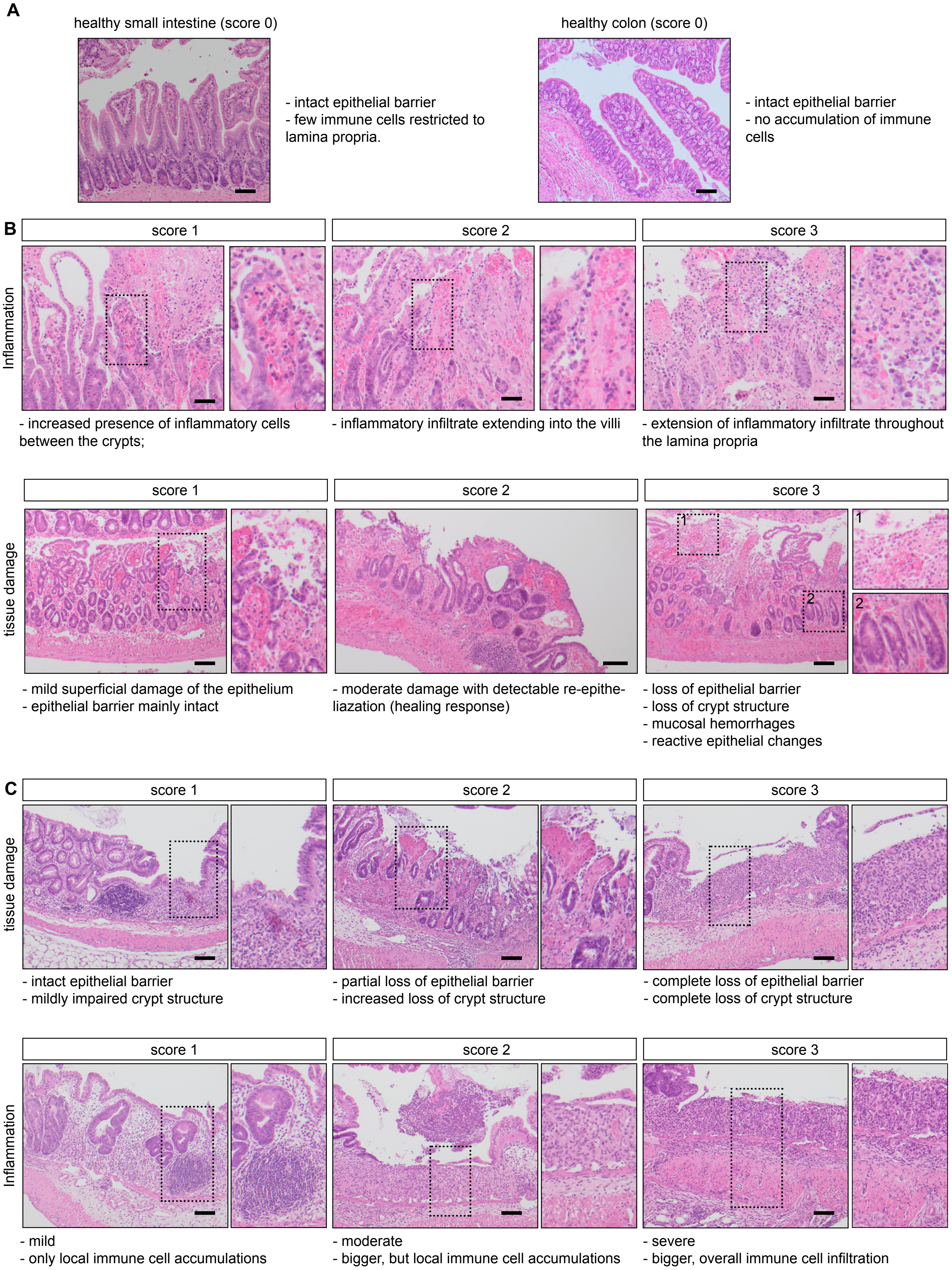

Appendix Fig. S7:

2. Appendix Supplementary Figure Legends

Appendix Fig. S1: Efficient Cre recombination and Casp8 deletion in ECs from different organs in Casp8^{ECko} mice.

A. Scheme showing the tamoxifen treatment protocol and the day of analysis for Cre activity in Cdh5Cre^{ERT2} x ROSA^{mTmG} reporter mice.

B. Representative images of vessel staining (CD31 or IsoB4) and mT/mG signal in the brain, heart, lung and liver of Cre+ and Cre- mice. Boxed areas are shown in higher magnifications and split channels. Numbers indicate the percentage of CD31+/GFP+ vasculature (n=3 Cre+). Scale bars: 100µm.

C-G. QPCR quantification of Casp8 expression in ECs from brain (C; n= 3 WT, 4 ECko; two tailed unpaired Student's t-test), lung (D; n= 4 WT, 4 ECko; two tailed unpaired Student's t-test), skin (E; n= 4 WT, 4 ECko; two tailed unpaired Student's t-test), small intestine (F; n= 6 WT, 6 ECko; two-way ANOVA with Sidak´s multiple comparison) and colon (G; n= 6 WT, 6 ECko; two-way ANOVA with Sidak´s multiple comparison) of Casp8^{WT} and Casp8^{ECko} mice.

H. QPCR quantification of Casp8 expression in isolated small intestinal epithelial cells from Casp8^{WT} and Casp8^{ECko} mice (n= 5 WT, 6 ECko; two tailed unpaired Student's t-test).

I. Representative pictures of CD31 (i') and Lyve-1 (i'') staining and mG signal in the small intestine of Cdh5Cre^{ERT2} x ROSA^{mTmG} reporter mice (n= 3 Cre+ mice) showing GFP+ cells in blood and lymphatic ECs. Scale bars: 50µm.

Data information: All data is shown as mean ± SEM. ** $P < 0.01$, *** $P < 0.001$, **** $P < 0.0001$; ns: not significant.

Appendix Fig. S2: Characterization of cell death upon Casp8 deletion in ECs.

A. Representative images of CD31, TUNEL and DAPI staining of sections of small intestine from $Casp8^{WT}$ and $Casp8^{ECko}$ mice at a late disease stage. Scale bars: 100µm.

B. Quantification of TUNEL+ cells in $Casp8^{WT}$ and $Casp8^{ECko}$ small intestines at a late disease stage (n= 4 WT, 4 ECko, multiple t-tests with Holm-Sidak method).

Data information: All data is shown as mean ± SEM. * P<0.05, ** P <0.01, *** P <0.001, **** P <0.0001; ns: not significant.

Appendix Fig. S3: Expression of PDGFb-EGFP is restricted to BECs in the small intestine.

A: Assessment of EGFP expression in Pdgfb-iCreER+ and Pdgfb-iCreER- mice by flow cytometry, showing that EGFP expression was absent from the Lyve1+ population (plots are representative of n=6 mice per group).

B: Casp8 expression levels determined by qPCR in small intestinal BECs of $Casp8^{WT}$ and $Casp8^{BECko}$ mice (n=6 mice per genotype; two tailed unpaired Student's t-test).

Data information: All data is shown as mean ± SEM. **** P<0.0001.

Appendix Fig. S4: Genes associated to the necroptosis signaling pathway are equally expressed in ECs of different organs.

A-D. *Tnfr1* expression (A). *Casp8* expression (B). *Ripk3* expression (C). *Mlkl* expression (D). Note that except of brain, spleen and kidney, all molecules are co-expressed in different EC clusters.

Data information: Data has been extracted from a publicly available scRNA-Seq database (Kalucka et al. 2020; https://endotheliomics.shinyapps.io/ec_atlas/).

Appendix Fig. S5: TLRs are expressed in small intestinal BECs and LECs. QPCR expression analysis of the main TLRs in sorted small intestinal BECs and LECs (n= 6 mice).

Data information: All data is shown as mean ± SEM.

Appendix Fig. S6: Microbiome sequencing of Casp8^{ECko} early disease intestine.

A-B. Principal component analysis (PcoA) of weighted UniFrac distances (A) and heat map (B) of small intestinal microbiome analysis between Casp8^{ECko} and Casp8^{WT} mice at an early disease stage (n= 8 WT, 7 ECko, A: Permanova test and Mann-Whitney U test)

Appendix Fig. S7: Illustration of the different parameters of the histopathological score for small intestine and DSS-induced colitis samples.

A. Representative pictures of healthy small intestine and colon tissue. Scale bars: 100µm.

B. Exemplary pictures of different severities (score 1-3) in the small intestine in the two evaluated parameters: inflammation and tissue damage. Insets highlight the specific features explained below the pictures. Scale bar: 100µm.

C. Exemplary pictures of different severities (score 1-3) in the DSS-induced colitis model in the two evaluated parameters: inflammation and tissue damage. Insets highlight the specific features explained below the pictures. Scale bar: 100µm.

3. Appendix Table S1

qPCR primers

Gene	***Primer***
Mouse *Angpt2*	
Mouse *Casp8*	1. 5’-GGTCATGCTCTTTAAGCTCTCA-3’<br>2. 5’-TCATCTTCCAGCTTACATTTGG-3’
Mouse *Ccl2*	1. 5’-GATGCAGTTAACGCCCCACT-3’<br>2. 5’- ACCCATTCCTTCTTGGGGTC-3’
Mouse *c-myc*	1. 5’-CAGCGACTCTGAAGAAGAGCA-3’<br>2. 5’-TTGTGCTGGTGAGTGGAGAC-3’
Mouse *Cxcl1*	1. 5’-ACTCAAGAATGGTCGCGAGG-3’<br>2. 5’-GTGCCATCAGAGCAGTCTGT-3’
Mouse *Cxcl5*	1. 5’-CCCTACGGTGGAAGTCATAGC-3’<br>2. 5’-TTCACTGGGGTCAGAGTCCT-3’
Mouse *Icam1*	1. 5’-AAGGTGGTTCTTCTGAGCGG-3’<br>2. 5’-TCCAGCCGAGGACCATACAG-3’
Mouse *Il1b*	1. 5’-TGCCACCTTTTGACAGTGATG-3’<br>2. 5’-AAGGTCCACGGGAAAGACAC-3’
Mouse *Il1rn*	1. 5’-TCACCAACACACCGGAAGAG-3’<br>2. 5’-GCCAGAGTGATCAGGCAGTT-3’
Mouse *Il6*	1. 5’-CCCCAATTTCCAATGCTCTCC-3’<br>2. 5’-CGCACTAGGTTTGCCGAGTA-3’
Mouse *Ki67*	1. 5’-CGGCCAGAGCTAACTTGCG-3’<br>2. 5’-TTCAATACTCCTTCCAAACAGGCA-3’
Mouse *Mlkl*	1. 5’-AGATCCCATTTGAAGGCTGTGA-3’<br>2. 5’-TGCCAGAAAGACTCCTACCG-3’
Mouse *Ripk3*	1. 5’-GCCTTCCTCTCAGTCCACAC-3’<br>2. 5’-ACGCACCAGTAGGCCATAAC-3’
Mouse *Tlr1*	1. 5’-GTGTGCAGCTGATTGCTCAT-3’<br>2. 5’CAAACCGATCGTAGTGCTGA-3’
Mouse *Tlr 3*	1. 5’-ATATGCGCTTCAATCCGTTC-3’<br>2. 5’CAGGAGCATACTGGTGCTGA-3’
Mouse *Tlr 4*	1. 5’-TTTGCTGGGGCTCATTCACT-3’<br>2. 5’GACTCGGCACTTAGCACTGT-3’
Mouse *TNFA*	1. 5’-GACGTGGAAGTGGCAGAAGAG-3’<br>2. 5’-TGCCACAAGCAGGAATGAGA-3’
Mouse *Tnfr1*	1. 5’-AAGGCTGGAAAGCCCCTAAC-3’<br>2. 5’-GAACTAAAGCCTGGGGTGCT-3’
Mouse *Vcam1*	1. 5’-GTCACGGTCAAGTGTTTGGC-3’<br>2. 5’-TCCTGGGAGAGATGTAGACTTGT-3’
Mouse *Vegfa*	1. 5’-CGTTCACTGTGAGCCTTGTT-3’<br>2. 5’-CTTGGCTTGTCACATCTGCA-3’
Human *Mlkl*	1. 5’-CCCTCAGGTAGGGATCGGG-3’<br>2. 5’-TTTCCATGCCTTCGCGCCT-3’
Human *TNFA*	1. 5’-ATCTTCTCGAACCCCGAGTGA-3’<br>2. 5’-CGGTTCAGCCACTGGAGCT-3’
Human *Tnfr1*	1. 5’-CCCGAGTCTCAACCCTCAAC-3’<br>2. 5’-ATTCCCACCAACAGCTCCAG-3’
Human *Ripk3*	1. 5’-CAAGATCGTAAACTCGAAGG-3’<br>2. 5’-CCGTTCTCCATGAATTTAGT-3’
16S rDNA Bacteroidetes 1	1. 5’-GGAGAGTACCCGGAGAAAAAGC-3’<br>2. 5’-TTCCGCATACTTCTCGCCCA-3’
16S rDNA Bacteroidetes 2	1. 5’-CCAGCAGCCGCGGTAATA-3’<br>2. 5’-CGCATTCCGCATACTTCTC-3’
16S rDNA Firmicutes	1. 5’-TGAAACTYAAAGGAATTGACG-3’<br>2. 5’-ACCATGCACCACCTGTC-3’

4. Appendix Table S2

P values for Fig. 1-6, Fig. EV 1-5, Appendix Fig. S1, S2

Figure	Comparison	*P* value
Fig. 1B	Casp8^{WT} vs. Casp8^{ECko}	0,0032
Fig. 1C	Casp8^{WT} vs. Casp8^{ECko}	<0,0001
Fig. 1F	Casp8^{WT} vs. Casp8^{ECko}	>0,9999
Fig. 1G	Casp8^{WT} vs. Casp8^{ECko}	<0,0001
Fig. 1H	Casp8^{WT} vs. Casp8^{ECko}	0,0003
Fig. 2A	Ccl2: Casp8^{WT} vs. Casp8^{ECko}	0,00004
Fig. 2A	Il1b: Casp8^{WT} vs. Casp8^{ECko}	0,003908
Fig. 2A	TNA: Casp8^{WT} vs. Casp8^{ECko}	0,024796
Fig. 2A	Il1rn: Casp8^{WT} vs. Casp8^{ECko}	0,00137
Fig. 2B	VCAM: Casp8^{WT} vs. Casp8^{ECko}	0,001718
Fig. 2B	ICAM: Casp8^{WT} vs. Casp8^{ECko}	0,014235
Fig. 2B	VEGF: Casp8^{WT} vs. Casp8^{ECko}	0,001437
Fig. 2B	Ang2: Casp8^{WT} vs. Casp8^{ECko}	0,000059
Fig. 2C	Complement Factor D: Casp8^{WT} vs. Casp8^{ECko}	0,817708
Fig. 2C	Complement Component C5/C5a: Casp8^{WT} vs. Casp8^{ECko}	0,021638
Fig. 2C	C-Reactive Protein: Casp8^{WT} vs. Casp8^{ECko}	0,023798
Fig. 2D	EGF: Casp8^{WT} vs. Casp8^{ECko}	0,016627
Fig. 2D	HGF: Casp8^{WT} vs. Casp8^{ECko}	0,000003
Fig. 2D	Amphiregulin: Casp8^{WT} vs. Casp8^{ECko}	0,000984
Fig. 2D	IGFBP-2: Casp8^{WT} vs. Casp8^{ECko}	0,013671
Fig. 2D	IGFBP-5: Casp8^{WT} vs. Casp8^{ECko}	0,000239
Fig. 2F	Casp8^{WT} vs. Casp8^{ECko}	0,0074
Fig. 2G	c-myc: Casp8^{WT} vs. Casp8^{ECko}	0,0062
Fig. 2G	Ki67: Casp8^{WT} vs. Casp8^{ECko}	0,0085
Fig. 3B	Casp-8^{WT} vs. Casp8^{ECko} no hem	0,0112
Fig. 3B	Casp-8^{WT} vs. Casp8^{ECko} mild hem	0,0152
Fig. 3B	Casp-8^{WT} vs. Casp8^{ECko} strong hem	<0,0001
Fig. 3E	Casp8^{WT} vs. Casp8^{ECko}	0,0018
Fig. 3F	Casp8^{WT} vs. Casp8^{ECko}	0,0125
Fig. 3H	Intestine: Casp8^{WT} vs. Casp8^{ECko}	0,0003
Fig. 3H	Brain: Casp8^{WT} vs. Casp8^{ECko}	>0,9999
Fig. 4B:	Epithelium: Casp8^{WT} vs. Casp8^{ECko}	0,008097
Fig. 4B:	Stroma: Casp8^{WT} vs. Casp8^{ECko}	0,027191
Fig. 4B:	Endothelium: Casp8^{WT} vs. Casp8^{ECko}	0,001177
Fig. 4D:	Casp8^{WT}/MLKL^{ko} vs. Casp8^{ECko}/MLKL^{ko}	>0,9999
Fig. 4E:	Casp8^{WT}/MLKL^{ko} vs. Casp8^{ECko}/MLKL^{ko}	0,1210
Fig. 4H:	Casp8^{WT}/MLKL^{ko} vs. Casp8^{ECko}/MLKL^{ko}	0,3060
Fig. 4I:	Casp8^{WT}/MLKL^{ko} vs. Casp8^{ECko}/MLKL^{ko}	0,4110
Fig. 4J:	Casp8^{WT}/MLKL^{ko} vs. Casp8^{ECko}/MLKL^{ko}	0,1738
Fig. 4K:	Casp8^{WT}/MLKL^{ko} vs. Casp8^{ECko}/MLKL^{ko}	0,8435
Fig. 4L:	Casp8^{WT}/MLKL^{ko} vs. Casp8^{ECko}/MLKL^{ko}	0,5301
Fig. 4N:	Casp8^{WT}/MLKL^{ko} vs. Casp8^{ECko}/MLKL^{ko}	0,8903
Fig. 4P:	Casp8^{WT}/MLKL^{ko} vs. Casp8^{ECko}/MLKL^{ko}	>0,9999
Fig. 4Q:	Casp8^{WT}/MLKL^{ko} vs. Casp8^{ECko}/MLKL^{ko}	>0,9999
Fig. 5C:	Casp8^{WT} vs. Casp8^{BECko}	>0,9999
Fig. 5F:	Casp8^{WT} vs. Casp8^{BECko}	>0,9999
Fig. 5G:	Casp8^{WT} vs. Casp8^{BECko}	>0,9999
Fig. 5I:	Casp8^{WT} vs. Casp8^{BECko}	0,2097
Fig. 5J:	Casp8^{WT} vs. Casp8^{BECko}	0,0677
Fig. 5L:	Casp8^{WT} vs. Casp8^{BECko}	0,3351
Fig. 6B:	Casp8^{WT} vs. Casp8^{ECko}	0,9731
Fig. 6E:	Casp8^{WT} vs. Casp8^{ECko}	0,0005

Fig. 6E:	Casp8^{Ecko} vs. Casp8^{ECko}/MLKL^{ko}	<0,0001
Fig. 6H:	Casp8^{WT} vs. Casp8^{ECko} + IgG	0,0003
Fig. 6H:	Casp8^{WT} vs. Casp8^{ECko} + Enbrel	0,0133
Fig. 6K:	Casp8^{WT} vs. Casp8^{ECko} + IgG	<0,0001
Fig. 6K:	Casp8^{WT} vs. Casp8$^{ECko +}$ Enbrel diseased	<0,0001
Fig. 6K:	Casp8^{WT} vs. Casp8$^{ECko +}$ Enbrel healthy	>0,9999
Fig. 7B:	Bacteroidetes 1: Control vs. Antibiotics	<0,0001
Fig. 7B:	Bacteroidetes 2: Control vs. Antibiotics	<0,0001
Fig. 7C:	Casp8^{WT} vs. Casp8^{ECko}	0,0001
Fig. 7C:	Casp8^{ECko} vs. Casp8^{ECko} antibiotics	0,0002
Fig. 7D:	Casp8^{WT} vs. Casp8^{ECko}	0,0001
Fig. 7D:	Casp8^{ECko} vs. Casp8^{ECko} antibiotics	0,0025
Fig. 7G:	Casp8^{WT} vs. Casp8^{ECko}	<0,0001
Fig. 7G:	Casp8^{ECko} vs. Casp8^{ECko} antibiotics	<0,0001
Fig. 7H:	Casp8^{WT} vs. Casp8^{ECko}	<0,0001
Fig. 7H:	Casp8^{ECko} vs. Casp8^{ECko} antibiotics	<0,0001
Fig. EV1C:	Casp8^{WT} vs. Casp8^{ECko}	>0,9999
Fig. EV1D:	Casp8^{WT} vs. Casp8^{ECko}	>0,9999
Fig. EV2D:	Casp8^{WT} vs. Casp8^{ECko}	0,0951
Fig. EV2F:	Casp8^{WT} vs. Casp8^{ECko}	0,0037
Fig. EV3A:	Casp8^{WT} vs. Casp8^{ECko}	>0,9999
Fig. EV4B:	Casp8^{WT} + Casp8^{WT} + IMQ	<0,0001
Fig. EV4B:	Casp8^{ECko} + Casp8^{ECko}+ IMQ	<0,0001
Fig. EV4B:	Casp8^{WT} + IMQ vs. Casp8^{ECko} + IMQ	0,1908
Fig. EV4C:	Casp8^{WT} + Casp8^{WT} + IMQ	0,0013
Fig. EV4C:	Casp8^{ECko} + Casp8^{ECko}+ IMQ	0,0025
Fig. EV4C:	Casp8^{WT} + IMQ vs. Casp8^{ECko} + IMQ	0,9680
Fig. EV4E:	Casp8^{WT} + Casp8^{WT} + IMQ	0,0023
Fig. EV4E:	Casp8^{ECko} + Casp8^{ECko}+ IMQ	0,0002
Fig. EV4E:	Casp8^{WT} + IMQ vs. Casp8^{ECko} + IMQ	0,8412
Fig. EV5B:	Casp8^{WT} vs. Casp8^{ECko}	0,3686
Fig. EV5C:	Casp8^{WT} vs. Casp8^{ECko}	>0,9999
Fig. EV5D:	Casp8^{WT} vs. Casp8^{ECko}	0,3714
Fig. EV5F:	Casp8^{WT} vs. Casp8^{ECko}	0,3715
Fig. EV5G:	TNFA: Casp8^{WT} vs. Casp8^{ECko}	0,270271
Fig. EV5G:	Ccl2: Casp8^{WT} vs. Casp8^{ECko}	0,933552
Fig. EV5G:	Il1b: Casp8^{WT} vs. Casp8^{ECko}	0,700199
Fig. EV5G:	Il16: Casp8^{WT} vs. Casp8^{ECko}	0,359654
Fig. EV5H:	Casp8^{WT} vs. Casp8^{ECko}	0,0371
Appendix Fig. S1C:	Casp8^{WT} vs. Casp8^{ECko}	0,0003
Appendix Fig. S1D:	Casp8^{WT} vs. Casp8^{ECko}	0,0037
Appendix Fig. S1E:	Casp8^{WT} vs. Casp8^{ECko}	0,0001
Appendix Fig. S1F:	BEC: Casp8^{WT} vs. Casp8^{ECko}	<0,0001
Appendix Fig. S1F:	LEC: Casp8^{WT} vs. Casp8^{ECko}	<0,0001
Appendix Fig. S1G:	BEC: Casp8^{WT} vs. Casp8^{ECko}	<0,0001
Appendix Fig. S1G:	LEC: Casp8^{WT} vs. Casp8^{ECko}	<0,0001
Appendix Fig. S1H:	Casp8^{WT} vs. Casp8^{ECko}	0,915
Appendix Fig. S2B:	Epithelium: Casp8^{WT} vs. Casp8^{ECko}	0,000008
Appendix Fig. S2B:	Stroma: Casp8^{WT} vs. Casp8^{ECko}	0,005076
Appendix Fig. S2B:	Endothelium: Casp8^{WT} vs. Casp8^{ECko}	0,039503
Appendix Fig. S3B:	Casp8^{WT} vs. Casp8^{BECko}	<0,0001
