# Supplementary material for: Caspase‐8 in endothelial cells maintains gut homeostasis and prevents small bowel inflammation in mice
Source: EMBO Mol Med. 2022 May 2;14(6):e14121. doi: 10.15252/emmm.202114121 (PMC9174885; doi:10.15252/emmm.202114121)
Supplement: Supplementary file 2 — Expanded View Figures PDF [file EMMM-14-e14121-s010.pdf]

Expanded View Figures

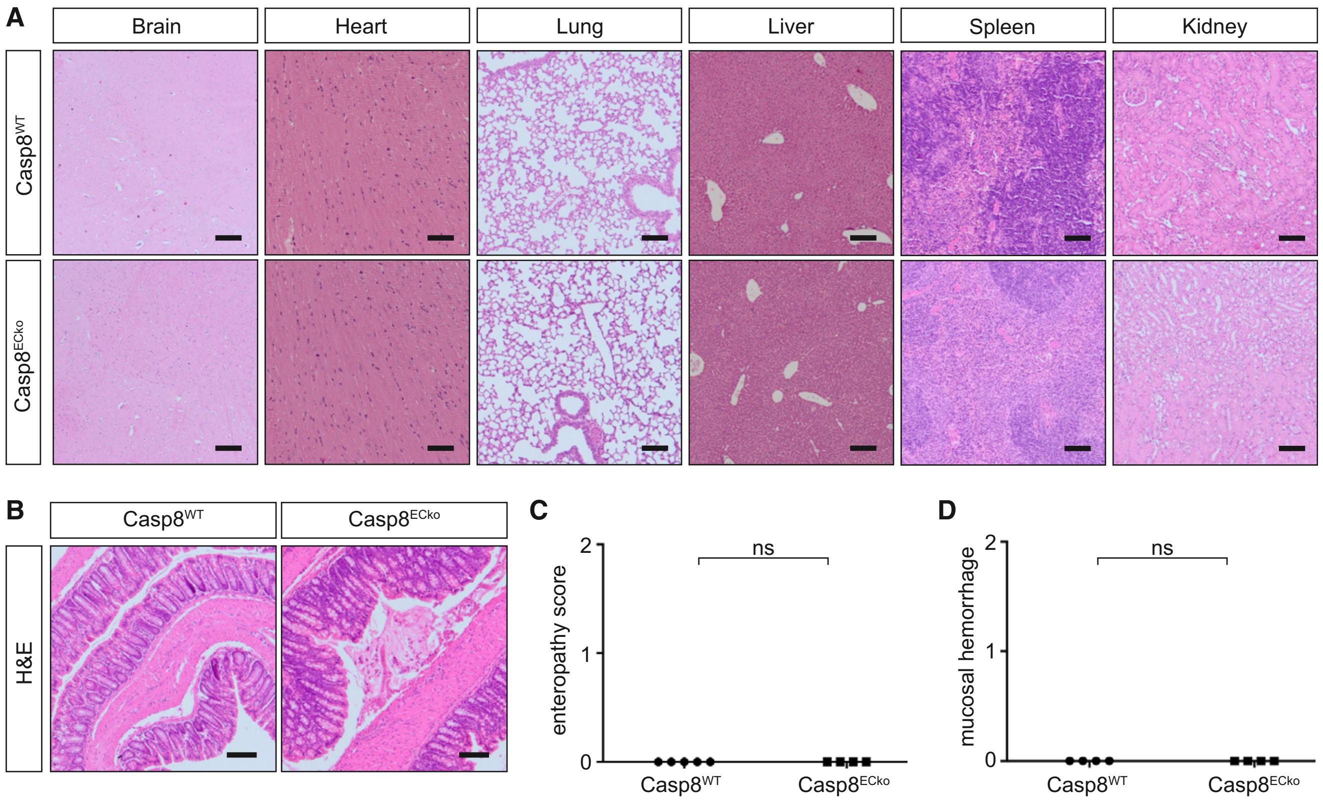

Figure EV1. Casp8^{ECko} mice do not present defects in other organs.

A Representative images of H&E staining of the indicated organs in Casp8^{WT} and Casp8^{ECko} mice at a late disease stage (n = 4–5 WT, 5 ECko). Scale bars 100 μm.

B–D Representative images of H&E staining of the large intestine (colon) (B). Quantification of intestinal pathology (C) and mucosal hemorrhages (D) in Casp8^{WT} and Casp8^{ECko} mice at a late disease stage (enteropathy score; n = 4 WT, 4 ECko; two-tailed unpaired Student's t-test). Scale bars: 100 μm.

Data information: All data is shown as mean ± SEM. ns: not significant.
Source data are available online for this figure.

 © 2022 The Authors

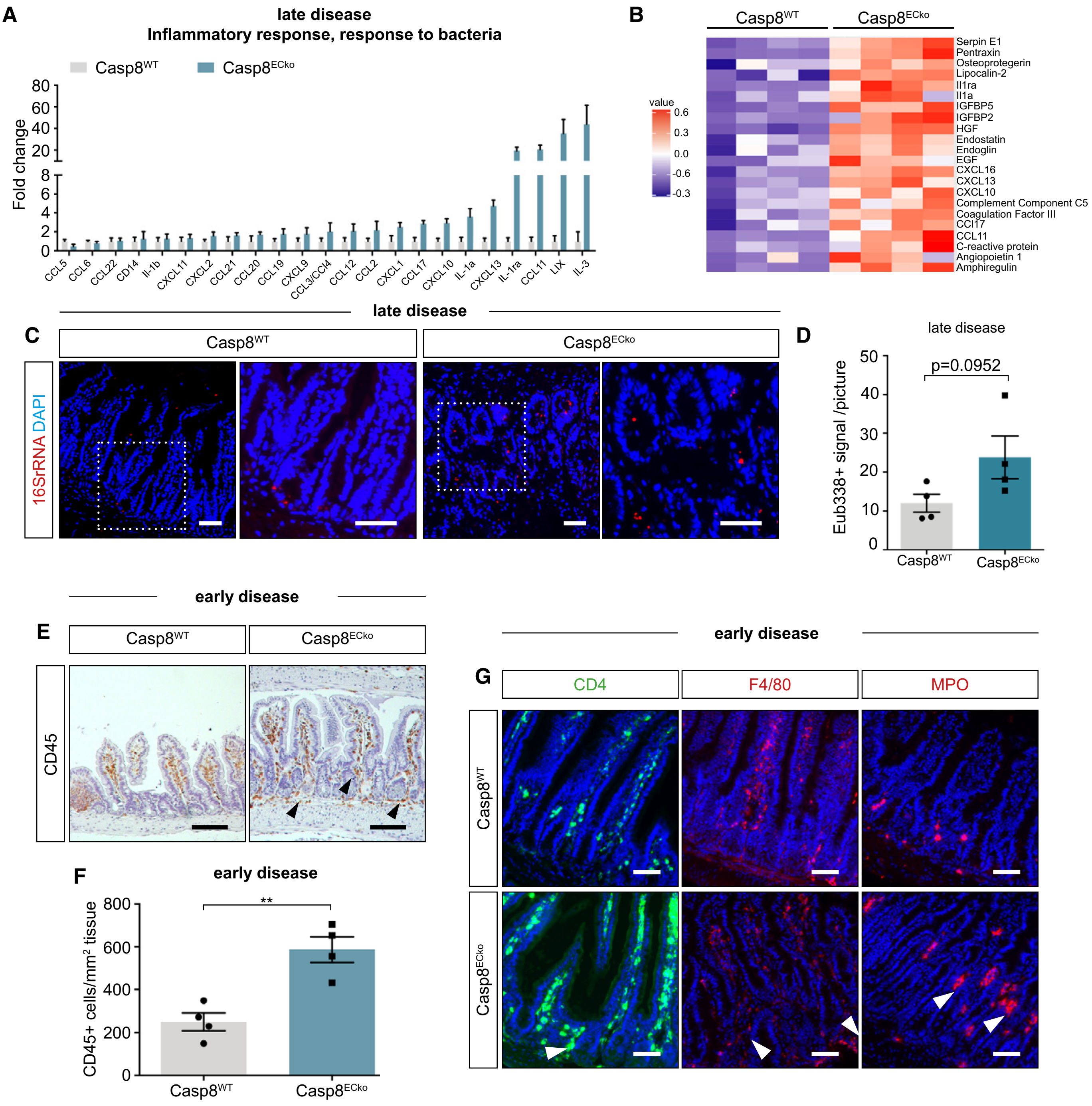
A
late disease
Inflammatory response, response to bacteria
Casp8WT
Casp8ECko
Fold change
CCL5
CCL6
CCL22
CD14
Il-1b
CXCL11
CXCL2
CCL21
CCL20
CCL19
CXCL9
CCL3/CCl4
CCL12
CCL2
CXCL1
CCL17
CXCL10
IL-1a
CXCL13
IL-1ra
CCL11
LIX
IL-3
B
Casp8WT
Casp8ECko
value
Serpin E1
Pentraxin
Osteoprotegerin
Lipocalin-2
Il1ra
Il1a
IGFBP5
IGFBP2
HGF
Endostatin
Endoglin
EGF
CXCL16
CXCL13
CXCL10
Complement Component C5
Coagulation Factor III
CCl17
CCL11
C-reactive protein
Angiopoietin 1
Amphiregulin
late disease
C
Casp8WT
Casp8ECko
16SrRNA DAPI
D
late disease
p=0.0952
Eub338+ signal /picture
Casp8WT
Casp8ECko
early disease
E
Casp8WT
Casp8ECko
CD45
early disease
G
CD4
F4/80
MPO
Casp8WT
Casp8ECko
F
early disease
**
CD45+ cells/mm² tissue
Casp8WT
Casp8ECko

Figure EV2.

◀ **Figure EV2. Loss of Casp8 in ECs leads to small bowel inflammation.**

A Graph showing increased expression of proteins associated to the GOs inflammatory response and bacterial response in small intestinal samples of $Casp8^{ECko}$ mice (n = 4 WT, 4 ECko), identified via a proteome profiler analysis.

B Row-normalized heat map of significantly upregulated genes in the proteome profiler analysis of small intestinal samples of $Casp8^{ECko}$ compared to $Casp8^{WT}$ mice (n = 4 WT, 4 ECko, unpaired students t-test, unadjusted P-values).

C, D Representative pictures (C) and quantification (D) of bacterial 16S rRNA in $Casp8^{WT}$ and $Casp8^{ECko}$ mice by *FISH* at a late disease stage (n = 4 WT, 4 ECko two-tailed unpaired student t-test). Scale bars: 50 µm.

E, F Representative pictures (E) and quantification (F) of $CD45^+$ cells in deep layers of the lamina in the small intestine of $Casp8^{WT}$ and $Casp8^{ECko}$ mice (n = 4 WT, 4 ECko, two-tailed unpaired student t-test. Black arrowheads point to accumulated $CD45^+$ cells). Scale bars: 50 µm.

G Representative pictures of T-cells ($CD4^+$), macrophages ($F4/80^+$), and granulocytes (MPO^+) in the small intestine of $Casp8^{WT}$ and $Casp8^{ECko}$ mice, showing accumulation of these cells in the crypt area (white arrowheads) in $Casp8^{ECko}$ mice already at early disease stages. Scale bars: 50 µm.

Data information: All data is shown as mean ± SEM. $^{**}P < 0.01$, ns: not significant.
Source data are available online for this figure.

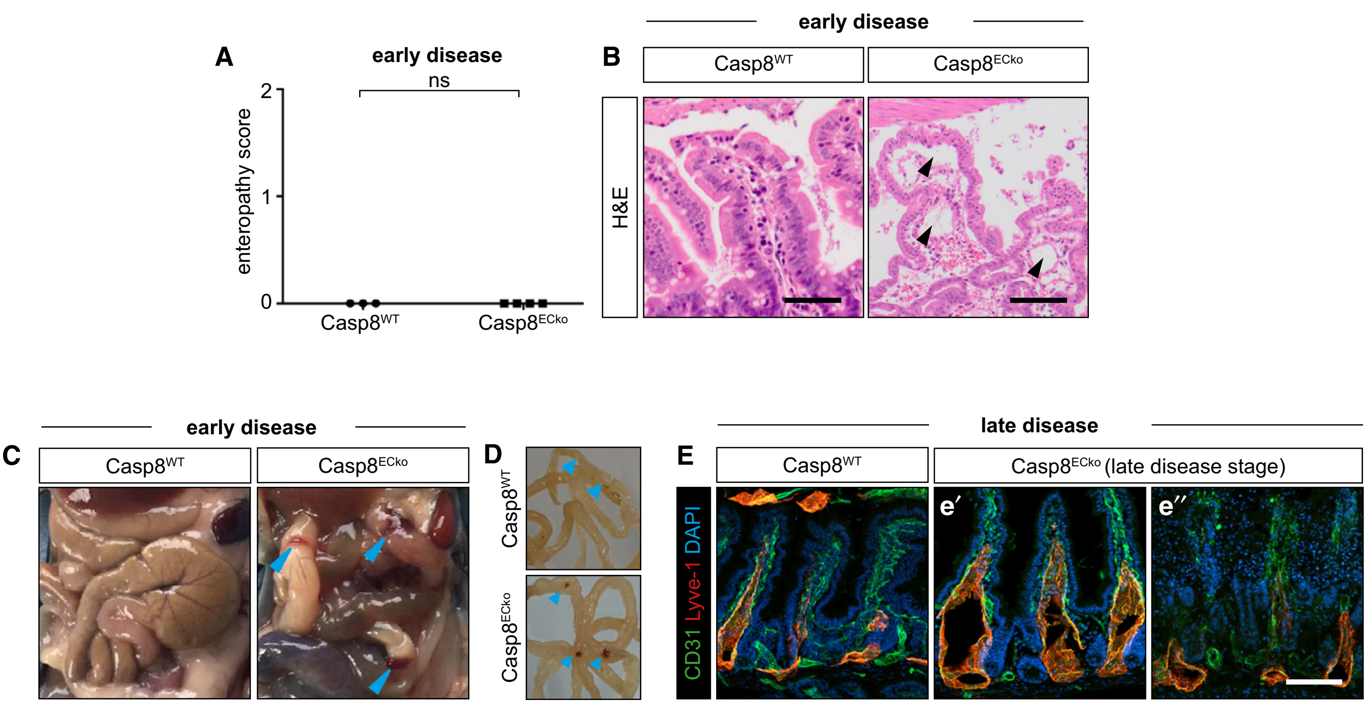

Figure EV3. $Casp8^{ECko}$ mice present increasing vascular disintegration, including edema and loss of tissue architecture in the course of disease progression.

A Quantification of intestinal pathology at an early disease stage (enteropathy score; n = 3 WT, 4 ECko, two-tailed unpaired student t-test).

B Representative pictures of the small intestinal villi of $Casp8^{WT}$ and $Casp8^{ECko}$ mice at an early disease stage (black arrow heads point to edema in $Casp8^{ECko}$ mice). Scale bars: 100 µm.

C, D Representative pictures of the small intestine of $Casp8^{WT}$ and $Casp8^{ECko}$ mice at an early disease stage (blue arrow heads point to Peyer's Patches in $Casp8^{ECko}$ mice). Cleaned intestines are shown in D for better visualization of Peyer's Patches (blue arrowheads).

E Representative images of stainings for CD31, Lyve1, and DAPI in small intestinal sections of $Casp8^{WT}$ and $Casp8^{ECko}$ mice at a late disease stage. Lacteals in $Casp8^{ECko}$ mice are severely dilated in villi that are still present (e′). Most of the villi are blunted, showing severe tissue disintegrity (e″). Scale bars: 100 µm.

Data information: All data is shown as mean ± SEM. ns: not significant.
Source data are available online for this figure.

 © 2022 The Authors

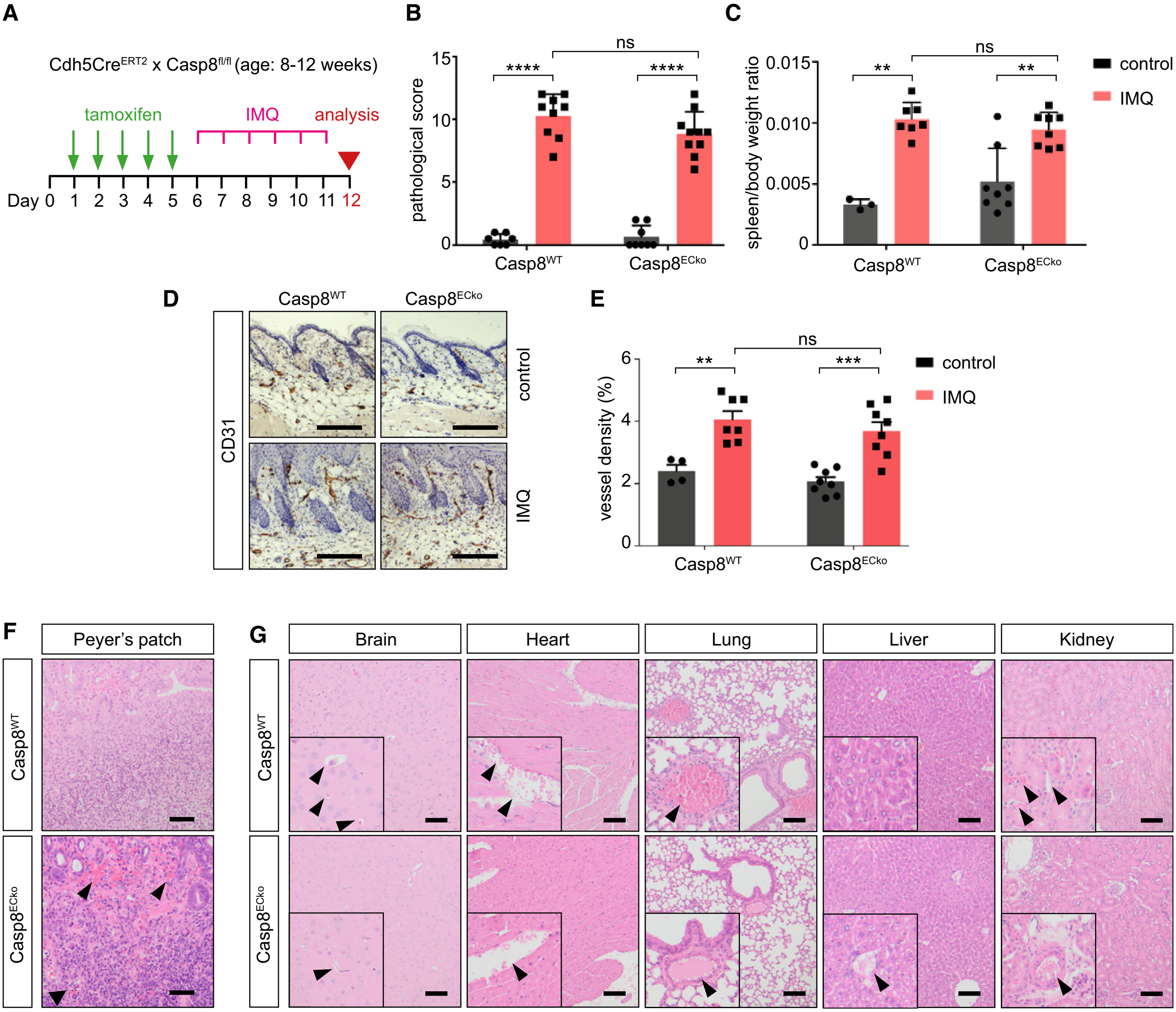

Figure EV4. Inflammation alone is not sufficient to induce vascular defects and pathology in other organs in Casp8^{ECko} mice.

A Schematic representation of imiquimod (IMQ) treatment on the back skin of Casp8^{WT} and Casp8^{ECko} mice.

B, C Quantification of skin pathology (B) and spleen weight (C) in Casp8^{WT} and Casp8^{ECko} mice with and without IMQ treatment (n = 4–7 WT control, 7–9 WT IMQ, 8 ECko control, 8–10 ECko IMQ; two-way ANOVA with Sidak's multiple comparison).

D Representative pictures of CD31 and hematoxylin staining on skin cross sections of Casp8^{WT} and Casp8^{ECko} mice with and without IMQ treatment. Scale bars: 100 μm.

E Graph showing quantification of CD31^{+} vessel area upon IMQ treatment in Casp8^{WT} and Casp8^{ECko} mice (n = 4 WT control, 7 WT IMQ, 8 ECko control, 8 ECko IMQ; two-way ANOVA with Sidak's multiple comparison).

F Representative images of H&E staining of Peyer's Patches from TNF-α treated Casp8^{WT} and Casp8^{ECko} mice. Black arrowheads point to hemorrhages (n = 5 WT, 5 ECko). Scale bars: 50 μm.

G Representative images of H&E staining of the indicated organs in Casp8^{WT} and Casp8^{ECko} mice 24 h after TNF-α injection (n = 5 WT, 5 ECko). Black arrowheads point to erythrocytes inside of (healthy) blood vessels. Scale bars: 100 μm.

Data information: All data is shown as mean ± SEM. **P < 0.01 ***P < 0.001, ****P < 0.0001; ns: not significant.
Source data are available online for this figure.

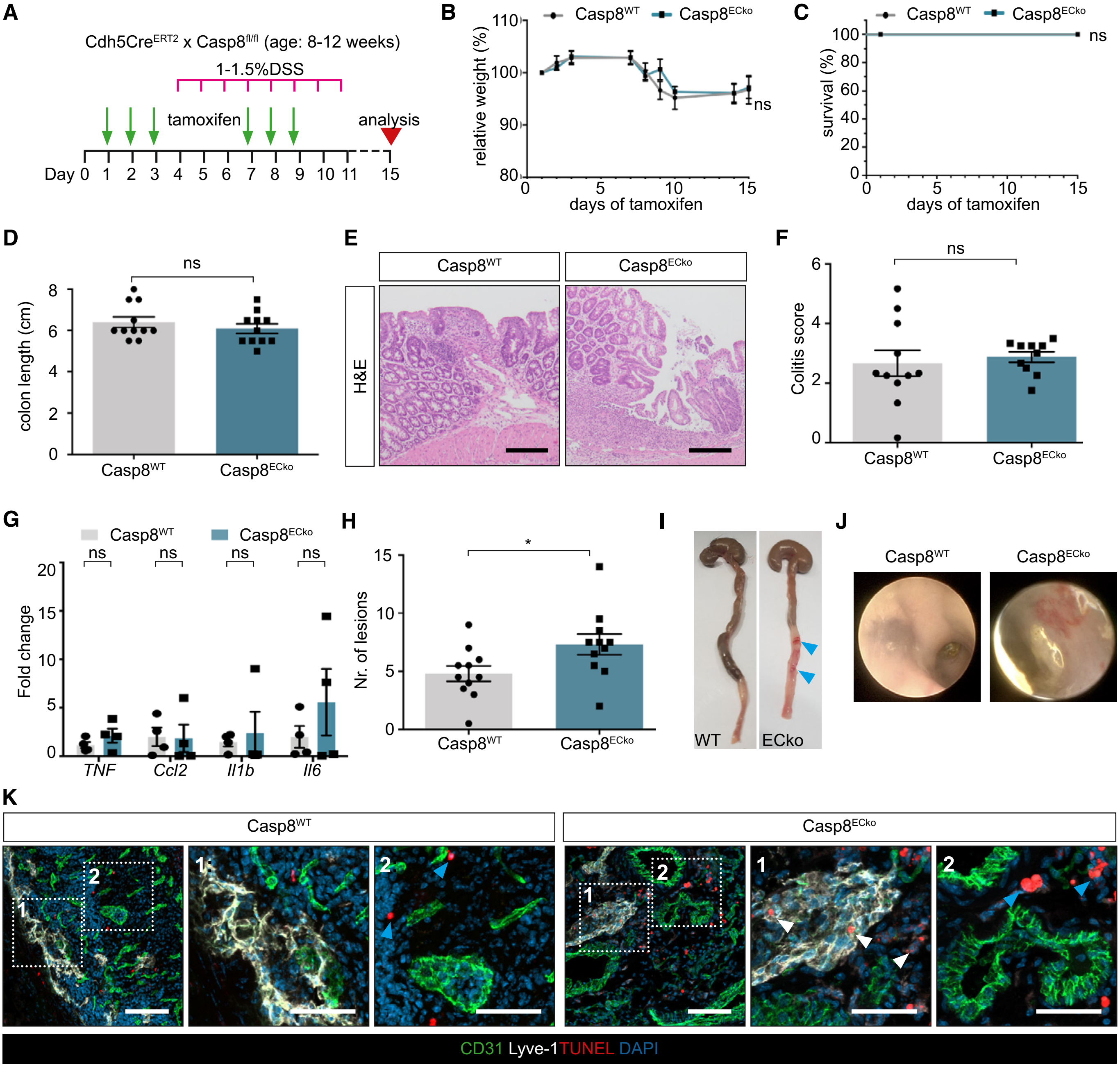

Figure EV5. Casp8^{ECko} mice are more susceptible to colitis development upon DSS treatment.

A Schematic representation of low dosage DSS treatment via the drinking water in Casp8^{WT} and Casp8^{ECko} mice.

B, C Graphs showing weight loss (B) and lethality (C) in Casp8^{WT} and Casp8^{ECko} mice (n = 11 WT, 11 ECko; B: curve comparison; C: Log-Rank test).

D Colon length of Casp8^{WT} and Casp8^{ECko} mice after tamoxifen treatment (n = 11 WT, 11 ECko; two-tailed unpaired Student's t-test).

E, F Representative images (E) and histopathological evaluation (F) of H&E staining of the large intestine of Casp8^{WT} and Casp8^{ECko} mice (n = 11 WT, 10 ECKo; unpaired t-test with Welch's correction). Scale bars: 100 μm.

G QPCR analysis of pro-inflammatory cytokines in Casp8^{WT} and Casp8^{ECko} mice upon DSS treatment (n = 4 WT, 4 ECko; multiple t-tests with Holm–Sidak method).

H Quantification of the total number of inflammatory lesions per section in the colon of Casp8^{ECko} compared to Casp8^{WT} mice upon DSS treatment (n = 11WT, 11ECko, unpaired Student's t-test).

I, J Representative pictures of large intestines (I) and colon endoscopy (J) of Casp8^{WT} mice and Casp8^{ECko} mice after DSS treatment. Blue arrowheads point to hemorrhages.

K Representative pictures of TUNEL staining together with CD31 and Lyve-1 staining shows TUNEL^{+} LECs, not BECs in Casp8^{ECko}, but not Casp8^{WT} mice upon DSS treatment. Blue arrow heads point TUNEL^{+} cells outside the vessels. White arrow heads point to TUNEL^{+} cells inside Lyve-1^{+} lymphatics. Scale bars: 50 μm, insets 25 μm.

Data information: Data shown as mean ± SEM. $^{*}P < 0.05$; ns: not significant.
Source data are available online for this figure.

 © 2022 The Authors
