# Supplementary material for: Caspase‐8 in endothelial cells maintains gut homeostasis and prevents small bowel inflammation in mice
Source: EMBO Mol Med. 2022 May 2;14(6):e14121. doi: 10.15252/emmm.202114121 (PMC9174885; doi:10.15252/emmm.202114121)
Supplement: Supplementary file 13 [file EMMM-14-e14121-s008.pdf]

EMBO Molecular Medicine

Caspase-8 in endothelial cells maintains gut homeostasis and prevents small bowel inflammation in mice

Nathalie Tisch, Carolin Mogler, Ana Stojanovic, Robert Luck, Emilia Korhonen, Alexander Ellerkmann, Heike Adler, Mahak Singhal, Géza Schermann, Lena Erkert, Jay Patankar, Andromachi Karakatsani, Anna-Lena Scherr, Yaron Fuchs, Adelheid Cerwenka, Stefan Wirtz, Bruno Köhler, Hellmut Augustin, Christoph Becker, Thomas Schmidt, and Carmen Ruiz de Almodóvar
DOI: 10.15252/emmm.202114121

Corresponding authors: Carmen Ruiz de Almodóvar (carmen.ruizdealmodovar@medma.uni-heidelberg.de) , Nathalie Tisch (nathalie.tisch@hest.ethz.ch)

Review Timeline:

Submission Date:	11th Feb 21
Editorial Decision:	11th Mar 21
Revision Received:	3rd Mar 22
Editorial Decision:	24th Mar 22
Revision Received:	6th Apr 22
Accepted:	8th Apr 22

Editor: Zeljko Durdevic

Transaction Report:

(Note: With the exception of the correction of typographical or spelling errors that could be a source of ambiguity, letters and reports are not edited. Depending on transfer agreements, referee reports obtained elsewhere may or may not be included in this compilation. Referee reports are anonymous unless the Referee chooses to sign their reports.)

11th Mar 2021

Dear Prof. Ruiz de Almodóvar,

Thank you for the submission of your manuscript to EMBO Molecular Medicine. We have now received feedback from the three reviewers who agreed to evaluate your manuscript. As you will see from the reports below, the referees acknowledge the interest of the study but also raise serious and partially overlapping concerns that should be addressed in a major revision. Particular attention should be given to in-depth mechanistic analysis.

Addressing the reviewers' concerns in full will be necessary for further considering the manuscript in our journal, and acceptance of the manuscript will entail a second round of review. EMBO Molecular Medicine encourages a single round of revision only and therefore, acceptance or rejection of the manuscript will depend on the completeness of your responses included in the next, final version of the manuscript. For this reason, and to save you from any frustrations in the end, I would strongly advise against returning an incomplete revision.

Considering the extent of the revision, I am happy to extend the revisions time to six months. We realize that the current situation is exceptional on the account of the COVID-19/SARS-CoV-2 pandemic. Therefore, please let us know if you need more than six months to revise the manuscript.

I look forward to receiving your revised manuscript.

Yours sincerely,

Zeljko Durdevic

Zeljko Durdevic
Editor
EMBO Molecular Medicine

***** Reviewer's comments *****

Referee #1 (Comments on Novelty/Model System for Author):

The model provides a powerful analysis of the organ-specific consequences of the lack of Caspase 8 in endothelial cells. The finding of developement of an inflammation driven pathology specific for the small instetine that is dependent on the presence of microbiota is unexpected and may have important implications for the further understanding of the development of inflammatory intestinal syndroms liek IBD and Mrobus Crohn. A Question that remains somewhat open is the relative importance of blood and lmyphatic vessels, this could be addressed by deletion of Caspase 8 in LECs using a Prox1-CreERT2 driver line.

Referee #1 (Remarks for Author):

The manuscript entitled „Caspase-8 in endothelial cells is required to maintain gut homeostasis and to prevent small bowel inflammation in mice" investigates the consequences of inducible endothelial cell (EC)-specific deletion of Caspase 8 in newborn mice.
The study reports progressive wasting that started three weeks after deletion induction and was associated with hemorrhages located in the small intestine. Early lesions in still asymptomatic animals were detected two weeks after tamoxifen treatment including structural vascular changes, increased blood vessel permeability and bleeding in the Peyer's Patches. This organ malfunction progressed to a manifest enteritis with loss of gut vascular barrier integrity, inflammation, tissue erosion and necrosis affecting selectively the small intestine. Disease development was associated with expression of a proinflammatory gene signature and EC activation, but also reactive activation of tissue healing pathways. Induction of Caspase 8-deletion on a

MLKL-negative background did not elicit the pathology, providing strong evidence for a central involvement of the necroptosis pathway. Furthermore, in a psoriasis skin model inflammation in combination EC-specific Caspase 8 deletion was not sufficient to cause vascular hemorrhages and disease development similar to the intestine, suggesting the presence of organotypic factors. The intestinal microbiome was identified as one such factor, because ablation of the gut microbiota by antibiotic treatment protected EC Caspase 8-deleted mice from the development of small intestinal lesions.

Major points
The study by Tisch and colleagues presents convincing evidence that EC-specific Caspase 8 deletion may act as driver of pathological intestinal inflammation in a process that is dependent on the TNFa/RIPK/MLKL mediated necroptosis pathway. Intestinal microbiota are identified an important organotypic factor underlying the organ-specific development of this pathology. The study significantly broadens our knowledge about the role of the intestinal vasculature in the maintenance of tissue integrity. It provides valuable information for the understanding of the potential role of the vasculature in the development of IBD. While this part of the manuscript is clearly presented and fully supported by the data, several important questions concerning the mechanistic analysis, in particular the assessment of relative contribution of blood (BECs) and lymphatic endothelium (LECs) remain open.
The manuscript focuses on an organ-specific defect resulting from EC-specific Caspase 8 deletion that selectively manifests in the intestinal vasculature. Yet for mechanistic analysis, mRNAs expression of necroptosis pathway components, TNFa regulation under caspase inhibition and cell death after TNFa stimulation were all measured in human dermal blood and lymphatic endothelial cells. While establishment of primary intestinal EC cultures may presently not be feasible, the suitability of the used EC culture systems should be discussed. Furthermore, expression analysis of essential necroptosis pathway components like TNFR1 and RIPK3 could be performed on freshly isolated mouse intestinal BECs and LECs.
Functional experiments using the Caspase inhibitor ZIETD in combination with TNFa and LCL161 identified LECs as most susceptible to necroptosis. Is this reflected in the Tunel data from intestinal samples when BECs and LECS are separately considered (CD31 vs Lyve1 staining)?
Upon disease onset, PPs are affected and develop the first hemorrhages, this deserves a more in depth analysis, possibly with additional intermediate time points. Are the endothelial defects preceded by infiltration of inflammatory cells, or is endothelial activation starting the cascade? The course of events during disease development in the Peyer's patches can be described in more detail. Is endothelial necrosis also prominently observed there as well? If so are BECs, LECs or both populations equally affected? An important issue to address is how at the early stage of disease development, before the break down of the epithelial barrier, gut specific information about the microbiota is conveyed to and interepreted in Peyer's patches? Are antigen presenting immune cell populations responsible for the transfer or is there a constant basal penetration of the epithelial barrier? An elegant way to distinguish the relative contribution of BECs and LECS would be the deletion of Casp8 specifically in LECs using the Prox1-CreERT2 deleter strain.
Specific points
Fig. S2 B) Conversion of the Rosa-mTmG strain is often used as a proxy for deletion by a given deleter allele in a particular cell type. However, this should be considered with caution as Rosa mTmG conversion is no guarantee for successful deletion of other in particular multiple floxed alleles.
Fig. S2 C,D) Addition of qRT-PCR data is most commendable, however, inclusion of intestinal ECs would have been desirable.
Fig. S2 F) Staining for mG in the Lyve1 positive areas (LECs) appears weaker compared to the surrounding BECs. Why is that, given that the same reporter allele is activated in both cell types?
Legend to Fig. 3 (K) and (J) are mixed up, (K) should read (J) and describes Caspase 8 inhibition not deletion. In addition (K) should be inserted before the sentence starting: ..."Quantification of cell death...".
Fig. 2 and following Figs.: Time of analysis (early or late after Tamoxifen induction) could be indicated in the figures for easy reference as done in Fig.1.

Referee #2 (Comments on Novelty/Model System for Author):

Several aspects of the analysis need to be further improved. These are outlined in the "Remarks to be sent to the authors".

Referee #2 (Remarks for Author):

Tisch et al. describe how endothelial-specific Casp8 deletion results in small intestinal enteropathy associated with inflammation and increased cell death, ultimately leading to increased lethality. This pathology is present only in the small intestine, although Casp8 is targeted in endothelial cells of all other organs. The authors further show that this phenotype is ameliorated either by deletion of MLKL or antibiotic treatment after weaning, indicating the crucial role of necroptosis and microbial responses in its aetiopathogenesis. This is an interesting paper that reveals an important homeostatic role for endothelial Casp8 and endothelial cells in general in the intestine. However, the molecular mechanisms underlying the phenotype described are not analyzed in-depth, while several aspects of the analysis need to be further improved to support the conclusions drawn.

Major Comments:
1) The authors have performed several experiments to analyze the phenotype of increased lethality and enteropathy that the

Casp8ECko mice display. However, there are several concerns/questions that need to be addressed:
- What is the extent of enteropathy in the small intestine? Can the authors provide low and high magnification images to better show this pathology? Why do control Casp8wt mice in Fig. 1H have a positive enteropathy score that only doubles in the Casp8ECko mice? Can the authors show in supplementary figures the different parameters of the histopathological scores in control and experimental mice? In Figure 1I how is the number of mucosal hemorrhages calculated (per mouse, per field, other)?
- In Fig. 2G and 1G (g') the intestine seems to have lost entirely its structure. This is not obvious in other images (e.g. Fig. 2E, 2H). Which are more representative of the phenotype? Could images in 1G and 2G be the result of organ/organism death?
- The authors characterize the phenotype as having "features of chronic intestinal inflammation (reminiscent of IBD in humans)". It would be preferable for the interpretation of results if inflammatory cell infiltration is analyzed and quantified by FACS (including a more detailed analysis of immune cell populations).
- In Fig. 3A, it is not obvious what the authors refer to as tissue edema. Is it the relative height of intestinal epithelial cells? Have the authors measured this? What is the potential relationship with the phenotype observed? Also, villi from Casp8ECko seem more elongated, which could be related to the part of the intestine that they come from. Villi from the same area of the small intestine should be shown. Are these images maximum projections of well-oriented villi? Since at this stage (Day 15), hemorrhages were mainly present in the PPs, imaging of the vasculature of PPs and analysis of immune cells there could be more meaningful.
- In Figure 3H, it is not clear what the authors refer to as stroma. Is it non-epithelial, non-endothelial cells? How do the authors explain increased Tunel+ cells in the epithelium and stroma? Could cell death be secondary to ensuing organ death rather than aetiopathogenic?
- In my opinion, the MLKLko data are the most supportive of a role for cell death/necroptosis in the phenotype and should be included in the main Figures.
- Based on the in vitro data that show an increase in LECs' cell death after simultaneous inhibition of Casp8 and stimulation with TNF and LCL161, the authors suggest that LECs have a more important contribution in the disease development. Are there any in vivo data to support this conclusion, e.g. increased cell death in LECs? Experiments with tamoxifen-induced primary cells would be more representative of the in vivo data.

2) PPs should be analyzed in more detail in the TNF model to support common mechanisms with the spontaneous phenotype of Casp8ECko mice. High and low magnification images, analysis of the vasculature, Tunel assay for the identification of endothelial cell death, immune cell analysis should be performed. It would also be interesting to see if MLKL deletion rescues this phenotype too.

3) The DSS experiments presented in Fig. 5 require additional analysis to be able to support the conclusions. In more detail:
- What is the clinical score of these mice? Do they show increased blood in their stool as a result of hemorrhage? Do they have decreased hematocrit? These data would help define the pathophysiological significance of the findings.
- The authors use a low dose of DSS. What happens when they give a more standard dose of 2-2.5%? Is there any difference in survival and/or clinical/histopathological scoring?
- Representative H&E stainings should be shown to better assess histologically the differences in the phenotype.
- Do blood/lymphatic vessels show increased cell death in this model? Is there an increased expression of cytokines, chemokines, etc? Is there a difference in inflammation and inflammatory cell infiltration?
- This set of experiments is useful to show increased susceptibility to chemically-induced colitis, but the data presented cannot support the conclusion that bacterial challenge in these mice disrupts the GVB and leads to intestinal disease and inflammation.

4) The experiments showing the dependency of the phenotype on the presence of microbiota are very interesting. However, they are largely underexplored and there is no proposed mechanism. For example, how does the microbiota affect GVB? Is there an increased influx of bacteria in the tissue potentially due to an altered epithelial barrier? Is this process TNF dependent as the authors hypothesize?

Minor Comments:
- For the short title, I would suggest: "Endothelial Casp8 prevents intestinal inflammation".
- More information is needed in the Methods sections. For techniques that are referenced elsewhere, a brief explanation would be helpful. Other specific information that is missing: primers and qRTPCR conditions for analysis of 16S bacterial rDNA, a vendor for recombinant mouse TNF, qRTPCR primers, and conditions for Fig. 2A-B.
- The number of mice in many in vivo experiments is quite small (3-4 mice). Have these experiments been repeated?
- p.11: "restricted" seems more appropriate than "specific".
- Can the authors also show the levels of Casp8 deletion in small intestinal and colonic endothelial cells to exclude potential organ-specific differences that could explain the absence of a phenotype in the colon? Similarly, they should also show deletion efficiency in the skin.
- Colocalization of CD31/IsoB4 and GFP is not evident in the images provided in Fig. S2B. Split channels and higher magnification would help. A description of how the quantification was performed should also be added in the Methods.
- In the manuscript text, it is indicated that Fig. 2C, D contains gene expression data, but in the figure legend, they are described as the results of the proteome profiler analysis. Which is correct?
- Unaltered vessel permeability in the brain is stated in the text (p. 13), but not shown in any figure.
- In Fig. S3A, the label is missing.

- Fig. S6 should be explained in more detail in the text and more information about the analysis should be provided in the Methods and the Figure Legend. Can the authors identify clusters co-expressing the indicated genes?
- Can the authors explain why they use a different tamoxifen protocol in the psoriasis model?
- Reference to Fig. 5A-D is not correct in the text. Reference of Fig. S5E-F in p.17 should be changed to Fig. 5E-F.
- In many cases, analyses of the two different time-points are interchangeable, making it difficult to follow the paper and make comparisons. An indication of the time-point of analysis in each panel would be helpful.

Referee #3 (Remarks for Author):

In the present manuscript, Tisch and colleagues investigated the function of Caspase-8 in endothelial cells (EC). For this purpose, they generated tamoxifen inducible EC specific Caspase-8 knockout mice using Cdh5(PAC)-CreERT2. Casp-8ECko mice succumbed to death, about 3 weeks after the first tamoxifen injection. Casp-8 deletion was associated with small bowel inflammation, vascular dysfunction and hemorrhage in the small intestine, whereas all others organs remained unaffected. Furthermore, they provide evidence that this specific effect was TNF-R1-dependent, leading to cell death in form of necroptosis, as the additional constitutive deletion of MLKL, could completely reverse the phenotype. Moreover, they showed that Inflammation alone was not sufficient to induce vascular defects and pathology in Casp8ECko mice, since other organs (skin or liver) were not differently affected in a psoriasis inflammation model or the systemic TNF-dependent SIRS model. Finally, they could show that the intestinal microbiota is required to trigger the disease development in a gut-specific manner.
Overall the study is well conducted, the data clearly presented and the conclusions are mainly justified. However, the following issues should be taken into account.
The involvement of TNF in spontaneously induced pathogenesis remains to be experimentally proven. Since an additional sub-crossing with TNF-R1 deficient mice is certainly time consuming, treatment of the Casp-8ECko mice with Etanercept could alternatively be performed in order to investigate the involvement of TNF in the spontaneous phenotype.
Moreover, a previous work from the group of Manolis Pasparakis (Schwarzer et al. 2020) suggested that next to TNF-R1 also ZBP1 could be involved in driving intestinal inflammation in human patients with mutations in the CASP8 gene, who were reported to develop severe forms of very early onset IBD that was refractory to anti-TNF treatment (Lehle et al., 2019). Do the authors have any evidence that ZBP1 is also relevant in their model?
Minor comments:
1. Do Casp8ECko/MLKLko mice develop a later phenotype?
2. Figure 2E-F: A counting of PCNA positive cells would be more useful than QPCR analysis to Ki-67, alternatively staining with statistical analysis. In addition, a zoom-in would be appropriate, the image is too small in order to see specific differences.
3. Which antibiotics were used. Are species-related differences to be expected
4. In Figure 4E, the liver and kidney seems to be affected upon TNF treatment in WT but not in Casp8ECko mice. Do the authors have additional parameters regarding liver damage (e.g. serum transaminases, AST/ALT) or kidney damage (creatine kinase (CK))? In general, the images are too small. A zoom-in would be helpful.
5. The order should be adjusted. In the text, however, 5G is described first, then 5D-F. Please adjust

We thank all reviewers for their insightful and constructive criticisms that have guided us in the production of a thoroughly revised version that we hereby resubmit. The revision entailed the inclusion of two new main Figures, several new panels in the old figures, four new Supplementary Figures and the selection of five expanded view figures. We hope that the revised version of the manuscript now meets the reviewer's expectations.

Referee #1

(Comments on Novelty/Model System for Author)

The model provides a powerful analysis of the organ-specific consequences of the lack of Caspase 8 in endothelial cells. The finding of development of an inflammation driven pathology specific for the small instetine that is dependent on the presence of microbiota is unexpected and may have important implications for the further understanding of the development of inflammatory intestinal syndroms liek IBD and Morbus Crohn. A Question that remains somewhat open is the relative importance of blood and lymphatic vessels, this could be addressed by deletion of Caspase 8 in LECs using a Prox1-CreERT2 driver line.

(Remarks for Author)

The manuscript entitled „Caspase-8 in endothelial cells is required to maintain gut homeostasis and to prevent small bowel inflammation in mice" investigates the consequences of inducible endothelial cell (EC)-specific deletion of Caspase 8 in newborn mice.
The study reports progressive wasting that started three weeks after deletion induction and was associated with hemorrhages located in the small intestine. Early lesions in still asymptomatic animals were detected two weeks after tamoxifen treatment including structural vascular changes, increased blood vessel permeability and bleeding in the Peyer's Patches. This organ malfunction progressed to a manifest enteritis with loss of gut vascular barrier integrity, inflammation, tissue erosion and necrosis affecting selectively the small intestine. Disease development was associated with expression of a proinflammatory gene signature and EC activation, but also reactive activation of tissue healing pathways. Induction of Caspase 8-deletion on a MLKL-negative background did not elicit the pathology, providing strong evidence for a central involvement of the necroptosis pathway. Furthermore, in a psoriasis skin model inflammation in combination EC-specific Caspase 8 deletion was not sufficient to cause vascular hemorrhages and disease development similar to the intestine, suggesting the presence of organotypic factors. The intestinal microbiome was identified as one such factor, because ablation of the gut microbiota by antibiotic treatment protected EC Caspase 8-deleted mice from the development of small intestinal lesions.

Major points

1. The study by Tisch and colleagues presents convincing evidence that EC-specific Caspase 8 deletion may act as driver of pathological intestinal inflammation in a process that is dependent on the TNFa/RIPK/MLKL mediated necroptosis pathway. Intestinal microbiota are identified an important organotypic factor underlying the organ-specific development of this pathology. The study significantly broadens our knowledge about the role of the intestinal vasculature in the maintenance of tissue integrity. It provides valuable information for the understanding of the potential role of the vasculature in the development of IBD. While this part of the manuscript is clearly presented and fully supported by the data, several important questions concerning the mechanistic analysis, in particular the assessment of relative contribution of blood (BECs) and lymphatic endothelium (LECs) remain open.

Answer: *We thank the referee for his/her comment. We have addressed this major point during the revision of our manuscript, and we provide the newly generated data in the revised version. First, we generated PDGFb-CreERT2 x Casp8$^{fl/fl}$ mice for blood endothelial cell (BEC) specific Casp8 deletion (see our answer to this reviewer's comment #6). Second, we complemented our in vivo cell death analysis, where we quantified the number of TUNEL+ cells in the endothelium by differentially studying cell death in BECs and LECs and specifically analyzed cell death in the Peyer's Patches (and their vasculature) now (see the answer to this reviewer's comment #4). Overall, the newly generated data support our previous model and brings further understanding on the relative contribution of the blood and lymphatic vasculature in maintaining intestinal homeostasis.*

2. The manuscript focuses on an organ-specific defect resulting from EC-specific Caspase 8 deletion that selectively manifests in the intestinal vasculature. Yet for mechanistic analysis, mRNAs expression of necroptosis pathway components, TNFa regulation under caspase inhibition and cell death after TNFa stimulation were all measured in human dermal blood and lymphatic endothelial cells. While establishment of primary intestinal EC cultures may presently not be feasible, the suitability of the used EC culture systems should be discussed. Furthermore, expression analysis of essential necroptosis pathway components like TNFR1 and RIPK3 could be performed on freshly isolated mouse intestinal BECs and LECs.

Answer: *As the referee already indicates, up to date mouse intestinal EC cultures are not feasible and have not been successfully established by us and others (Hagerling, Hoppe et al., 2018, Spadoni, Zagato et al., 2015). Therefore, it is currently common that ECs from other organs, e.g. lung (Spadoni et al., 2015) or skin (Hagerling et al., 2018) are used to complement the in vivo analysis of the intestinal vasculature. Also, it is uncertain to which extend isolated primary ECs maintain their organotypic features after isolation, or even in culture, and there are already hints from the literature, that intestinal LECs quickly lose their characteristic properties after isolation (Hagerling et al., 2018). As culture of intestinal BECs and LECs has not been feasible, we decided to sort intestinal BECs and LECs and performed qPCR analysis of the isolated cells for essential necroptosis pathway components, as suggested. Interestingly, qPCR analysis of sorted intestinal BECs and LECs by flow cytometry did not show the differences that we have previously seen in HDMECs and HDLECs (Fig. R1, below), suggesting that indeed molecular differences exist between either organ specific ECs (skin vs. intestine), human vs. mouse ECs, or in vitro cultured vs. freshly isolated ECs. Due to this new result we decided to remove the data from the human dermal BECs and LECs from our manuscript, as we believe that those cells might not be a good model for our analysis. Because of those limitations we decided to undertake an in vivo approach and analyze cell death of lymphatic vs. blood endothelium in the small intestine of Casp8^{WT} and Casp8^{ECKO} (new Fig. 5A and answer to the next comment of this reviewer).*

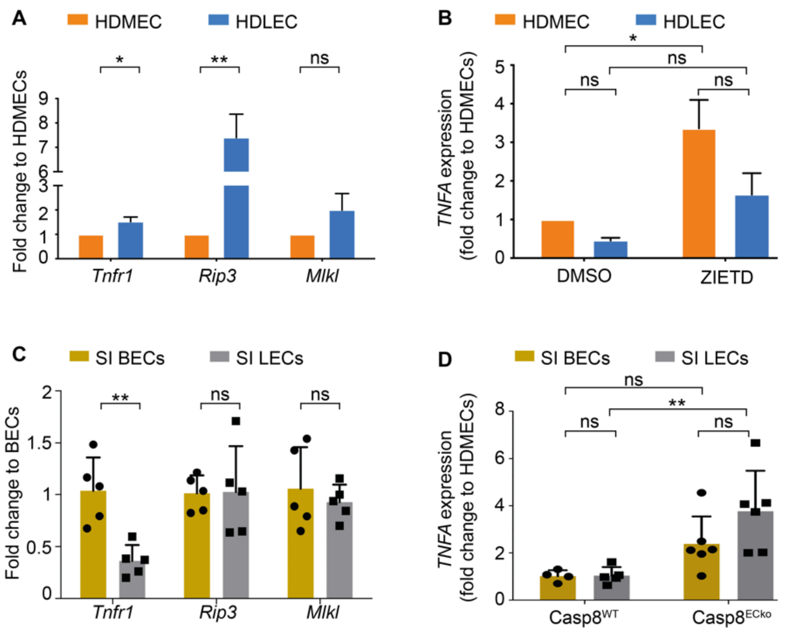

Fig. R1: Comparison of cell death signaling molecules between HDMECs/HDLECS and primary small intestine (SI) BECs and LECs. (**A**) Expression of *Tnfr1*, *Rip3*, *Mlkl* in HDMEC and HDLEC (human skin, previous Fig.3I). (**B**) Expression of *TNFA* in HDMEC/HDLEC after Casp8 inhibition (previous Fig.3J). (**C**) Expression of *Tnfr1*, *Rip3*, *Mlkl* in primary small intestine (SI) BECs and LECs. (**D**) Expression of *TNFA in BECs and LECs* of Casp8^{WT} and Casp8^{ECko} small intestine. Data shown as mean ± SEM. * P <0.05, ** P <0.01, ns: not significant.

3. Functional experiments using the Caspase inhibitor ZIETD in combination with TNF-*α* and LCL161 identified LECs as most susceptible to necroptosis. Is this reflected in the Tunel data from intestinal samples when BECs and LECS are separately considered (CD31 vs Lyve1 staining)?

Answer: *As indicated above, it was not feasible to isolate and culture intestinal BECs and LECs to perform those experiments. Thus, in addition to our previous quantitative analysis of CD31/TUNEL+ cells in small intestinal villi (old Fig.3 G, H and Fig. S4 D, E now Fig.4 A,B and Appendix Fig. S2), in the revised version of our manuscript, we now focused on specifically analyzing cell death in intestinal BECs and LECs in vivo. In the new Fig. 5A we now provide TUNEL staining together with CD31 and Lyve-1 staining to determine cell death in BECs and LECs respectively. We focused this analysis on Peyer's Patches at an early disease timepoint, as this is where we observed the first hemorrhages. We hereby distinguished between non-hemorrhagic and hemorrhagic Peyer's Patches to also get a better timely resolution of the disease events (see this referees comment #4). This qualitative analysis shows that indeed, TUNEL+ ECs are localized to the lymphatic vasculature (new Fig. 5A), while BECs are not TUNEL+ at those time points and thus suggests that intestinal LECs are more susceptible to cell death than BECs.*

4. Upon disease onset, PPs are affected and develop the first hemorrhages, this deserves a more in depth analysis, possibly with additional intermediate time points. Are the endothelial defects preceded by infiltration of inflammatory cells, or is endothelial activation starting the cascade? The course of events during disease development in the Peyer's Patches can be described in more detail. Is endothelial necrosis also prominently observed there as well? If so are BECs, LECs or both populations equally affected?

Answer: *We thank the reviewer for this suggestion. In the revised version of our manuscript, we provide a detailed analysis of the vasculature, including TUNEL staining (see above), in Peyer's Patches. At 15 days after the first tamoxifen treatment (early disease stage), not all Peyer's Patches are equally affected, with some already showing hemorrhages and some*

others not presenting any macroscopic vascular phenotype (new Fig. 3A). Hence, we dissected and differentially analyzed Peyer's Patches with and without hemorrhages, hypothesizing that those without hemorrhages would represent an early step of disease development, potentially before EC death has occurred. This would allow us to track the development of the vascular defects and disease in more detail, from non- hemorrhagic, to mild and severely hemorrhagic (diseased) stages.

First, we analyzed the blood and lymphatic vasculature in wholemount stainings of Peyer's patches (new Fig.3 B, C). Lymphatic vessel density was analyzed based on Lyve-1 staining and showed that a reduction of lymphatic vessel density was an early event during disease development, even before hemorrhages had occurred. Unfortunately, the lack of a good blood vessel specific marker in Peyer's Patches (CD31 also labels lymphatics), and strong tissue autofluorescence (potentially from immune cells) precluded the quantitative analysis of blood vessel density. However, morphological differences in the blood endothelium only became apparent after the lymphatic vessel density was already reduced (see new Fig. 3C, c''), suggesting that the lymphatic vasculature is affected earlier. This data is in line with the differential cell death analysis that we are now providing – showing TUNEL+ ECs in lymphatic, but not blood endothelial vasculature (new Fig. 5A).

To analyze immune cell infiltration, we decided to try to identify the different immune cell populations by flow cytometry. Our approach was as follows: Peyer's Patches with and without hemorrhages at 15 days after the first tamoxifen treatment were dissected to characterize different immune cell populations (myeloid cells (neutrophils, monocyte/macrophages and dendritic cells), innate lymphocytes (NK cells, ILC1s, ILC2s and ILC3s) and T cells (γδ+ T cells and αβ+ T cells, including NKT cells, CD8+ T cells and helper CD4+ T cells – Th1/2, Th17 and Foxp3+ regulatory T cells). Expression of PD-1 and CD44 on T cell was measured as indication of T cell activation. Different subsets of MHC II-expressing antigen presenting cells, including gut-resident DCs, defined by differential expression of CD8α and CD103, and CX3CR1+ macrophages, were assessed for the expression of costimulatory molecules (CD80 and CD86).

Unfortunately, recovery of CD45+ cells from $Casp8^{ECko}$ *lamina and Peyer's Patches was very low (Fig.R2A,B), which might be in part due to immune cell death. Indeed, while death of lymphatic ECs was observed already in Peyer's Patches without or presenting small hemorrhages, death of other cells ((CD45+) TUNEL+ cells) in the Peyer's Ptches was only observed at later events, indicating that immune cell death is a secondary event (new Fig. 5A). This result, which is by itself very interesting (see note below), prevented us from further in-depth analysis.*

Of note: the observation that immune cell recovery is reduced in the Casp8 knockout mouse model is very interesting and has also been observed in other mouse models of intestinal chronic inflammation upon Casp8 deletion in the epithelium (personal communication with Christoph Becker). We believe that trying to characterize this observation lies out of the scope of this study and will be further investigated in a follow up study. We will acknowledge this limitation in our manuscript.

While we were not able to provide such a deep analysis of the immune cell populations, we already provided CD4, F4/80, MPO and NIMPR14 stainings at two different disease timepoints, together with histopathological analysis of H&E stained tissue sections (which also included scoring of inflammation and immune cell infiltration). In addition, we also provide a detailed cytokine analysis, which further supports our proposed model. This set of experiments is comparable to one of the landmark papers identifying Casp8 as key necroptosis regulator in the intestine, and Casp8 dependent small bowel inflammation in mice and humans (Gunther, Martini et al., 2011).

Altogether, we hope that this immune cell characterization will be sufficient for the first description of our newly presented mouse model in this study, where we focus on the vasculature. We will acknowledge the limitation of our study accordingly in the revised manuscript.

Still, following the main focus of our study, and as (1) we are targeting ECs in our cell type specific Casp8 knockout mouse model, (2) TUNEL+ cells first occur in the vasculature, and (3) this phenotype is rescued in Casp8^{ECko}/MLKL^{ko} mice, we believe that we provide sufficient data to show that EC dysfunction (which is the focus of our study) is preceding any change or activation in the immune system.

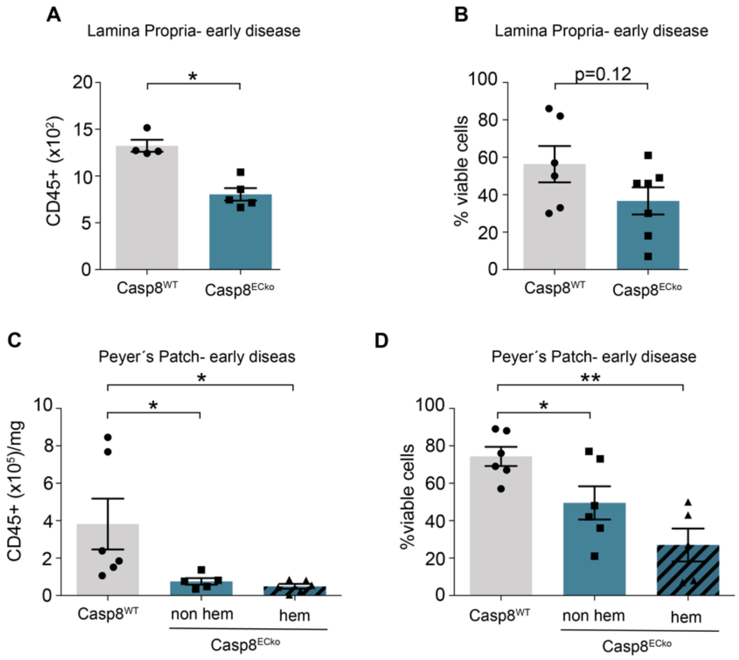

Fig. R2: Immune cell isolation in lamina propria and Peyer´s Patches of Casp8^{WT} and Casp8^{ECko} mice. (**A** and **B**) Total number of CD45+ cells (A) and cell viability (B) as measured by live-dead automated cell counting from early disease lamina propria. (**C** and **D**) Total number of CD45+ cells (C) and cell viability (D) as measured by live-dead automated cell counting from early disease Peyer´s Patches from Casp8^{WT} and non-hemorrhagic/hemorrhagic Peyer´s Patches of Casp8^{ECko} mice. Data shown as mean ± SEM. * P <0.05, ** P <0.01.

5. An important issue to address is how at the early stage of disease development, before the break down of the epithelial barrier, gut specific information about the microbiota is conveyed to and interpreted in Peyer's patches? Are antigen presenting immune cell populations responsible for the transfer or is there a constant basal penetration of the epithelial barrier?

Answer: *Several studies have described how gut specific information about microbiota is conveyed and interpreted in Peyer's Patches already under steady state conditions, in healthy animals when the epithelial barrier is intact (Morikawa, Tsujibe et al., 2016). For example, information about the intestinal microbiota is mainly transferred by active sampling of bacterial products from the intestinal lumen via specialized epithelial cells, and antigen presenting immune cells. For example, M-cells are specialized enterocytes that take up microbial products by transcytosis (e.g. phagocytosis, endocytosis, etc.) from the intestinal lumen before migrating specifically into Peyer's Patches, where those microbial products are presented to different immune cells (Kobayashi, Takahashi et al., 2019). Such a sampling and presentation of microbial material in the Peyer's Patches is also required for proper maturation of the intestinal immune system (Chung, Pamp et al., 2012). Thus, microbiota information is presented in PPs in homeostatic conditions via different cellular and molecular mechanisms, with no infiltration of microbiota.*

In line with this, we did not see a significant constant basal penetration of microbiota into the lamina propria (new Appendix Fig. S3C) in WT mice. This data is also consistent with other studies where microbiota is not detected across the epithelial barrier under steady state conditions (Gunther, Buchen et al., 2015, Swidsinski, Loening-Baucke et al., 2005). Furthermore, as depleting the microbiota with antibiotics before the initial tamoxifen treatment (and thus Casp8 deletion) fully protects the mice from disease development, this further supports the idea that steady-state microbiota-immune cell communication is an upstream regulator of Casp8 function in ECs. We therefore propose the hypothesis that microbiota information is conveyed to Peyer's Patches in Casp8^{ECko} mice in a similar way as in WT, indirectly via the immune system, without the need of bacterial translocation. It is then the signaling pathways activated in PPs that are different due to the lack of Casp8 in ECs. However, it is tempting to speculate that microbial products might also be directly presented to ECs, which requires further investigation. To address this question, we analyzed the expression of Toll-like receptors (TLRs; required to sense microbial products) in sorted intestinal BECs and L ECs (new Appendix Fig. S9). The main TLRs, TLR1,3 and 4 are all expressed in both populations.

To explore the possibility that a difference in microbiota populations between Casp8^{WT} and Casp8^{ECKO} might be a potential contributor to the phenotype, we also performed microbiome sequencing. Analysis of the sequence data at an early disease stage did not show any significant difference between Casp8^{WT} and Casp8^{ECko} mice, suggesting that microbiota differences are not preceding vascular dysfunction in Casp8^{ECko} mice (new Appendix Fig. S11A,B).

In summary, we propose a model were microbiome-induced cytokine production signals to the vasculature. Under normal conditions, when ECs express Casp8, a pro-survival mechanism is in place to control vascular homeostasis. However, in the absence of Casp8, as cytokines, such as TNFα, are potent inducers of cell death in the absence Casp8, the endothelium in Casp8^{ECko} mice becomes sensitive to necroptosis, which explains the initial appearance of hemorrhages in Peyer's Patches, home to the biggest immune cell reservoir.

6. An elegant way to distinguish the relative contribution of BECs and LECS would be the deletion of Casp8 specifically in LECs using the Prox1-CreERT2 deleter strain.

Answer: *We totally agree that a cell type specific deletion of Casp8 is the most elegant way to distinguish the relative contribution of BECs and LECs. As suggested, we generated tamoxifen inducible Prox1-CreERT2 x Casp8$^{fl/fl}$ mice (from hereon Casp8^{LECko}). Even though we saw activation of Prox-1-CreERT2 upon tamoxifen treatment (protocol according to Fig. 1A) when crossed to a Rosa26-mTmG reporter line (Fig.R1A), we did not see sufficient reduced expression of Casp8 in neither lung nor small intestinal LECs of Casp8^{LECko} mice isolated by flow cytometry (Fig. R3B-E). Thus, this low recombination precluded us from further analyzing this mouse line.*

In another attempt to investigate the same question, we generated PDGFb-CreERT2 x Casp8$^{fl/fl}$ mice (from hereon Casp8^{BECko}). Based on previous studies, the PDGFb-CreERT2 line only recombines in BECs in the intestine (Bernier-Latmani et al. 2015). Consistently, endogenous expression of PDGFb-EGFP was restricted to Lyve^{-} (blood), not in Lyve^{+} (lymphatic) ECs (Appendix Fig. S3). Using this mouse line, and following a similar tamoxifen treatment protocol as before, we detected reduced expression of Casp8 in BECs (new Appendix Fig. S3B). Casp8^{BECko} mice were viable during the whole observation period (30 days post tamoxifen treatment; new Fig. 5C). Furthermore, Casp8^{BECko} mice did not show histopathological signs of small bowel inflammation (new Fig.5 D-G) nor differences in blood vessel density and lacteal length in the small intestinal villi (new Fig.5 H-J) or lymphatic density in Peyer's Patches (new Fig.5 K,L). Hence, deletion of Casp8 in BECs is not

sufficient to cause disease, as compared to deletion of Casp8 in BECs and LECs as seen in the Casp8^{ECko} mice. The timeframe of revision, together with the required time to generate this mouse line, did unfortunately not allow for analysis at later timepoints.

In summary, the above-mentioned results suggest that deletion of Casp8 in BECs is not sufficient to induce small bowel inflammation. However, it does not exclude the possibility that Casp8 expression is required in both, BECs and LECs together, considering a potential crosstalk between the blood and lymphatic vascular system.

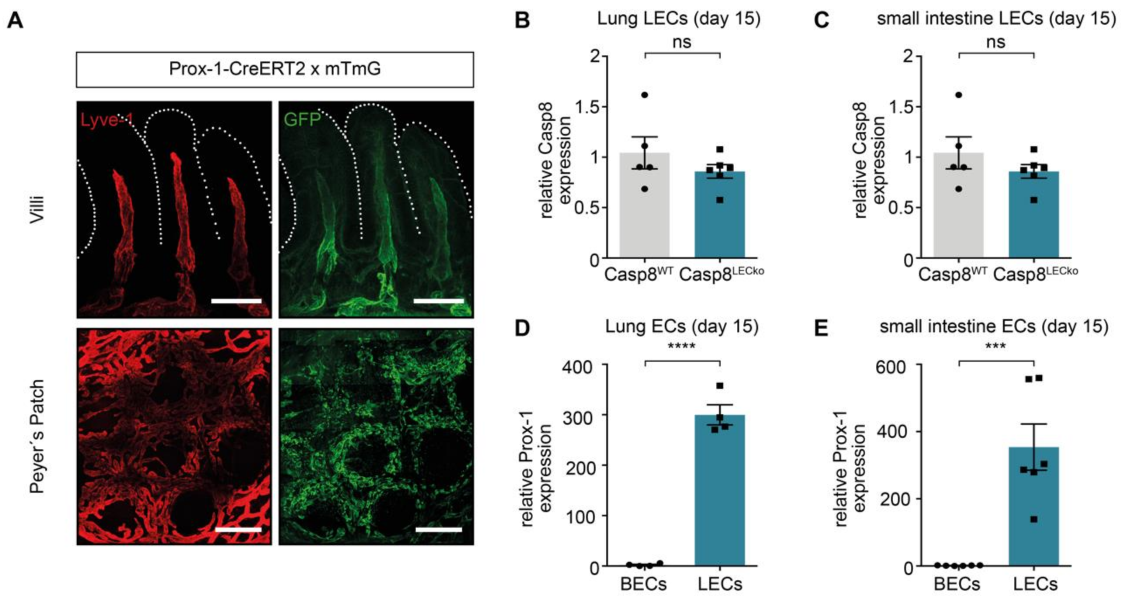

Fig. R3: Recombination efficiency in Prox-1-CreERT2 mice. (A) When crossed to the ROSA^{mTmG} reporter line, regular tamoxifen as used in this study (see manuscript, Fig. 1A) leads to GFP expression in the lymphatic vasculature in the intestine. (**B** and **C**) However, when crossed to Casp8$^{fl/fl}$ mice, we could not detect recombination in sorted LECs of lung (B) or small intestine (C). (**D** and **E**) Prox-1 expression as determined by qPCR analysis of sorted LECs compared to BECs confirms successful FACS gating. Scale bars: A)100µm (upper panel), 500µm (lower panel). Data shown as mean ± SEM. *** P <0.001, **** P <0.0001, ns: not significant.

Specific points

7. Fig. S2 B) Conversion of the Rosa-mTmG strain is often used as a proxy for deletion by a given deleter allele in a particular cell type. However, this should be considered with caution as Rosa mTmG conversion is no guarantee for successful deletion of other in particular multiple floxed alleles.

Answer: *We agree with the referee, and we did not intend to claim that Cre activity is a proxy for deletion of the Casp8 gene. Instead, we wanted to say that Cre recombinase is expressed and active in ECs of different vascular beds, and not restricted to the intestine. To clarify our statement, we are now writing "This phenotype was not due to a restricted expression of Cre-recombinase in intestinal ECs as analysis of Cdh5-Cre^{ERT2} x Rosa^{mTmG} reporter mice…".*

8. Fig. S2 C,D) Addition of qRT-PCR data is most commendable, however, inclusion of intestinal ECs would have been desirable.

Answer: *Thank you for the comment. As suggested, we now provide data of isolated ECs from small and large intestine of Casp8^{ECko} and control mice by flow cytometry. In both*

organs, Casp8 recombination efficiency, as determined by qPCR (new Appendix Fig. S1F, G), is around 85-90% in blood and 70-80% in lymphatic ECs.

9. Fig. S2 F) Staining for mG in the Lyve1 positive areas (LECs) appears weaker compared to the surrounding BECs. Why is that, given that the same reporter allele is activated in both cell types?

Answer: *Similar quantitative differences in labeling intensity between LECs and BECs have also been reported by others. For example, staining of CD31 and other vessel membrane markers, such as VEGFR2 and VEGFR3, though expressed in both EC types, seem to label the blood stronger than the lymphatic endothelium (Podgrabinska, Braun et al., 2002). One explanation might be post-transcriptional modifications (Podgrabinska et al., 2002). In addition, the membrane composition is different in the lymphatic and blood vasculature. Lymphatics are highly permeable, and especially lacteals present a high amount of discontinuous, button like cell junctions (Zhang, Zarkada et al., 2020). Thus, it could be that staining of a membrane-bound protein, such as membrane tagged GFP as in the mTmG reporter mouse (new Appendix Fig. S1), would reflect this effect of "membrane discontinuity".*

10. Legend to Fig. 3 (K) and (J) are mixed up, (K) should read (J) and describes Caspase 8 inhibition not deletion. In addition (K) should be inserted before the sentence starting: ..."Quantification of cell death...".
Fig. 2 and following Figs.: Time of analysis (early or late after Tamoxifen induction) could be indicated in the figures for easy reference as done in Fig.1.

Answer: *We thank the referee for carefully reading and pointing out these inconsistencies. They are now corrected. In addition, in the revised version of our manuscript, we have added more information of the timepoint of analysis in the figures, as suggested.*

We thank all reviewers for their insightful and constructive criticisms that have guided us in the production of a thoroughly revised version that we hereby resubmit. The revision entailed the inclusion of two new main Figures, several new panels in the old figures, four new Supplementary Figures and the selection of five expanded view figures. We hope that the revised version of the manuscript now meets the reviewer's expectations.

Referee #2

(Comments on Novelty/Model System for Author)

Several aspects of the analysis need to be further improved. These are outlined in the "Remarks to be sent to the authors".

(Remarks for Author)

Tisch et al. describe how endothelial-specific Casp8 deletion results in small intestinal enteropathy associated with inflammation and increased cell death, ultimately leading to increased lethality. This pathology is present only in the small intestine, although Casp8 is targeted in endothelial cells of all other organs. The authors further show that this phenotype is ameliorated either by deletion of MLKL or antibiotic treatment after weaning, indicating the crucial role of necroptosis and microbial responses in its aetiopathogenesis. This is an interesting paper that reveals an important homeostatic role for endothelial Casp8 and endothelial cells in general in the intestine. However, the molecular mechanisms underlying the phenotype described are not analyzed in-depth, while several aspects of the analysis need to be further improved to support the conclusions drawn.

We thank the reviewer for the positive evaluation of our study. Below we address point by point each of her/his comments.

Major Comments

1. The authors have performed several experiments to analyze the phenotype of increased lethality and enteropathy that the Casp8ECko mice display. However, there are several concerns/questions that need to be addressed:

- What is the extent of enteropathy in the small intestine? Can the authors provide low and high magnification images to better show this pathology?

Answer: *The pathological features of the enteropathy have been analyzed by an experienced pathologist (Dr. Carolin Mogler) and provided as enteropathy score (new Fig. 1F, G and previously Fig. 1F, H). To further explain and illustrate the strength of enteropathy, in the revised version of our manuscript we have complemented Fig.1 by providing low and high magnification pictures. How the enteropathy score is created is now explained in more detailed in the material and methods section. In line with this and the next question of referee #2, we have now also added exemplary pictures to the supplement (Appendix Fig.S7), to better illustrate the different parameters of the score.*

We always focused our histopathological analysis on the ileum (for reasons of consistency between experiments). With respect to the extent of the enteropathy: even though we found hemorrhagic Peyer's Patches evenly distributed along the proximal-distal axis at early disease stages, late disease manifested most strongly in distal areas in most mice (see Fig. R4 below). Of note: Peyer's Patches along the proximal-distal axis are immunologically distinct niches, with more tolerogenic Peyer's Patches in the proximal, vs. more pro-inflammatory Peyer's Patches in the distal small intestine (Esterhazy, Canesso et al., 2019). This might explain why disease manifests in the distal areas. We will comment on this point

in the results and methods section accordingly. For all vessel analysis in the villi at early disease stages, we used areas surrounding the most distal hemorrhagic Peyer's Patches.

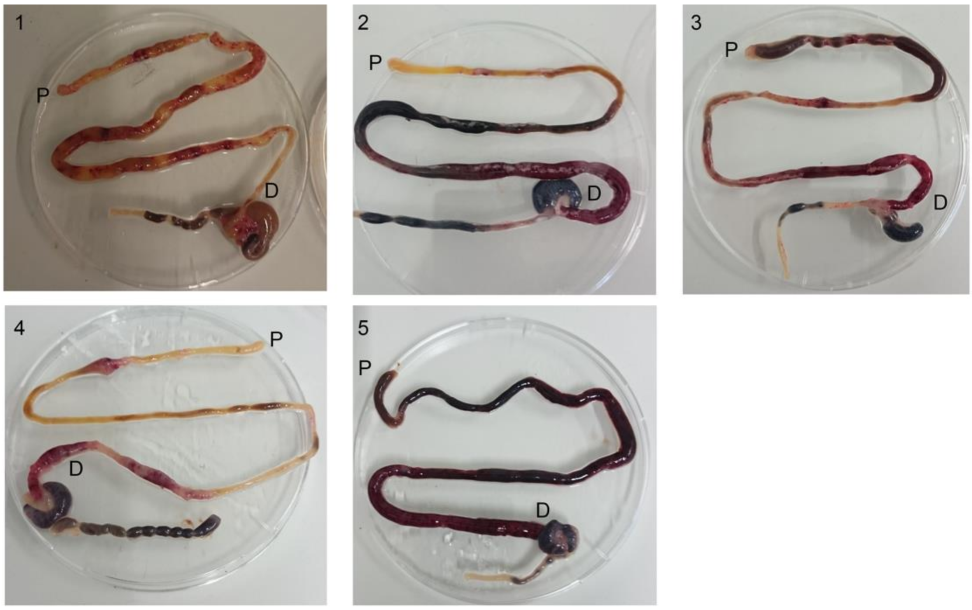

Fig. R4: Exemplary pictures of Casp8^{ECko} intestines at a late disease stage. P= proximal small intestine, D=distal small intestine.

2. Why do control Casp8wt mice in Fig. 1H have a positive enteropathy score that only doubles in the Casp8ECko mice?

Answer: *Indeed, Casp8^{WT} mice, in this quantification, showed a slight positive enteropathy score (old Fig. 1H). It can happen, likely depending on housing, during transport of animals to experimental facilities, etc. that also wildtype mice display mild signs of bowel irritation (personal communication with Dr. Carolin Mogler (pathologist)). We have asked Dr. Mogler to re-evaluate the samples (in a blinded fashion), which lead to the same result in this group of WT mice. However, please note that the control mice in other experiments e.g. Fig. 1F or Fig.5G did not show a positive enteropathy score. To further clarify this issue, we have now evaluated more mice and included four more Casp8^{WT}, and two more Casp8^{ECko} mice per genotype in this analysis. The new result better represents the true strength of the disease phenotype of Casp8^{WT} mice. All four control mice in the new analysis had the enteropathy score 0, as in other experiments, thus making our result even more significant (now $p<0.0001$).*

3. Can the authors show in supplementary figures the different parameters of the histopathological scores in control and experimental mice?

Answer: *We thank the reviewer for this suggestion. In the revised version of our manuscript, we provide exemplary pictures with further explanations from the different scores/disease strengths to visualize the different parameters that have been evaluated in the histopathology score (new Appendix Fig. S7 A,B). We provide similar information for the parameters evaluated in the DSS model (new Appendix Fig. S7 A,C).*

4. In Figure 1I how is the number of mucosal hemorrhages calculated (per mouse, per field, other)?

Answer: *Mucosal hemorrhages were calculated per mouse/per lesion. We now also indicate this information in the material and methods section.*

5. In Fig. 2G and 1G (g') the intestine seems to have lost entirely its structure. This is not obvious in other images (e.g. Fig. 2E, 2H). Which are more representative of the phenotype? Could images in 1G and 2G be the result of organ/organism death?

Answer: *In the previous Fig. 2G and 1G, we showed the phenotype at an advanced disease stage (called "late stage"), whereas previous Fig. 2E and 2H showed the phenotype at an earlier disease stage. This explains the differences in disease strength/tissue integrity that this referee refers to. We apologize for this confusion. In the revised version of our manuscript, we have clearly indicate the timepoint of analysis.*

Indeed, as this reviewer points out, the loss of structure could be the ultimate result of organ death/necrosis due to the lack of direct signaling from the diseased vasculature, hypoxia or severe inflammation in Casp8^{ECko} mice. We would like to highlight though that all mice were still alive at the timepoint of analysis, thus ruling out post-mortem affects.

6. The authors characterize the phenotype as having "features of chronic intestinal inflammation (reminiscent of IBD in humans)". It would be preferable for the interpretation of results if inflammatory cell infiltration is analyzed and quantified by FACS (including a more detailed analysis of immune cell populations).

Answer: *We agree with the reviewer that a detailed analysis of immune cell populations would be interesting, and we aimed to do so during the revision of this manuscript. Our aim was to analyze immune cells in the lamina propria by flow cytometry as follows: Relative proportions and absolute cell numbers per mg of tissue were determined for different subsets of immune cells: myelod cells (neutrophils, monocyte/macrophages and dendritic cells), innate lymphocytes (NK cells, ILC1s, ILC2s and ILC3s) and T cells (γδ+ T cells and αβ+ T cells, including NKT cells, CD8+ T cells and helper CD4+ T cells – Th1/2, Th17 and Foxp3+ regulatory T cells). Expression of PD-1 and CD44 on T cell was measured as indication of T cell activation. Different subsets of MHC II-expressing antigen presenting cells, including gut-resident DCs, defined by differential expression of CD8α and CD103, and CX3CR1+ macrophages, were assessed for the expression of costimulatory molecules (CD80 and CD86).*

Already in early diseased lamina propria, we observed a significant reduction in the number of recovered CD45+ cells in Casp8^{ECko} mice, and cell viability of the isolated population was also reduced (Fig. R2A.B). This technical limitation and low cell numbers prevented us from any further in-depth analysis, as we believe that the selective analysis of the alive population would mask or even falsify any potential difference between Casp8^{WT} and Casp8^{ECko} mice. As we also already showed a significant increase in TUNEL+ cells in the lamina propria in the initial manuscript (old Fig.3 G,H and Fig. S4 D,E now Fig.4 A,B and Appendix Fig. S2), we conclude that immune cell death occurs as a secondary event to vascular dysfunction. Though very interesting, this finding requires further investigation that lies beyond the scope of our study.

While we were not able to provide such a deep analysis of the immune cell populations, we already provided CD4, F4/80, MPO and NIMPR14 stainings at two different disease timepoints, together with histopathological analysis of H&E stained tissue sections (which also included scoring of inflammation and immune cell infiltration). In addition, we also provide a detailed cytokine analysis, which further supports our proposed model. This set of experiments is comparable to one of the landmark papers identifying Casp8 as key necroptosis regulator in the intestine, and Casp8 dependent small bowel inflammation in mice and humans (Gunther et al., 2011).

Altogether, we hope that this immune cell characterization will be sufficient for the first description of our newly presented mouse model in this study, where we focus on the vasculature. We will acknowledge the limitation of our study accordingly in the revised manuscript.

7. In Fig. 3A, it is not obvious what the authors refer to as tissue edema. Is it the relative height of intestinal epithelial cells? Have the authors measured this? What is the potential relationship with the phenotype observed?

Answer: *In the previous Fig. 3A, we referred to villus edema based on different observations: 1) the increased space observed between the vasculature and the epithelium, similar as it was done in other studies ((Cheat, Gerez et al., 2015, Sarac, Salman et al., 2015); 2) severe dilation of the remaining lymphatic vasculature at late disease stages (new Fig. EV3E (previously Fig. S4C); 3) the intestines seem to be swollen, enlarged in diameter, and fluid filled (see also Fig. 1D). We have confirmed these findings with a pathologist, and now provide additional H&E stainings to better show this pathology (new Fig. EV3B).*

As we observed regression of lymphatics, both in the villi and Peyer's Patches, we interpret these findings as a result of dysfunctional lymphatic vasculature.

8. Also, villi from Casp8ECko seem more elongated, which could be related to the part of the intestine that they come from. Villi from the same area of the small intestine should be shown. Are these images maximum projections of well-oriented villi?

Answer: *We are aware that the villi length decreases along the proximal-distal axis, with villi in the duodenum being significantly longer than the ileum. Therefore, we have consistently focused all our analysis on villi in the terminal ileum – distal jejunum, in areas surrounding the Peyer´s Patches. Images are maximum projections of well oriented villi. Of note, we are using wholemount preparations, thus avoiding bias due to sectioning artefacts. Taken together, we believe that the relative elongation of the villi in $Casp8^{ECko}$ mice compared to $Casp8^{WT}$ mice can be interpreted as a result of tissue swelling and edema.*

9. Since at this stage (Day 15), hemorrhages were mainly present in the Peyer's Patches, imaging of the vasculature of Peyer's Patches and analysis of immune cells there could be more meaningful.

Answer: *We thank the reviewer for this suggestion. In the revised version of our manuscript, we provide a detailed analysis of the vasculature, including TUNEL staining, in Peyer's Patches. At 15 days after the first tamoxifen treatment (early disease stage), not all Peyer's patches are equally affected, with some already showing hemorrhages and some others not presenting any macroscopic vascular phenotype (new Fig. 3A). Hence, we dissected and differentially analyzed Peyer's Patches with and without hemorrhages, hypothesizing that those without hemorrhages would represent an early step of disease development, potentially before EC death has occurred. This would allow us to track the development of the vascular defects and disease in more detail, from non- hemorrhagic, to early and severely hemorrhagic (diseased) stages.*

First, we analyzed the blood and lymphatic vasculature in wholemount stainings of Peyer's patches (new Fig.3 B,C). Lymphatic vessel density was analyzed based on Lyve-1 staining and showed that a reduction of lymphatic vessel density was an early event during disease development, even before hemorrhages had occurred (Fig. 3C, c'), and thus preceding changes in the blood vasculature. Severe hemorrhage formation, the lack of a good blood vessel specific marker in Peyer's Patches (CD31 also labels lymphatics), and strong tissue autofluorescence (potentially from immune cells) precluded the quantitative analysis of blood vessel density. However, morphological differences in the blood endothelium only became

apparent after the lymphatic density was already reduced (Fig. 3C, c'', c'''), suggesting that the lymphatic vasculature is affected earlier. This data is in line with the differential cell death analysis that we are now providing – showing TUNEL+ ECs in lymphatic, but not blood endothelial vasculature (new Fig. 5A).

Regarding the immune cells analysis, we chose a similar approach as for the lamina propria and aimed to analyze immune cells by flow cytometry: Peyer's Patches with and without hemorrhages at 15 days after the first tamoxifen treatment were dissected to characterize different immune cell populations (myeloid cells (neutrophils, monocyte/macrophages and dendritic cells), innate lymphocytes (NK cells, ILC1s, ILC2s and ILC3s) and T cells (γδ+ T cells and αβ+ T cells, including NKT cells, CD8+ T cells and helper CD4+ T cells – Th1/2, Th17 and Foxp3+ regulatory T cells). Expression of PD-1 and CD44 on T cell was measured as indication of T cell activation. Different subsets of MHC II-expressing antigen presenting cells, including gut-resident DCs, defined by differential expression of CD8α and CD103, and CX3CR1+ macrophages, were assessed for the expression of costimulatory molecules (CD80 and CD86).

Unfortunately, and similar to the lamina propria (see above) recovery of CD45+ cells from $Casp8^{ECko}$ lamina and Peyer's Patches was very low (Fig.R2 C.D), which might be in part due to immune cell death. Indeed, while death of lymphatic ECs was observed already in Peyer's Patches without or presenting small hemorrhages, death of other cells ((CD45+) TUNEL+ cells) in the Peyer's Patches was only observed at later events, indicating that immune cell death is a secondary event (new Fig. 5A). This technical limitation prevented us from further in-depth analysis. We will acknowledge this limitation in our revised manuscript.

Of note: the observation that immune cells are also affected in the Casp8 knockout mouse model is very interesting and will be further investigated in a follow up study. As we are (1) targeting ECs in our cell type specific Casp8 knockout mouse model, (2) as TUNEL+ cells first occur in the vasculature, and (3) as this phenotype is rescued in $Casp8^{ECko}/MLKL^{ko}$ mice, we believe that we provide sufficient data to show that EC dysfunction (which is the focus of our study) is preceding any change or activation in the immune system. Though it would be very exciting to characterize the timely events, and precise EC-immune cell crosstalk in more detail, we think that this technically challenging investigation lies beyond the scope of our study, which we will appropriately acknowledge in our discussion.

10. In Figure 3H, it is not clear what the authors refer to as stroma. Is it non-epithelial, non-endothelial cells? How do the authors explain increased Tunel+ cells in the epithelium and stroma? Could cell death be secondary to ensuing organ death rather than aetiopathogenic?

Answer: *Indeed, we refer to stroma as non-epithelial and non-endothelial cells, which we have now better indicated in the text. The increased TUNEL+ cells in the epithelium and stroma could be interpreted as i) a secondary event due to tissue necrosis or hypoxia; and or ii) as a consequence of changed or impaired angiocrine (=endothelial cell derived) signaling, especially as there are no hemorrhages yet in the villi, and EC death is rather minor (around 10%).*
Interestingly, while reviewing this manuscript, a new mouse model of lymphangiectasia has been described, and treatment with lymph fluid increases permeability of intestinal epithelial cell monolayers (Gonzalez-Loyola, Bovay et al., 2021). As we see lymphatic defects, together with tissue edema, this might be another explanation how vascular dysfunction impacts the epithelium and surrounding tissue in general.
In the revised version of our manuscript, we have now mentioned potential interpretations of the increased TUNEL+ cells in the epithelium and stroma, and also referred to this exciting new study in our manuscript.

11. In my opinion, the MLKLko data are the most supportive of a role for cell death/necroptosis in the phenotype and should be included in the main Figures.

Answer: *We thank the referee for his/her suggestion and now moved the MLKL^{ko} data to the new main Fig.4.*

12. Based on the in vitro data that show an increase in LECs' cell death after simultaneous inhibition of Casp8 and stimulation with TNF and LCL161, the authors suggest that LECs have a more important contribution in the disease development. Are there any in vivo data to support this conclusion, e.g. increased cell death in LECs? Experiments with tamoxifen-induced primary cells would be more representative of the in vivo data.

Answer: *In the revised version of our manuscript, we are now providing in vivo data addressing the referee's question. First, we performed TUNEL staining together with CD31/Lyve-1 staining in Peyer's Patches of Casp8^{WT} and Casp8^{ECko} mice. This analysis shows that only LECs, but not BECs are TUNEL positive (new Fig. 5A). We furthermore document the progression of LEC demise over the course of disease, by distinguishing between hemorrhagic and non-hemorrhagic Peyer's Patches in Casp8^{ECko} mice (new Fig. 3D-F). This analysis shows that regression of the lymphatic vasculature precedes defects in the blood vasculature.*

Furthermore, we generated PDGFb-CreERT2 x Casp8$^{fl/fll}$ mice, where in the intestine, Casp8 is solely deleted in BECs (from hereon Casp8^{BECko}). Casp8^{BECko} mice were viable during the whole observation period (30 days post tamoxifen treatment (new Fig. 5B, C), and neither developed vascular dysfunction nor signs of small bowel inflammation (new Fig. 5D-L). Hence, deletion of Casp8 in BECs is not sufficient to cause disease, as compared to deletion of Casp8 in BECs and LECs as in the Cdh5-CreERT2 x Casp8$^{fl/fl}$ mice.

We agree that it would be best to perform the cell death experiment with primary intestine BECs and LECs. Unfortunately, we and others (e.g. (Spadoni et al., 2015) have so far technically not succeeded to culture primary mouse intestinal ECs. Therefore, it is not possible to provide the analysis with tamoxifen-induced primary cells. During revision of this manuscript, we have now performed further experiments to compare the transcriptional properties of HDMECs/HDLECs and primary intestinal BECs/LECs in order to make sure that those cells would be a suitable model system. However, as elucidated in detail for referee #1, comment 2, we found substantial differences. We have thus decided to completely remove this data from the revised manuscript, and instead provide in vivo data where we differentially analyze cell death in BECs and LECs in the Peyer's Patches (see above).

13. Peyer's Patches should be analyzed in more detail in the TNF model to support common mechanisms with the spontaneous phenotype of Casp8ECko mice. High and low magnification images, analysis of the vasculature, Tunel assay for the identification of endothelial cell death, immune cell analysis should be performed. It would also be interesting to see if MLKL deletion rescues this phenotype too.

Answer: *We thank the reviewer for this suggestion. In the revised version of our manuscript, we now provide detailed analysis of the Peyer's Patches in Casp8^{WT}, Casp8^{ECko} and Casp8^{ECko}/MLKL^{ko} mice after intravenous TNF-α injection.*

For the analysis of the vasculature in the Peyer's Patches we performed wholemount preparations with CD31 and Lyve1 staining 24h after TNF-α injection. This analysis shows that hemorrhages and regression of the lymphatic vasculature already occur within this short time frame in Casp8^{ECko}, but neither in Casp8^{WT} nor Casp8^{ECko}/MLKL^{ko} mice (new Fig.6D-E), indicating that this phenotype is necroptosis dependent and is similar to the observed

phenotype in Casp8^{ECko} mice (without TNF-α injections) at early disease stages). In addition, the above-mentioned result is further supported by our second analysis of TUNEL-staining on Peyer's patch cryosections, showing TUNEL+ ECs localizing to the lymphatic vasculature in Casp8^{ECko}, but neither Casp8^{WT} nor Casp8^{ECko}/MLKL^{ko} mice upon TNF-α injection (new Fig. 6F).

Due to the technical limitations that we´ve already discussed previously, we did not further analyze immune cells by flow cytometry in this model and will acknowledge the limitations in the discussion of our revised manuscript.

14. The DSS experiments presented in Fig. 5 require additional analysis to be able to support the conclusions. In more detail:

- What is the clinical score of these mice? Do they show increased blood in their stool as a result of hemorrhage? Do they have decreased hematocrit? These data would help define the pathophysiological significance of the findings.
- The authors use a low dose of DSS. What happens when they give a more standard dose of 2-2.5%? Is there any difference in survival and/or clinical/histopathological scoring?

Answer: *Our aim by using the DSS model was to test whether the large intestine can also be affected in Casp8^{ECko} mice upon an explicit challenge, as the large intestine was not affected in Casp8^{ECko} mice* under basal conditions*. Our already shown results, also showing clinical scores (see old Fig.5 and new Appendix Fig. EV5), indicated that indeed, when challenged, the large intestine in Casp8^{ECko} develops more lesions, together with hemorrhages, upon DSS treatment in comparison to Casp8^{WT} mice. Altogether, this result supported our hypothesis that Casp8 expression in ECs is a pro-survival molecule required for tissue homeostasis.*

As this reviewer suggested, to complement this part of the study we now performed the DSS model with 2.5% DSS treatment (Fig.R5) and checked for visible blood in the stool of those mice. However, as blood in the stool is a common feature in the DSS model (Kiesler, Fuss et al., 2015)-independent of a vascular disease origin, it is difficult to see differences between WT and Casp8^{ECko} mice.

Upon *2.5% DSS treatment (Fig.R5 A), the overall weight loss was generally greater than after 1-1.5% DSS treatment, but survival and weight loss were not significantly different between Casp8^{WT} and Casp8^{ECko} mice (Fig. R5 B-C). Likewise, the histopathological score was in the same magnitude as after treatment with low dose DSS, and not significantly different between genotypes (Fig, R5 E-F). However, similar as with the low dose of DSS treatment, Casp8^{ECko}, but not Casp8^{WT} mice developed hemorrhages (Fig. R5D), indicating that Casp8^{ECko} are more susceptible to vascular dysfunction than Casp8^{WT}.*

Overall, as we did not detect major differences to the low DSS dose treatment, we did not further continue our investigations with this approach, but rather focused on complementing our initial analysis with the 1-1.5% DSS treatment regime, as requested in the following comments of the referee (see below).

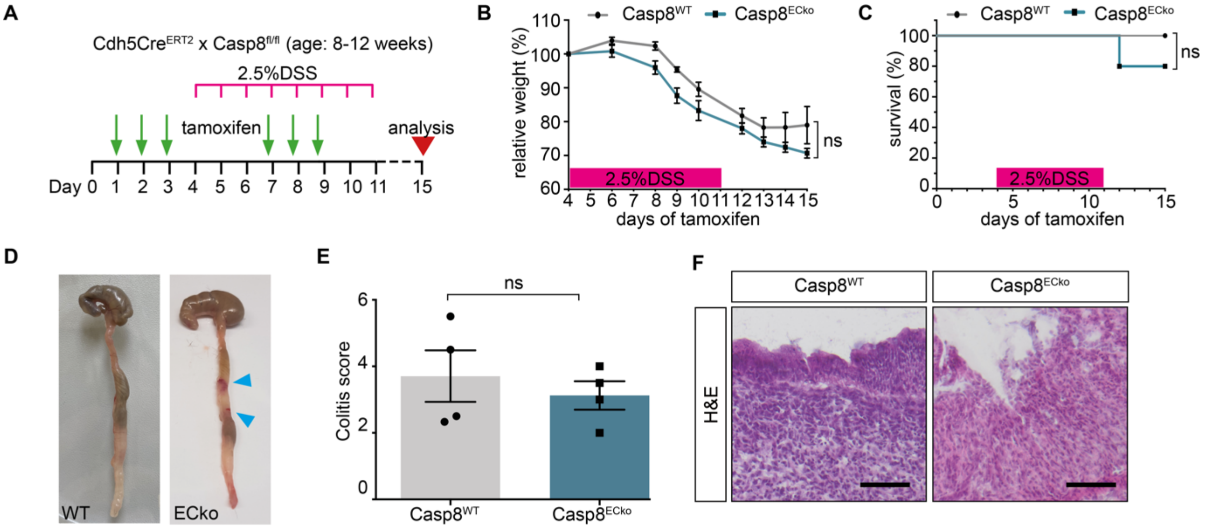

Fig. R5: Colitis development upon 2.5% DSS treatment. (**A**) Scheme of the applied treatment protocol. (**B** and **C**) No difference in weight (B; n= 4WT, 4-5ECko, curve comparison) or survival (C; n=4WT, 4-5ECko, Log-Rank test) could be seen between Casp8^{WT} and Casp8^{ECko} mice. (**D**) Similar to the 1-1-5% treatment protocol, Casp8^{ECko}, but not Casp8^{WT} mice developed hemorrhages. (**E** and **F**) No difference in the colitis score could be seen between Casp8^{WT} and Casp8^{ECko} mice (E; n= 4WT, 4ECko, two-tailed unpaired students t-test). All data is shown as mean ± SEM. ns: not significant. Scale bars: 100µm.

- Representative H&E stainings should be shown to better assess histologically the differences in the phenotype.

Answer: *We now provide H&E stainings showing the pathology in Casp8^{WT} and Casp8^{ECko} mice (new Fig. EV 5E). We would like to highlight that, as stated in the initial manuscript, Casp8^{WT} and Casp8^{ECko} mice do not per se show a histologically different phenotype (see colitis score in new Fig. EV 5F), only the number of lesions is increased (new Fig. EV 5H). In addition, as mentioned, we observed the formation of hemorrhages in Casp8^{ECko} mice (new Fig. EV 5I).*

- Do blood/lymphatic vessels show increased cell death in this model? Is there an increased expression of cytokines, chemokines, etc? Is there a difference in inflammation and inflammatory cell infiltration?

Answer: *In the revised version of our manuscript, we now provide TUNEL staining, combined with CD31/Lyve-1 staining (new Fig. EV 5K). Of note, tissue collection has been performed four days after acute DSS treatment, when regeneration already takes place, thus likely underestimating the amount of cell death during the initial wave of disease development. Taking this into consideration, and the overall mild outcome in our DSS model, we only rarely found TUNEL+ cells in general, thus precluding any meaningful quantitative analysis. However, only in Casp8^{ECko} mice, but not controls, we saw TUNEL+ lymphatic, but not blood ECs, thus confirming our hypothesis that LECs are the primary EC type requiring Casp8 expression to prevent necroptosis.*

We now also analyzed cytokine expression after low dose DSS treatment and could not detect major differences by qPCR analysis in the strength of inflammation between Casp8^{ECko} and Casp8^{WT} mice (new Fig. EV 5G), which is in line with the results of the histopathological score.

- This set of experiments is useful to show increased susceptibility to chemically-induced

colitis, but the data presented cannot support the conclusion that bacterial challenge in these mice disrupts the GVB and leads to intestinal disease and inflammation.

Answer: *We have down-tuned our initial statement and now write "indicating that Casp8^{ECko} mice are more susceptible to DSS induced colitis." instead. Still, the microbiota are a key part of the DSS induced Colitis model, where bacterial translocation induces inflammation upon chemical disruption of the epithelium (e.g. (Kiesler et al., 2015). Whereas control mice in a low-dose DSS model can cope with a pro-inflammatory environment, induced by bacteria (as they can in the small intestine under homeostatic conditions), Casp8^{ECko} mice cannot. Thus, it shows that such a translocation of bacteria in Casp8^{ECko} mice supports inflammation, formation of hemorrhages and colitis in the absence of Casp8.*

Nevertheless, we agree that it is only an indirect proof that the microbiota is required for disease development, as epithelial cell damage has to occur first in the DSS model, whereas it is secondary to EC damage in the small intestine of Casp8^{ECko} mice. We have thus down-tuned our initial statement according to the referee's comment and now only say that this model indirectly depends on the intestinal microbiota.

In recognition of a lack of in-depth analysis of this model and the fact that we could (despite the detection of small hemorrhages, LEC death and a mild increase in the number of lesions) not see strong clinical significance in this model, we have moved this data now to the new Fig. EV 5. We also feel that this is reflects better that this model was a tool, but not part of the focus of our paper.

15. The experiments showing the dependency of the phenotype on the presence of microbiota are very interesting. However, they are largely underexplored and there is no proposed mechanism. For example, how does the microbiota affect GVB?

Answer: *We apologize that this important point was not clear. We have now further elaborated on this topic in our revised manuscript. As we mentioned in our discussion, it has been previously shown that TNF-α production in CD11b+ myeloid cells is stimulated by the presence of microbiota (Naito, Iba et al., 2019). In the same paper, they show that antibiotic treatment reduces TNF-α production, and EC death in the absence of TAK1. We believe that a similar mechanism is true in our model, and that the microbiota indirectly affects the GVB by stimulating the intestinal immune system.*

Several studies have described how gut specific information about microbiota is conveyed and interpreted in Peyer's Patches already under steady state conditions, in healthy animals when the epithelial barrier is intact (Morikawa et al., 2016). For example, information about the intestinal microbiota is mainly transferred by active sampling of bacterial products from the intestinal lumen via specialized epithelial cells, and antigen presenting immune cells. For example, M-cells are specialized enterocytes that take up microbial products by transcytosis (e.g. phagocytosis, endocytosis, etc.) from the intestinal lumen before migrating specifically into Peyer's Patches, where those microbial products are presented to different immune cells (Kobayashi et al., 2019). Such a sampling and presentation of microbial material in the Peyer's Patches is also required for proper maturation of the intestinal immune system (Chung et al., 2012). Thus, microbiota information is presented in PPs in homeostatic conditions via different cellular and molecular mechanisms, with no infiltration of microbiota.

In line with this, we did not see a significant constant basal penetration of microbiota into the lamina propria (see this referees comment #16 and new Appendix Fig. S3C) in WT mice. This data is also consistent with other studies where microbiota is not detected across the epithelial barrier under steady state conditions (Gunther et al., 2015, Swidsinski et al., 2005). Furthermore, as depleting the microbiota with antibiotics before the initial tamoxifen treatment (and thus Casp8 deletion) fully protects the mice from disease development

further supports the idea that steady-state microbiota-immune cell communication is an upstream regulator of Casp8 function in ECs. We therefore propose the hypothesis that microbiota information is conveyed to Peyer's Patches in $Casp8^{ECko}$ mice in a similar way as in WT, indirectly via the immune system, without the need of bacterial translocation. It is then the signaling pathways activated in PPs that are different due to the lack of Casp8 in ECs. However, it is tempting to speculate that microbial products might also be directly presented to ECs, which requires further investigation. To address this question, we analyzed the expression of Toll-like receptors (TLRs; required to sense microbial products) in sorted intestinal BECs and L ECs (new Appendix Fig. S9). The main TLRs, TLR1,3 and 4 are all expressed in both populations.

To explore the possibility that a difference in microbiota populations between $Casp8^{WT}$ and $Casp8^{ECKO}$ might be a potential contributor to the phenotype, we also performed microbiome sequencing. Analysis of the sequence data at an early disease stage did not show any significant difference between $Casp8^{WT}$ and $Casp8^{ECko}$ mice, suggesting that microbiota differences are not preceding vascular dysfunction in $Casp8^{ECko}$ mice (new Appendix Fig. S11A, B).

In summary, we propose a model were microbiome-induced cytokine production signals to the vasculature. Under normal conditions, when ECs express Casp8, a pro-survival mechanism is in place to control vascular homeostasis. However, in the absence of Casp8, as cytokines, such as TNFα, are potent inducers of cell death in the absence Casp8, the endothelium in $Casp8^{ECko}$ mice becomes sensitive to necroptosis, which explains the initial appearance of hemorrhages in Peyer's Patches, home to the biggest immune cell reservoir.

16. Is there an increased influx of bacteria in the tissue potentially due to an altered epithelial barrier?

Answer: *In the revised version of our manuscript, we now provide FISH labeling of bacterial 16S rRNA in small intestine tissue of $Casp8^{WT}$ and $Casp8^{ECko}$ mice at a late disease timepoint. We could not detect significant bacterial translocation in $Casp8^{ECko}$ mice, even though there was a trend (new Fig. EV2 C,D). This is likely depending on focal lesions, rather than overall barrier break-down. This is supported by E-Cadherin staining to assess epithelial barrier integrity, which shows a defective epithelial barrier in severe intestinal lesions in $Casp8^{ECko}$ mice, whereas the barrier was intact in other, more healthy areas (new Fig. 2H). This is in line with an upregulated mucosal healing response (Fig. 2D), as well as increased Ki67 staining in intestinal crypts (Fig. 2E-G). Likely, active and enhanced mucosal healing maintains overall barrier integrity in $Casp8^{ECko}$ mice, thus avoiding significant bacterial translocation in diseased animals.*

17. Is this process TNF dependent as the authors hypothesize?

Answer: *Whereas vascular hemorrhages in Peyer's Patches are accelerated after intravenous injection of TNF-α, no other organ was affected in $Casp8^{ECko}$ mice (new Fig. EV 4G), at least on a histopathological level, as further discussed for referee 3, comment #4. This indicates that an additional signal, such as the microbiota (and microbiota regulated processes) are involved. In the revised version of our manuscript, we have now furthermore treated $Casp8^{ECko}$ mice with the TNF-α inhibitor Enbrel® (new Fig. 6G-K). TNF-α inhibition only partially prevented disease development in $Casp8^{ECko}$ mice, and 60% of the knockouts still succumbed with intestinal hemorrhages and pathology, similar to $Casp8^{ECko}$ mice that were treated with IgG control (new Fig. 6H-K). We therefore conclude that TNF-α contributes to but is not the cause of pathology in $Casp8^{ECko}$ mice.*

Minor Comments

18. For the short title, I would suggest: "Endothelial Casp8 prevents intestinal inflammation".

Answer: *We apologize for the spelling mistake in our title, which we corrected accordingly.*

19. More information is needed in the Methods sections. For techniques that are referenced elsewhere, a brief explanation would be helpful. Other specific information that is missing: primers and qRTPCR conditions for analysis of 16S bacterial rDNA, a vendor for recombinant mouse TNF, qRTPCR primers, and conditions for Fig. 2A-B.

Answer: *In the revised version of our manuscript, we are providing more detailed methodologic information, including the information requested by the referee.*

20. The number of mice in many in vivo experiments is quite small (3-4 mice). Have these experiments been repeated?

Answer: *We have carefully looked again at our data and here refer to all experiments that are with small group size (3-4 mice). Where feasible, we have included more mice now.*

1) Previous Fig. 1F: 3WT vs. 4 ECko from two independent experiments. p= 1.0, student´s t-test. In our opinion this data already convincingly shows that there is no histopathological significant small bowel inflammation phenotype at early disease stages. To further convince the referee, we have included more mice in this analysis now (new Fig. 1F: 6WT vs. 7 ECko, p=1.0).

2) Previous Fig. S5F,G. 3WT vs. 3 ECko p=0.5 student´s t-test. We agree that this group size in this particular key experiment is small and have now added more mice to this experiment (new Fig. 5F, H 4WT, 5ECko), which did not affect the results (p=0.29 student´s t-test). Taken together with the analysis of Casp8^{ECko}/MLKL^{ko} mice at a later timepoint after tamoxifen treatment, we believe this data is convincingly showing that there is no vascular defect in those mice.

3) Previous Fig. 6G. 4WT, 4 ECko, 4 ECko treated with antibiotics, adjusted p-value =0.0004 (Casp8^{WT} vs. Casp8^{ECko}); 0.0009 (Casp8^{ECko} vs. Casp8^{ECko} with antibiotics). We have now included 2WT and 2ECko treated with antibiotics. Inclusion of two more Casp8ECko treated with antibiotics lead to the detection of one statistical outlier in this group, which has been excluded, and thus leading to a total of 5 mice in the new Fig. 7G. Overall, this did only improve the significance of our previous graph (adjusted p-values = <0.0001 (Casp8^{WT} vs. Casp8^{ECko}); <0.0001 (Casp8^{ECko} vs. Casp8^{ECko} with antibiotics).

21. p.11: "restricted" seems more appropriate than "specific".

Answer: We have corrected this sentence accordingly.

22. Can the authors also show the levels of Casp8 deletion in small intestinal and colonic endothelial cells to exclude potential organ-specific differences that could explain the absence of a phenotype in the colon? Similarly, they should also show deletion efficiency in the skin.

Answer: *We have now specifically isolated BECs and LECs from small intestine and colon tissue of Casp8^{WT} and Casp8^{ECko} mice by FACS. By qPCR analysis, we show that the*

recombination efficiency in both cell types is high (~70-90% reduction of Casp8 expression) and comparable between both the small and large intestine (new Appendix Fig.S1 F, G). We have furthermore isolated CD31+ ECs by magnetic bead isolation from back skin samples and show that Casp8 expression is also reduced there by ~70% (new Appendix Fig.S1 E).

23. Colocalization of CD31/IsoB4 and GFP is not evident in the images provided in Fig. S2B. Split channels and higher magnification would help. A description of how the quantification was performed should also be added in the Methods.

Answer: *We are now providing higher magnifications of separated pictures of CD31/IsoB4 and /GFP staining. The quantification is expressed as GFP+ area per CD31 or IsoB4+ area, which we now also indicate in the material and methods section.*

24. In the manuscript text, it is indicated that Fig. 2C, D contains gene expression data, but in the figure legend, they are described as the results of the proteome profiler analysis. Which is correct?

Answer: *We apologize for the confusion: Fig.2 C, D shows selected clusters of the proteome profiler assay to old Fig.S3B (now Fig. EV 2B). We have clarified the information accordingly.*

25. Unaltered vessel permeability in the brain is stated in the text (p. 13), but not shown in any figure.

Answer: *We thank the reviewer for noticing this mistake and have referred accordingly to this graph now in the text (new Fig.3H).*

26. In Fig. S3A, the label is missing.

Answer: *Thank you for pointing it out. We have added the label accordingly.*

27. Fig. S6 should be explained in more detail in the text and more information about the analysis should be provided in the Methods and the Figure Legend. Can the authors identify clusters co-expressing the indicated genes?

Answer: *We have added an appropriate section in the methods of the revised manuscript, and more explanations in the results section and figure legend. Regarding co-expression of the indicated genes, as can be seen from the figure (now Appendix Fig. S4), all genes are co-expressed in ECs of testis, lung, muscle, intestine, liver and heart. However, Ripk3 expression seems to be absent in spleen brain and kidney, and is thus not co-expressed with Tnfr1, Casp8 and Mlkl in these organs. We have added a note about this finding in the figure legend.*

28. Can the authors explain why they use a different tamoxifen protocol in the psoriasis model?

Answer: *We apologize if this was not clear in the first version of our manuscript. If we had started the Imiquimod treatment after our original tamoxifen treatment protocol (so from day 10-15 after the first tamoxifen treatment), the development of skin inflammation would have overlapped with the development of intestine dysfunction (at day 15 after the first tamoxifen treatment, hemorrhages are already observed). We clearly wanted to separate those two processes from each other. Therefore, we decided to use a more condensed tamoxifen treatment protocol for this model. We consider this protocol equally efficient, as it has been frequently used and standardized in other studies using the Cdh5-Cre^{ERT2} recombinase*

(Chen, Liu et al., 2019, Zhang, Kim et al., 2020). We have added a sentence in the method section to avoid misunderstanding.

29. Reference to Fig. 5A-D is not correct in the text. Reference of Fig. S5E-F in p.17 should be changed to Fig. 5E-F.

Answer: *We thank the referee for careful reading and pointing out this mistake. As the order of figures has changed in the revised manuscript, we carefully double checked that all labeling is correct.*

30. In many cases, analyses of the two different time-points are interchangeable, making it difficult to follow the paper and make comparisons. An indication of the time-point of analysis in each panel would be helpful.

Answer: *We apologize for this. We have now clearly indicated the different time points of all analysis so that there is difficulty in following this in the results section.*

We thank all reviewers for their insightful and constructive criticisms that have guided us in the production of a thoroughly revised version that we hereby resubmit. The revision entailed the inclusion of two new main Figures, several new panels in the old figures, four new Supplementary Figures and the selection of five expanded view figures. We hope that the revised version of the manuscript now meets the reviewer's expectations.

Referee #3

(Remarks for Author)

In the present manuscript, Tisch and colleagues investigated the function of Caspase-8 in endothelial cells (EC). For this purpose, they generated tamoxifen inducible EC specific Caspase-8 knockout mice using Cdh5(PAC)-CreERT2. Casp8ECko mice succumbed to death, about 3 weeks after the first tamoxifen injection. Casp8 deletion was associated with small bowel inflammation, vascular dysfunction and hemorrhage in the small intestine, whereas all others organs remained unaffected. Furthermore, they provide evidence that this specific effect was TNF-R1-dependent, leading to cell death in form of necroptosis, as the additional constitutive deletion of MLKL, could completely reverse the phenotype.
Moreover, they showed that Inflammation alone was not sufficient to induce vascular defects and pathology in Casp8ECko mice, since other organs (skin or liver) were not differently affected in a psoriasis inflammation model or the systemic TNF-dependent SIRS model.
Finally, they could show that the intestinal microbiota is required to trigger the disease development in a gut-specific manner.
Overall the study is well conducted, the data clearly presented and the conclusions are mainly justified. However, the following issues should be taken into account.
The involvement of TNF in spontaneously induced pathogenesis remains to be experimentally proven. Since an additional sub-crossing with TNF-R1 deficient mice is certainly time consuming, treatment of the Casp8ECko mice with Etanercept could alternatively be performed in order to investigate the involvement of TNF in the spontaneous phenotype.

Answer: *We thank the reviewer for his positive comments on our manuscript.*

In the revised version of our manuscript, we have now treated Casp8^{ECko} mice with the TNF-α inhibitor Enbrel® (new Fig. 6G). TNF-α inhibition only partially prevented disease development in Casp8^{ECko} mice, and 60% of the mice still succumbed with intestinal hemorrhages and pathology similar to Casp8^{ECko} mice that were treated with IgG control (new Fig. 6H-K). We therefore conclude that TNF-α contributes to but is not the sole cause of pathology in Casp8^{ECko} mice. These results fit to the SIRS experiment and our hypothesis that TNF-α is an accelerator but not driver of pathology in Casp8^{ECko} mice, and that another gut specific factor (as we show: the microbiota) is required. Furthermore interesting, this data is in line with the phenotype of the epithelial Casp8 knockout (Gunther et al., 2011).

Moreover, a previous work from the group of Manolis Pasparakis (Schwarzer et al. 2020) suggested that next to TNF-R1 also ZBP1 could be involved in driving intestinal inflammation in human patients with mutations in the CASP8 gene, who were reported to develop severe forms of very early onset IBD that was refractory to anti-TNF treatment (Lehle et al., 2019). Do the authors have any evidence that ZBP1 is also relevant in their model?

Answer: *In the study of Schwarzer et al., the authors show that increased expression of ZBP1 in FADD epithelial cell specific knock-out mice (FADD^{IEC-KO}) correlates with increased colitis severity. To address the referee's question, we have now analyzed ZBP1 expression in isolated intestinal BECs and LECs of Casp8^{WT} and Casp8^{ECko} mice by qPCR (Fig. R6*

A,B). This data shows no significant difference between $Casp8^{WT}$ *and* $Casp8^{ECko}$ *mice*. Therefore, we do not have any evidence at this moment that ZBP1 plays a relevant role in our model. Although very interesting, we think that further investigations in this direction would go beyond the scope of our study.

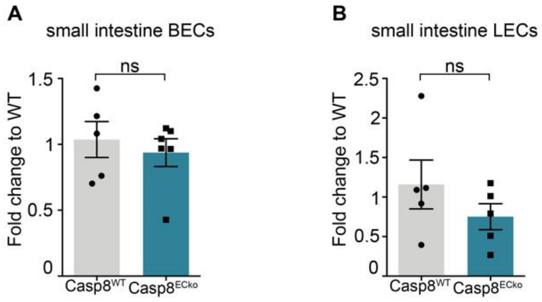

Fig. R6: ZBP expression in sorted intestinal BECs/LECS of $Casp8^{WT}$ and $Casp8^{ECko}$ mice. (**A** and **B**) mRNA expression levels of ZBP1 from qPCR analysis in sorted intestinal BECs (A) and LECs (B) (A; n= 5WT, 6ECko, B; n=5WT, 5ECko). All data is shown as mean ± SEM (unpaired students t-test).

Minor comments

1. Do Casp8ECko/MLKLko mice develop a later phenotype?

Answer: *In the literature, the majority of IBD mouse models develops the disease within the age of 3 weeks to 3 months (Neurath, 2012). On this basis, we now provide analysis of* $Casp8^{ECko}/MLKL^{ko}$ *mice at 3.5 months after the first tamoxifen treatment (Fig.4 of our revised manuscript). To ensure persistent recombination (in case Casp8 deficient ECs are replaced over time), we extended our tamoxifen treatment protocol (new Fig. 4C). We did not see differences in* $Casp8^{ECko}/MLKL^{ko}$ *mice viability compared to* $Casp8^{WT}/MLKL^{ko}$ *mice controls (new Fig. 4D). Furthermore, we do not find differences in vessel density (new Fig. 4I) or lacteal length (new Fig. 4K) in small intestinal wholemount preparations, as shown by CD31/Lyve-1 staining. We are now also providing wholemount analysis of the Peyer's Patch vasculature in* $Casp8^{ECko}/MLKL^{ko}$ *mice (new Fig. 4M, N). Also here, lymphatic vessel density is not significantly different to* $Casp8^{WT}/MLKL^{ko}$ *mice controls. Furthermore,* $Casp8^{WT}/MLKL^{ko}$ *mice do not develop histopathological signs of intestine inflammation (new Fig. 4O,Q). We therefore conclude that even at later timepoints after Casp8 deletion, co-deletion of MLKL is sufficient to prevent vascular dysfunction and the development of small intestine inflammation.*

2. Figure 2E-F: A counting of PCNA positive cells would be more useful than QPCR analysis to Ki-67, alternatively staining with statistical analysis. In addition, a zoom-in would be appropriate, the image is too small in order to see specific differences.

Answer: *In our revised manuscript, we have now replaced the PCNA staining with fluorescent Ki67 staining (new Fig. 2E) and quantified the percentage of Ki67+ epithelial cells per crypt (Fig. 2F). This analysis confirms that proliferation in the epithelium is already increased at an early disease stage in* $Casp8^{ECko}$ *mice, likely to maintain epithelial barrier function (please also see comment 16 of Referee #2 above).*

3. Which antibiotics were used. Are species-related differences to be expected?

Answer: *The following antibiotics, which are now also indicated in the material and methods section of our revised manuscript, were used: Ampicillin* $1gl^{-1}$ *(Ratiopharm), Neomycin* $1gl^{-1}$ *(Sigma), Meronem 0.5* gl^{-1} *(Friedrich-Eberth), Ciprofloxacin 0.5* gl^{-1} *(Sigma). This broad-spectrum antibiotic cocktail has been previously used to deplete the intestinal microbiota*

(Dannappel, Vlantis et al., 2014), and as shown by PCR analysis of 16S rDNA expression, we see a strong reduction of the most common bacteria phyla (new Fig. 7B). Taken together, we do not expect any species-specific difference.

4. In Figure 4E, the liver and kidney seems to be affected upon TNF treatment in WT but not in Casp8ECko mice. Do the authors have additional parameters regarding liver damage (e.g. serum transaminases, AST/ALT) or kidney damage (creatine kinase (CK))? In general, the images are too small. A zoom-in would be helpful.

Answer: *We apologize if the representative pictures for the liver and kidney shown in the initial manuscript gave the wrong impression that those tissues were affected after TNF-α injection. We considered again our collaborator (Dr. Carolin Mogler, pathologist) to discuss the liver and kidney phenotype of TNF-α treated $Casp8^{WT}$ and $Casp8^{ECko}$ mice. After careful analysis of more sections, we came again to conclude that the liver and kidney were not affected, at least (and this we have now acknowledged in the revised manuscript) on a histopathological level. We are aware that liver damage in the SIRS model has been previously observed (Duprez, Takahashi et al., 2011) . However, it is unlikely that damage would occur on a histological level at low dosage of TNF-α during the time window of observation (24h). Therefore, additional parameters (such as AST/ALT serum levels) would have been a better readout, as suggested by the reviewer. As we were focusing on the vasculature, in particular hemorrhage formation as a sign of EC damage, we did not assess those parameters. In the revised manuscript, we have now provided better representative H&E pictures of the liver and kidney, together with higher magnification pictures (zoom in specifically on vessels) of all organs evaluated.*

5. The order should be adjusted. In the text, however, 5G is described first, then 5D-F. Please adjust

Answer: *We have corrected the order of the figure accordingly (now Fig. EV5).*

References:

Cheat S, Gerez JR, Cognie J, Alassane-Kpembi I, Bracarense APFL, Raymond-Letron I, Oswald IP, Kolf-Clauw M (2015) Nivalenol Has a Greater Impact than Deoxynivalenol on Pig Jejunum Mucosa in Vitro on Explants and in Vivo on Intestinal Loops. *Toxins* 7: 1945-1961

Chen Q, Liu Y, Jeong HW, Stehling M, Dinh VV, Zhou B, Adams RH (2019) Apelin(+) Endothelial Niche Cells Control Hematopoiesis and Mediate Vascular Regeneration after Myeloablative Injury. *Cell Stem Cell* 25: 768-783 e6

Chung H, Pamp SJ, Hill JA, Surana NK, Edelman SM, Troy EB, Reading NC, Villablanca EJ, Wang S, Mora JR, Umesaki Y, Mathis D, Benoist C, Relman DA, Kasper DL (2012) Gut immune maturation depends on colonization with a host-specific microbiota. *Cell* 149: 1578-93

Dannappel M, Vlantis K, Kumari S, Polykratis A, Kim C, Wachsmuth L, Eftychi C, Lin J, Corona T, Hermance N, Zelic M, Kirsch P, Basic M, Bleich A, Kelliher M, Pasparakis M (2014) RIPK1 maintains epithelial homeostasis by inhibiting apoptosis and necroptosis. *Nature* 513: 90-4

Duprez L, Takahashi N, Van Hauwermeiren F, Vandendriessche B, Goossens V, Vanden Berghe T, Declercq W, Libert C, Cauwels A, Vandenabeele P (2011) RIP kinase-dependent necrosis drives lethal systemic inflammatory response syndrome. *Immunity* 35: 908-18

Esterhazy D, Canesso MCC, Mesin L, Muller PA, de Castro TBR, Lockhart A, ElJalby M, Faria AMC, Mucida D (2019) Compartmentalized gut lymph node drainage dictates adaptive immune responses. *Nature* 569: 126-+

Gonzalez-Loyola A, Bovay E, Kim J, Lozano TW, Sabine A, Renevey F, Arroz-Madeira S, Rapin A, Wypych TP, Rota G, Durot S, Velin D, Marsland B, Guarda G, Delorenzi M, Zamboni N, Luther SA, Petrova TV (2021) FOXC2 controls adult lymphatic endothelial specialization, function, and gut lymphatic barrier preventing multiorgan failure. *Sci Adv* 7

Gunther C, Buchen B, He GW, Hornef M, Torow N, Neumann H, Wittkopf N, Martini E, Basic M, Bleich A, Watson AJ, Neurath MF, Becker C (2015) Caspase-8 controls the gut response to microbial challenges by Tnf-alpha-dependent and independent pathways. *Gut* 64: 601-10

Gunther C, Martini E, Wittkopf N, Amann K, Weigmann B, Neumann H, Waldner MJ, Hedrick SM, Tenzer S, Neurath MF, Becker C (2011) Caspase-8 regulates TNF-alpha-induced epithelial necroptosis and terminal ileitis. *Nature* 477: 335-9

Hagerling R, Hoppe E, Dierkes C, Stehling M, Makinen T, Butz S, Vestweber D, Kiefer F (2018) Distinct roles of VE-cadherin for development and maintenance of specific lymph vessel beds. *EMBO J* 37

Kiesler P, Fuss IJ, Strober W (2015) Experimental Models of Inflammatory Bowel Diseases. *Cell Mol Gastroenterol Hepatol* 1: 154-170

Kobayashi N, Takahashi D, Takano S, Kimura S, Hase K (2019) The Roles of Peyer's Patches and Microfold Cells in the Gut Immune System: Relevance to Autoimmune Diseases. *Front Immunol* 10: 2345

Morikawa M, Tsujibe S, Kiyoshima-Shibata J, Watanabe Y, Kato-Nagaoka N, Shida K, Matsumoto S (2016) Microbiota of the Small Intestine Is Selectively Engulfed by Phagocytes of the Lamina Propria and Peyer's Patches. *PLoS One* 11: e0163607

Naito H, Iba T, Wakabayashi T, Tai-Nagara I, Suehiro JI, Jia W, Eino D, Sakimoto S, Muramatsu F, Kidoya H, Sakurai H, Satoh T, Akira S, Kubota Y, Takakura N (2019) TAK1 Prevents Endothelial Apoptosis and Maintains Vascular Integrity. *Dev Cell* 48: 151-166 e7

Neurath MF (2012) Animal models of inflammatory bowel diseases: illuminating the pathogenesis of colitis, ileitis and cancer. *Dig Dis* 30 Suppl 1: 91-4

Podgrabinska S, Braun P, Velasco P, Kloos B, Pepper MS, Skobe M (2002) Molecular characterization of lymphatic endothelial cells. *Proc Natl Acad Sci U S A* 99: 16069-74

Sarac F, Salman T, Gun F, Celik A, Gurler N, Abbasoglu SD, Olgac V, Saygili A (2015) Effect of probiotic supplementation on bacterial translocation in common bile duct obstruction. *Pediatr Surg Int* 31: 155-161

Spadoni I, Zagato E, Bertocchi A, Paolinelli R, Hot E, Di Sabatino A, Caprioli F, Bottiglieri L, Oldani A, Viale G, Penna G, Dejana E, Rescigno M (2015) A gut-vascular barrier controls the systemic dissemination of bacteria. *Science* 350: 830-4
Swidsinski A, Loening-Baucke V, Lochs H, Hale LP (2005) Spatial organization of bacterial flora in normal and inflamed intestine: a fluorescence in situ hybridization study in mice. *World J Gastroenterol* 11: 1131-40
Zhang F, Zarkada G, Yi S, Eichmann A (2020) Lymphatic Endothelial Cell Junctions: Molecular Regulation in Physiology and Diseases. *Front Physiol* 11: 509
Zhang S, Kim JY, Xu S, Liu H, Yin M, Koroleva M, Guo J, Pei X, Jin ZG (2020) Endothelial-specific YY1 governs sprouting angiogenesis through directly interacting with RBPJ. *Proc Natl Acad Sci U S A* 117: 4792-4801

24th Mar 2022

Dear Prof. Ruiz de Almodóvar,

Thank you for the submission of your manuscript to EMBO Molecular Medicine. I am pleased to inform you that we will be able to accept your manuscript pending the following final amendments:

1) Tables: Please move Table 1 and 2 to the Appendix and rename them to "Appendix Table S1 and S2". Also, correct their callouts in the text accordingly.
2) In the main manuscript file, please do the following:
- Correct/answer the track changes suggested by our data editors by working from the attached document.
- Make sure that all special characters display well.
- Add callouts for figure 6C and F.
- In M&M, a statistical paragraph should reflect all information that you have filled in the Authors Checklist, especially regarding randomization, blinding, replication.
- In the reference list, citations should be listed in alphabetical order. Where there are more than 10 authors on a paper, 10 will be listed, followed by "et al.". Please check "Author Guidelines" for more information. https://www.embopress.org/page/journal/17574684/authorguide#referencesformat
3) Appendix: Please move the "Appendix Supplementary Methods" to the main manuscript M&M section. Combine all appendix figures, figure legends and appendix tables into one "Appendix" PDF file with a table of content.
4) Movies: Please provide legends and zipp each movie file with corresponding legend. Rename movie files to Movie EV1 and 2 and update callouts in the main text.
5) Synopsis: Please check your synopsis text and image, revise them if necessary and submit their final versions with your revised manuscript. Please be aware that in the proof stage minor corrections only are allowed (e.g., typos).
6) For more information: There is space at the end of each article to list relevant web links for further consultation by our readers. Could you identify some relevant ones and provide such information as well? Some examples are patient associations, relevant databases, OMIM/proteins/genes links, author's websites, etc...
7) Source data: We encourage you to include the source data for figure panels that show essential data. Numerical data should be provided as individual .xls or .csv files (including a tab describing the data). For blots or microscopy, uncropped images should be submitted (using a zip archive if multiple images need to be supplied for one panel). Please check "Author Guidelines" for more information. https://www.embopress.org/page/journal/17574684/authorguide#sourcedata
8) As part of the EMBO Publications transparent editorial process initiative (see our Editorial at http://embomolmed.embopress.org/content/2/9/329), EMBO Molecular Medicine will publish online a Review Process File (RPF) to accompany accepted manuscripts. This file will be published in conjunction with your paper and will include the anonymous referee reports, your point-by-point response and all pertinent correspondence relating to the manuscript. Let us know whether you agree with the publication of the RPF and as here, if you want to remove or not any figures from it prior to publication. Please note that the Authors checklist will be published at the end of the RPF.
9) Please provide a point-by-point letter INCLUDING my comments as well as the reviewer's reports and your detailed responses (as Word file).

I look forward to reading a new revised version of your manuscript as soon as possible.

Yours sincerely,

Zeljko Durdevic

Zeljko Durdevic
Editor
EMBO Molecular Medicine

.

***** Reviewer's comments *****

Referee #1 (Comments on Novelty/Model System for Author):

1. The manuscript investigates the relevance of Caspase 8 expression in endothelial cells. Analysis includes a wide range of technologies from macroscopy, via immunofluorescence and histological staining to a proteome profiler analysis. 2. The connection between Caspase 8 function in ECs and inflammation of the small intestine has not been reported previously. 3. IBD and Morbus Crohn are significant health issues with so far poorly understood etiology. 4 Tissue specific LOF studies in mice are state of the art to address this type of question. There are no ehtical issues associated with this study.

Referee #1 (Remarks for Author):

The authors have thoroughly considered this reviewer' comments and included substantial additional data to answer the questions raised. The reviewer's comments are adequately discussed and the reviewer appreciates the effort of generating two additional mouse models aiming to dissect effects on BECs and LECs further. The expression analysis of sorted intestinal BECs and LECs is informative and inclusion of the resulting data commendable. In addition, significantly more details are now provided about the events in the Peyer's Patches, this additional information improved the manuscript and provides additional insights.

Referee #2 (Remarks for Author):

The authors have addressed my comments, clarified several points, and added new data that support their conclusions. To my opinion, the manuscript is significantly improved and suitable for publication in EMM.

Referee #3 (Remarks for Author):

The authors addressed all my comments adequately. Excellent paper.

The authors performed the requested editorial changes.

We are pleased to inform you that your manuscript is accepted for publication and is now being sent to our publisher to be included in the next available issue of EMBO Molecular Medicine.

EMBO Press Author Checklist

Corresponding Author Name: Prof. Carmen Ruiz de Almodóvar, Dr. Nathalie Tisch
Journal Submitted to: EMBO Molecular Medicine
Manuscript Number: EMM-2021-14121

USEFUL LINKS FOR COMPLETING THIS FORM
The EMBO Journal - Author Guidelines
EMBO Reports - Author Guidelines
Molecular Systems Biology - Author Guidelines
EMBO Molecular Medicine - Author Guidelines

Reporting Checklist for Life Science Articles (updated January 2022)
This checklist is adapted from Materials Design Analysis Reporting (MDAR) Checklist for Authors. MDAR establishes a minimum set of requirements in transparent reporting in the life sciences (see Statement of Task: 10.31222/osf.io/9sm4x). Please follow the journal's guidelines in preparing your manuscript.
Please note that a copy of this checklist will be published alongside your article.

Abridged guidelines for figures
1. Data
The data shown in figures should satisfy the following conditions:
- the data were obtained and processed according to the field's best practice and are presented to reflect the results of the experiments in an accurate and unbiased manner.
- ideally, figure panels should include only measurements that are directly comparable to each other and obtained with the same assay.
- plots include clearly labeled error bars for independent experiments and sample sizes. Unless justified, error bars should not be shown for technical replicates.
- if n<5, the individual data points from each experiment should be plotted. Any statistical test employed should be justified.
- Source Data should be included to report the data underlying figures according to the guidelines set out in the authorship guidelines on Data Presentation.

2. Captions
Each figure caption should contain the following information, for each panel where they are relevant:
- a specification of the experimental system investigated (eg cell line, species name).
- the assay(s) and method(s) used to carry out the reported observations and measurements.
- an explicit mention of the biological and chemical entity(ies) that are being measured.
- an explicit mention of the biological and chemical entity(ies) that are altered/varied/perturbed in a controlled manner.
- the exact sample size (n) for each experimental group/condition, given as a number, not a range;
- a description of the sample collection allowing the reader to understand whether the samples represent technical or biological replicates (including how many animals, litters, cultures, etc.).
- a statement of how many times the experiment shown was independently replicated in the laboratory.
- definitions of statistical methods and measures:
  - common tests, such as t-test (please specify whether paired vs. unpaired), simple χ2 tests, Wilcoxon and Mann-Whitney tests, can be unambiguously identified by name only, but more complex techniques should be described in the methods section;
  - are tests one-sided or two-sided?
  - are there adjustments for multiple comparisons?
  - exact statistical test results, e.g., P values = x but not P values < x;
  - definition of 'center values' as median or average;
  - definition of error bars as s.d. or s.e.m.

Please complete ALL of the questions below.
Select "Not Applicable" only when the requested information is not relevant for your study.

Materials

Newly Created Materials	Information included in the manuscript?	In which section is the information available? (Reagents and Tools Table, Materials and Methods, Figures, Data Availability Section)
New materials and reagents need to be available; do any restrictions apply?	Yes	Generated new mouse lines. Material and Methods

Antibodies	Information included in the manuscript?	In which section is the information available? (Reagents and Tools Table, Materials and Methods, Figures, Data Availability Section)
For **antibodies** provide the following information:<br>- Commercial antibodies: RRID (if possible) or supplier name, catalogue number and or/clone number<br>- Non-commercial: RRID or citation	Yes	Antibodies are described in Material and Methods.

DNA and RNA sequences	Information included in the manuscript?	In which section is the information available? (Reagents and Tools Table, Materials and Methods, Figures, Data Availability Section)
**Short novel DNA or RNA including primers, probes**: provide the sequences.	Yes	Primer sequences are available in Appendix Table S1

Cell materials	Information included in the manuscript?	In which section is the information available? (Reagents and Tools Table, Materials and Methods, Figures, Data Availability Section)
**Cell lines:** Provide species information, strain. Provide accession number in repository **OR** supplier name, catalog number, clone number, and/**OR** RRID.	Not Applicable	
**Primary cultures:** Provide species, strain, sex of origin, genetic modification status.	Not Applicable	
Report if the cell lines were recently **authenticated** (e.g., by STR profiling) and tested for mycoplasma contamination.	Not Applicable	

Experimental animals	Information included in the manuscript?	In which section is the information available? (Reagents and Tools Table, Materials and Methods, Figures, Data Availability Section)
**Laboratory animals or Model organisms:** Provide species, strain, sex, age, genetic modification status. Provide accession number in repository **OR** supplier name, catalog number, clone number, **OR** RRID.	Yes	Material and Methods
**Animal observed in or captured from the field:** Provide species, sex, and age where possible.	Not Applicable	
Please detail **housing and husbandry conditions**.	Yes	Material and Methods

Plants and microbes	Information included in the manuscript?	In which section is the information available? (Reagents and Tools Table, Materials and Methods, Figures, Data Availability Section)
**Plants:** provide species and strain, ecotype and cultivar where relevant, unique accession number if available, and source (including location for collected wild specimens).	Not Applicable	
**Microbes:** provide species and strain, unique accession number if available, and source.	Not Applicable	

Human research participants	Information included in the manuscript?	In which section is the information available? (Reagents and Tools Table, Materials and Methods, Figures, Data Availability Section)
If collected and within the bounds of privacy constraints report on age, sex and gender or ethnicity for all study participants.	Not Applicable	

Core facilities	Information included in the manuscript?	In which section is the information available? (Reagents and Tools Table, Materials and Methods, Figures, Data Availability Section)
If your work benefited from core facilities, was their service mentioned in the acknowledgments section?	Yes	Acknowledgements

Design

Study protocol	Information included in the manuscript?	In which section is the information available? (Reagents and Tools Table, Materials and Methods, Figures, Data Availability Section)
If study protocol has been **pre-registered, provide DOI in the manuscript**. For clinical trials, provide the trial registration number **OR** cite DOI.	Not Applicable	
Report the **clinical trial registration number** (at ClinicalTrials.gov or equivalent), where applicable.	Not Applicable	

Laboratory protocol	Information included in the manuscript?	In which section is the information available? (Reagents and Tools Table, Materials and Methods, Figures, Data Availability Section)
Provide DOI OR other citation details if **external detailed step-by-step protocols** are available.	Not Applicable	

Experimental study design and statistics	Information included in the manuscript?	In which section is the information available? (Reagents and Tools Table, Materials and Methods, Figures, Data Availability Section)
Include a statement about **sample size** estimate even if no statistical methods were used.	Yes	Material and Methods, Figure Legends
Were any steps taken to minimize the effects of subjective bias when allocating animals/samples to treatment (e.g. **randomization procedure**)? If yes, have they been described?	Yes	Material and Methods
Include a statement about **blinding** even if no blinding was done.	Yes	Material and Methods
Describe **inclusion/exclusion criteria** if samples or animals were excluded from the analysis. Were the criteria pre-established?<br><br>If sample or data points were omitted from analysis, report if this was due to attrition or intentional exclusion and provide justification.	Yes	Material and Methods
For every figure, are **statistical tests** justified as appropriate? Do the data meet the assumptions of the tests (e.g., normal distribution)? Describe any methods used to assess it. Is there an estimate of variation within each group of data? Is the variance similar between the groups that are being statistically compared?	Yes	Material and Methods

Sample definition and in-laboratory replication	Information included in the manuscript?	In which section is the information available? (Reagents and Tools Table, Materials and Methods, Figures, Data Availability Section)
In the figure legends: state number of times the experiment was **replicated** in laboratory.	Yes	Figure legends
In the figure legends: define whether data describe **technical or biological replicates**.	Yes	Figure legends

Ethics

Ethics	Information included in the manuscript?	In which section is the information available? (Reagents and Tools Table, Materials and Methods, Figures, Data Availability Section)
Studies involving **human participants**: State details of **authority granting ethics approval** (IRB or equivalent committee(s), provide reference number for approval.	Not Applicable	
Studies involving **human participants**: Include a statement confirming that **informed consent** was obtained from all subjects and that the experiments conformed to the principles set out in the WMA Declaration of Helsinki and the Department of Health and Human Services Belmont Report.	Not Applicable	
Studies involving **human participants:** For publication of **patient photos**, include a statement confirming that consent to publish was obtained.	Not Applicable	
Studies involving experimental **animals**: State details of **authority granting ethics approval** (IRB or equivalent committee(s), provide reference number for approval. Include a statement of compliance with ethical regulations.	Yes	In Materials and Methods
Studies involving **specimen and field samples:** State if relevant **permits** obtained, provide details of authority approving study; if none were required, explain why.	Not Applicable	

Dual Use Research of Concern (DURC)	Information included in the manuscript?	In which section is the information available? (Reagents and Tools Table, Materials and Methods, Figures, Data Availability Section)
Could your study fall under dual use research restrictions? Please check biosecurity documents and list of **select agents and toxins** (CDC): https://www.selectagents.gov/sat/list.htm	Not Applicable	
If you used a select agent, is the security level of the lab appropriate and reported in the manuscript?	Not Applicable	
If a study is subject to dual use research of concern regulations, is the name of the **authority granting approval and reference number** for the regulatory approval provided in the manuscript?	Not Applicable	

Reporting

The MDAR framework recommends adoption of discipline-specific guidelines, established and endorsed through community initiatives. Journals have their own policy about requiring specific guidelines and recommendations to complement MDAR.

Adherence to community standards	Information included in the manuscript?	In which section is the information available? (Reagents and Tools Table, Materials and Methods, Figures, Data Availability Section)
State if relevant guidelines or checklists (e.g., **ICMJE, MIBBI, ARRIVE, PRISMA**) have been followed or provided.	Yes	Material and Methods
For **tumor marker prognostic studies,** we recommend that you follow the **REMARK** reporting guidelines (see link list at top right). See author guidelines, under 'Reporting Guidelines'. Please confirm you have followed these guidelines.	Not Applicable	
For **phase II and III randomized controlled trials**, please refer to the **CONSORT** flow diagram (see link list at top right) and submit the CONSORT checklist (see link list at top right) with your submission. See author guidelines, under 'Reporting Guidelines'. Please confirm you have submitted this list.	Not Applicable	

Data Availability

Data availability	Information included in the manuscript?	In which section is the information available? (Reagents and Tools Table, Materials and Methods, Figures, Data Availability Section)
Have **primary datasets** been deposited according to the journal's guidelines (see 'Data Deposition' section) and the respective accession numbers provided in the Data Availability Section?	Not Applicable	
Were **human clinical and genomic datasets** deposited in a public access-controlled repository in accordance to ethical obligations to the patients and to the applicable consent agreement?	Not Applicable	
Are **computational models** that are central and integral to a study available without restrictions in a machine-readable form? Were the relevant accession numbers or links provided?	Not Applicable	
If publicly available data were reused, provide the respective **data citations in the reference list**.	Yes	Figure legend and Materials and Methods, reference list
